# Two-year effects of semaglutide in adults with overweight or obesity: the STEP 5 trial

**DOI:** 10.1038/s41591-022-02026-4

**Published:** 2022-10-10

**Authors:** W. Timothy Garvey, Rachel L. Batterham, Meena Bhatta, Silvio Buscemi, Louise N. Christensen, Juan P. Frias, Esteban Jódar, Kristian Kandler, Georgia Rigas, Thomas A. Wadden, Sean Wharton, the STEP 5 Study Group

**Affiliations:** 1 Department of Nutrition Sciences, University of Alabama at Birmingham, Birmingham, AL USA; 2 University College London Centre for Obesity Research, Division of Medicine, University College London, London, UK; 3 National Institute of Health Research, UCLH Biomedical Research Centre, London, UK; 4 Centre for Weight Management and Metabolic Surgery, University College London Hospital, London, UK; 5 Novo Nordisk A/S, Søborg, Denmark; 6 Unit of Clinical Nutrition, Policlinico University Hospital, Palermo, Italy; 7 Department of Health Promotion, Mother and Child Care, Internal Medicine and Medical Specialties, University of Palermo, Palermo, Italy; 8 National Research Institute, Los Angeles, CA USA; 9 Department of Endocrinology and Nutrition, Hospital Universitario QuironSalud Madrid, Universidad Europea de Madrid, Madrid, Spain; 10 Department of Bariatric Metabolic Surgery, St George Private Hospital, Kogarah, Sydney, Australia; 11 Department of Psychiatry, Perelman School of Medicine, University of Pennsylvania, Philadelphia, PA USA; 12 York University, McMaster University and Wharton Weight Management Clinic, Toronto, ON Canada

**Keywords:** Obesity, Drug therapy, Obesity

## Abstract

The STEP 5 trial assessed the efficacy and safety of once-weekly subcutaneous semaglutide 2.4 mg versus placebo (both plus behavioral intervention) for long-term treatment of adults with obesity, or overweight with at least one weight-related comorbidity, without diabetes. The co-primary endpoints were the percentage change in body weight and achievement of weight loss of ≥5% at week 104. Efficacy was assessed among all randomized participants regardless of treatment discontinuation or rescue intervention. From 5 October 2018 to 1 February 2019, 304 participants were randomly assigned to semaglutide 2.4 mg (*n* = 152) or placebo (*n* = 152), 92.8% of whom completed the trial (attended the end-of-trial safety visit). Most participants were female (236 (77.6%)) and white (283 (93.1%)), with a mean (s.d.) age of 47.3 (11.0) years, body mass index of 38.5 (6.9) kg m^–2^ and weight of 106.0 (22.0) kg. The mean change in body weight from baseline to week 104 was −15.2% in the semaglutide group (*n* = 152) versus −2.6% with placebo (*n* = 152), for an estimated treatment difference of −12.6 %-points (95% confidence interval, −15.3 to −9.8; *P* < 0.0001). More participants in the semaglutide group than in the placebo group achieved weight loss ≥5% from baseline at week 104 (77.1% versus 34.4%; *P* < 0.0001). Gastrointestinal adverse events, mostly mild-to-moderate, were reported more often with semaglutide than with placebo (82.2% versus 53.9%). In summary, in adults with overweight (with at least one weight-related comorbidity) or obesity, semaglutide treatment led to substantial, sustained weight loss over 104 weeks versus placebo. NCT03693430

## Abstract (web summary)

Results from the STEP 5 trial, testing semaglutide as an adjunct to behavioral interventions in adults with overweight or obesity, demonstrate sustained weight loss over a period of 104 weeks.

## Main

Behavioral intervention incorporating modifications in diet and physical activity remains the foundation of treatment for overweight and obesity. However, because behavioral intervention is often not associated with clinically meaningful and sustainable weight loss, pharmacotherapy is recommended as an additional tool for long-term weight management in people with a body mass index (BMI) of at least 30 kg m^–2^, or at least 27 kg m^–2^ in those with weight-related comorbidities^1^.

Semaglutide is a glucagon-like peptide-1 (GLP-1) analog approved for the treatment of type 2 diabetes (oral semaglutide and subcutaneous semaglutide) and for reducing the risk of cardiovascular events in people with type 2 diabetes and cardiovascular disease (subcutaneous semaglutide only)^2–5^. At a dose of 2.4 mg once-weekly, subcutaneous semaglutide was approved in the United States, Europe, the United Kingdom and Canada for weight management in adults with overweight (BMI ≥ 27 kg m^–2^ with at least one weight-related comorbidity) or obesity (BMI ≥ 30 kg m^–2^)^2–5^, based on results from the Semaglutide Treatment Effect in People with Obesity (STEP) clinical trial program. In the STEP 1 and 3 trials in participants without type 2 diabetes, average placebo-subtracted weight losses of 12.4% and 10.3%, respectively, were seen with semaglutide 2.4 mg at week 68 (refs. ^6,7^).

**Extended Data Fig. 1:**
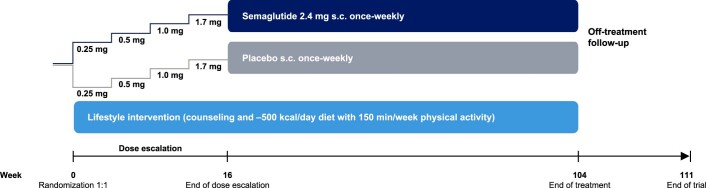
Trial design for STEP 5 clinical study. s.c., subcutaneous.

Previous studies in the STEP trial program have been limited to treatment durations of up to 68 weeks^6–8^. The 2-year STEP 5 study reported herein was conducted to evaluate the long-term effect of once-weekly subcutaneous semaglutide 2.4 mg compared with placebo, as an adjunct to behavioral intervention, on body weight and cardiometabolic risk factors, in adults with obesity (BMI ≥ 30 kg m^–2^), or with overweight (BMI ≥ 27 kg m^–2^) and at least one weight-related comorbidity, without diabetes (Extended Data Fig. 1). This phase 3, randomized, double-blind, placebo-controlled, multinational trial represents the longest study of the use of semaglutide for weight management to date. Co-primary endpoints were percentage change in body weight from baseline to week 104 and achievement of weight loss of at least 5% of baseline weight at week 104.

## Results

### Participants and treatment

**Fig. 1:**
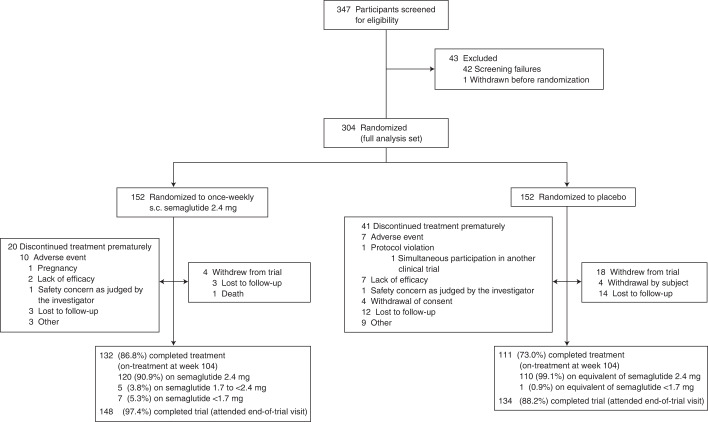
Flow chart of trial participants in the STEP 5 clinical trial. s.c., subcutaneous.

From 5 October 2018 to 1 February 2019, 304 participants were randomly assigned to semaglutide 2.4 mg (*n* = 152) or placebo (*n* = 152) and included in the full analysis set (all randomized participants according to the intention-to-treat principle). Observation periods included the in-trial period (that is, while in the trial, regardless of treatment discontinuation or rescue intervention) and the on-treatment period (with trial product). Overall, of 304 participants, 282 (92.8%) completed the trial (attended the end-of-trial safety visit), 272 (89.5%) had a body weight assessment at the end-of-treatment visit at week 104, and 243 (79.9%) adhered to treatment (were on-treatment at the end-of-treatment visit) (Fig. 1).

Demographics and baseline characteristics were similar between groups (Table 1). Most participants were female (236 (77.6%) of 304) and most were white (283 (93.1%) of 304). Mean age was 47.3 years. Mean body weight was 106.0 kg and mean BMI was 38.5 kg m^–2^.

**Table 1:** Baseline characteristics

Characteristic	Semaglutide (*n* = 152)	Placebo (*n* = 152)
Age, years	47.3 (11.7)	47.4 (10.3)
Sex^a^		
Female	123 (80.9%)	113 (74.3%)
Male	29 (19.1%)	39 (25.7%)
Race or ethnicity^b^		
White	141 (92.8%)	142 (93.4%)
Hispanic or Latino	18 (11.8%)	21 (13.8%)
Black or African American	7 (4.6%)	5 (3.3%)
American Indian or Alaska Native	2 (1.3%)	1 (0.7%)
Asian	2 (1.3%)	0 (0.0%)
Other	0 (0.0%)	4 (2.6%)
Body weight, kg	105.6 (20.8)	106.5 (23.1)
Body mass index, kg m^–2^	38.6 (6.7)	38.5 (7.2)
Waist circumference, cm	115.8 (14.3)	115.7 (15.5)
HbA_1c_	5.7% (0.3)	5.7% (0.4)
Fasting plasma glucose, mmol l^–1^	5.3 (0.5)	5.3 (0.6)
Fasting serum insulin geometric mean (CV), pmol l^–1^	87.6 (51.4); *n* = 143	88.1 (62.6); *n* = 146
Glycemic status^c^		
Normoglycemia	75 (49.3%)	88 (57.9%)
Prediabetes	77 (50.7%)	64 (42.1%)
Blood pressure, mmHg		
Systolic	126 (14)	125 (15)
Diastolic	80 (9)	80 (10)
Pulse, beats per min	73 (11)	72 (9)
Lipids geometric mean (CV), mmol l^–1^		
Total cholesterol	4.9 (20.9); *n* = 150	4.8 (18.3); *n* = 150
HDL cholesterol	1.2 (25.2); *n* = 150	1.2 (22.5); *n* = 150
LDL cholesterol	2.9 (30.1); *n* = 150	2.9 (25.7); *n* = 150
VLDL cholesterol	0.6 (46.5); *n* = 150	0.6 (47.4); *n* = 150
Free fatty acids	0.4 (57.2); *n* = 144	0.4 (63.3); *n* = 146
Triglycerides	1.3 (46.6); *n* = 150	1.2 (47.4); *n* = 150
C-reactive protein geometric mean (CV), mg l^–1^	4.8 (129.9)	3.8 (128.8)
Estimated glomerular filtration rate geometric mean (CV), ml min 1.73 m^–^^2^	95.7 (17.4)	92.9 (18.2)
Coexisting conditions at screening		
Dyslipidemia	58 (38.2%)	49 (32.2%)
Hypertension	56 (36.8%)	62 (40.8%)
Obstructive sleep apnea	27 (17.8%)	24 (15.8%)
Knee osteoarthritis	21 (13.8%)	25 (16.4%)
Nonalcoholic fatty liver disease	16 (10.5%)	15 (9.9%)
Asthma or chronic obstructive pulmonary disease	15 (9.9%)	17 (11.2%)
Polycystic ovarian syndrome^d^	10/123 (8.1%)	5/113 (4.4%)
Coronary artery disease	2 (1.3%)	3 (2.0%)

Data are *n* (%) or mean (s.d.) and include all patients in the full analysis set, unless indicated otherwise. There were no marked differences between treatment groups at baseline.

^a^Information on the sex of participants was collected by investigators by selecting from ‘male’ or ‘female’ on a case report form.

^b^Race and ethnic group were reported by the investigator. The category of ‘other’ includes any other ethnic group.

^c^Glycemic category was determined by investigators on the basis of available information (for example, medical records, concomitant medication, and blood glucose variables) and in accordance with American Diabetes Association criteria, which for prediabetes includes fasting plasma glucose levels of 100 mg dl^–1^ (5.6 mmol l^–1^) to 125 mg dl^–1^ (6.9 mmol l^–1^) or HbA_1c_ levels of 5.7–6.4% (39–47 mmol l^–1^), and for type 2 diabetes includes fasting plasma glucose levels of ≥126 mg dl^–1^ (7.0 mmol l^–1^) or HbA_1c_ levels ≥6.5% (48 mmol l^–1^)^29^. ^d^Percentage of female participants. CV, coefficient of variation; HDL, high-density lipoprotein; LDL, low-density lipoprotein; VLDL, very-low-density lipoprotein.

Two estimands were employed for the assessment of efficacy endpoints—estimands assess treatment efficacy from different perspectives and account for intercurrent events (for example, discontinuation of trial product or initiation of other weight loss interventions) and missing data differently. The ‘treatment policy’ estimand quantified the treatment effect for the in-trial period among all randomly assigned participants, regardless of treatment discontinuation or rescue intervention, based on the intention-to-treat principle, and was used as the primary analysis method. The ‘trial product’ estimand quantified the average treatment effect for the on-treatment period in all randomly assigned participants, assuming that the drug or placebo was taken as intended, and was used as the secondary analysis method (Methods).

### Efficacy endpoint results for the treatment policy estimand

**Fig. 2:**
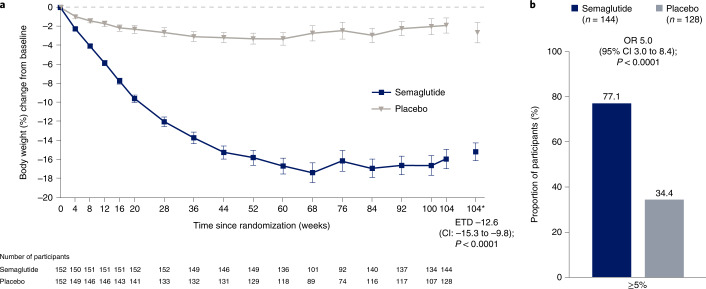
Comparison of body weight parameters for semaglutide versus placebo (co-primary endpoints; treatment policy estimand). **a**, Observed mean percentage change from baseline in body weight over time for participants in the full analysis set during the in-trial observation period (error bars are standard error of the mean; numbers below the panels are the number of participants contributing to the mean) and estimated treatment difference for the percentage change from baseline to week 104 in body weight based on the treatment policy estimand. **b**, Observed proportions of participants and OR for achieving weight loss of at least 5% from baseline at week 104 in the full analysis set during the in-trial observation period, based on the treatment policy estimand. *Estimated means in percent are from the primary analysis. The in-trial observation period was the time from random assignment to last contact with a trial site, regardless of treatment discontinuation or rescue intervention. The treatment policy estimand assesses treatment effect regardless of treatment discontinuation or rescue intervention; see Extended Data Fig. 6 for corresponding data for the trial product estimand (which assesses treatment effect assuming all participants adhered to treatment and did not receive rescue intervention). The change in body weight analysis was conducted with the use of the analysis-of-covariance method, with randomized treatment as a factor and baseline body weight as a covariate. The achievement of at least 5% weight loss analysis was conducted with the use of logistic regression, with the same factor and covariate. A multiple imputation approach was used for missing data. The results were accompanied by two-sided 95% CIs and corresponding *P* values (significance defined as *P* < 0.05). As co-primary endpoints, the analyses were controlled for multiple comparisons.

**Extended Data Fig. 2:**
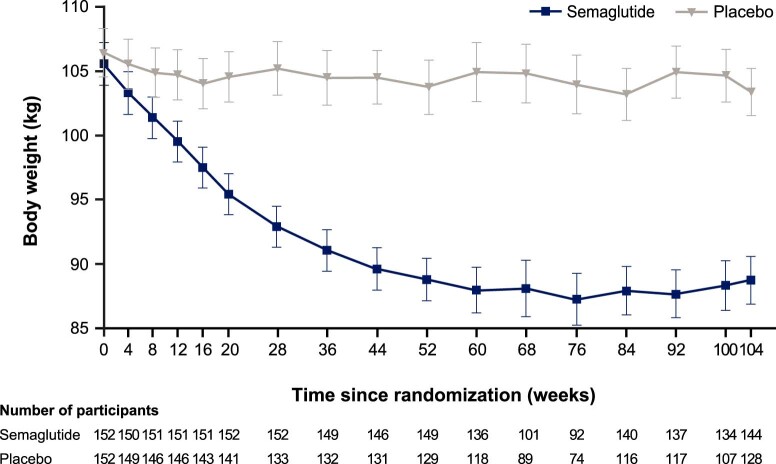
Body weight (kg) by week. Observed mean body weight (kg) over time for participants in the full analysis set during the in-trial observation period (from randomization to last contact with trial site, regardless of treatment discontinuation or rescue intervention). Error bars are standard error of the mean. Numbers below the panels are the number of participants contributing to the mean.

Mean observed change in body weight over time during the in-trial period is shown as percentage change in Fig. 2a and as absolute change (kg) in Extended Data Fig. 2. Based on the treatment policy estimand, the estimated mean (standard error (s.e.)) change in body weight from baseline to week 104 was –15.2% (0.9) with semaglutide and –2.6% (1.1) with placebo (co-primary endpoint; estimated treatment difference (ETD) –12.6 percentage points, 95% confidence interval (CI) –15.3 to –9.8, *P* < 0.0001). Semaglutide-treated participants, compared with placebo, were more likely to lose at least 5% of baseline body weight at week 104 (co-primary endpoint; odds ratio (OR) 5.0, 95% CI 3.0 to 8.4; *P* < 0.0001). At week 104, 111 (77.1%) versus 44 (34.4%) participants in the semaglutide and placebo groups, respectively, were observed to have achieved this endpoint (in-trial period data; among 144 participants for semaglutide and 128 for placebo) (Table 2 and Fig. 2b). As statistical superiority for both co-primary endpoints was demonstrated for semaglutide versus placebo, the prespecified criteria for a positive trial were met, indicating a significant benefit of semaglutide versus placebo.

**Table 2:** Co-primary, confirmatory secondary, and selected supportive secondary and exploratory trial endpoints^a^

	Semaglutide (*n* = 152)	Placebo (*n* = 152)	Treatment comparison (95% CI)^b^; *P* value for confirmatory analyses
**Co-primary endpoints**			
Body weight change from baseline to week 104, %	–15.2% (0.9)	–2.6% (1.1)	ETD –12.6 (–15.3 to –9.8); *P* < 0.0001
≥5% weight loss at week 104	111/144 (77.1%)	44/128 (34.4%)	OR 5.0 (3.0 to 8.4); *P* < 0.0001
**Confirmatory secondary endpoints**^c^			
≥10% weight loss at week 104	89/144 (61.8%)	17/128 (13.3%)	OR 7.2 (4.0 to 13.2); *P* < 0.0001
≥15% weight loss at week 104	75/144 (52.1%)	9/128 (7.0%)	OR 9.4 (4.4 to 20.0); *P* < 0.0001
Waist circumference—change from baseline to week 104, cm	–14.4 (0.9)	–5.2 (1.2)	ETD –9.2 (–12.2 to –6.2); *P* < 0.0001
Systolic blood pressure—change from baseline to week 104, mmHg	–5.7 (1.1)	–1.6 (1.2)	ETD –4.2 (–7.3 to –1.0); *P* = 0.0102
**Supportive secondary endpoints**^c^			
≥20% weight loss at week 104	52/144 (36.1%)	3/128 (2.3%)	OR 12.8 (3.9 to 41.9)
Body weight			
Change from baseline to week 104, kg	–16.1 (1.0)	–3.2 (1.2)	ETD –12.9 (–16.1 to –9.8)
Change from baseline to week 52, %	–15.6% (0.7)	–3.0% (0.7)	ETD –12.6 (–14.5 to –10.7)
Body mass index—change from baseline to week 104, kg m^–^^2^	–5.9 (0.4)	–1.6 (0.6)	ETD –4.3 (–5.7 to –2.9)
HbA_1c_—change from baseline to week 104, %	–0.4% (0.03)	–0.1% (0.03)	ETD –0.3 (–0.4 to –0.3)
Fasting plasma glucose—change from baseline to week 104, mmol l^–1^	–0.4 (0.05)	0.1 (0.06)	ETD –0.5 (–0.7 to –0.4)
Diastolic blood pressure—change from baseline to week 104, mmHg	–4.4 (0.9)	–0.8 (0.9)	ETD –3.7 (–6.1 to –1.2)
Fasting serum insulin—change from baseline to week 104, %^d^	–32.7%	–7.2%	Estimated relative percentage difference –27.4 (–39.3 to –13.3)
Lipids—change from baseline to week 104, %^d^			
Total cholesterol	–3.3%	1.4%	Estimated relative percentage difference –4.6 (–8.4 to –0.6)
HDL cholesterol	9.6%	8.1%	Estimated relative percentage difference 1.3 (–3.9 to 6.9)
LDL cholesterol	–6.1%	–2.7%	Estimated relative percentage difference –3.4 (–9.1 to 2.6)
VLDL cholesterol	–18.9%	3.3%	Estimated relative percentage difference –21.5 (–29.6 to –12.4)
Free fatty acids	0.3%	7.0%	Estimated relative percentage difference –6.2 (–21.2 to 11.6)
Triglycerides	–19.0%	3.7%	Estimated relative percentage difference –21.9 (–29.8 to –13.2)
C-reactive protein—change from baseline to week 104, %^d^	–56.7%	–7.8%	Estimated relative percentage difference –53.1 (–63.2 to –40.0)

Exploratory endpoints^c,e^		
Glycemic status at week 104 in participants with normoglycemia at baseline^f^		
Normoglycemia	70/71 (98.6%)	66/77 (85.7%)
Prediabetes	1/71 (1.4%)	10/77 (13.0%)
Type 2 diabetes	0/71 (0%)	1/77 (1.3%)
Glycemic status at week 104 in participants with prediabetes at baseline^f^		
Normoglycemia	59/74 (79.7%)	20/54 (37.0%)
Prediabetes	15/74 (20.3%)	32/54 (59.3%)
Type 2 diabetes	0/74 (0%)	2/54 (3.7%)
Change in lipid-lowering medication use at week 104^g^		
Increased	2/26 (7.7%)	5/29 (17.2%)
No change	19/26 (73.1%)	18/29 (62.1%)
Decreased	3/26 (11.5%)	1/29 (3.4%)
Stopped	2/26 (7.7%)	5/29 (17.2%)
Change in antihypertensive medication use at week 104^h^		
Increased	3/50 (6.0%)	14/61 (23.0%)
No change	31/50 (62.0%)	37/61 (60.7%)
Decreased	3/50 (6.0%)	5/61 (8.2%)
Stopped	13/50 (26.0%)	5/61 (8.2%)

Data are mean (standard error) or observed *n*/*N* (%) unless stated otherwise. All participants in the full analysis set are included in the treatment comparisons (that is, intention-to-treat analysis).

^a^Unless indicated otherwise, data are reported for the treatment policy estimand, which assesses treatment effect regardless of treatment discontinuation or rescue intervention; see Supplementary Table 1 for corresponding data for the trial product estimand (which assesses treatment effect assuming all participants adhered to treatment and did not receive rescue intervention). Continuous endpoint analyses were conducted with the use of the analysis-of-covariance method, with randomized treatment as a factor and baseline endpoint value as a covariate and a multiple imputation approach for missing data. Analyses of categorical endpoints were conducted with the use of logistic regression, with the same factor and covariate.

^b^The difference is the estimated treatment difference, odds ratio or estimated relative percentage difference between groups, as indicated.

^c^Confirmatory secondary endpoints were included in the statistical testing hierarchy. Supportive secondary and exploratory endpoint analyses were not included in the statistical testing hierarchy and analyses were not adjusted for multiplicity, and *P* values are therefore not reported for supportive secondary or exploratory endpoints.

^d^These parameters were initially analyzed on a log scale as estimated ratio to baseline (within treatment groups) and estimated treatment ratios (between treatment groups). For interpretation, these data are expressed as relative percentage change and estimated relative percentage difference between groups, respectively, and were calculated with the following formula: (estimated ratio − 1) × 100.

^e^Exploratory endpoints were assessed with descriptive statistics based on observed data.

^f^Glycemic category was determined by investigators on the basis of available information (for example, medical records, concomitant medication, and blood glucose variables) and in accordance with American Diabetes Association criteria, which for prediabetes includes fasting plasma glucose levels of 100 mg dl^–1^ (5.6 mmol l^–1^) to 125 mg dl^–1^ (6.9 mmol l^–1^) or HbA_1c_ levels of 5.7–6.4% (39–47 mmol l^–1^), and for type 2 diabetes includes fasting plasma glucose levels of ≥126 mg dl^–1^ (7.0 mmol l^–1^) or HbA_1c_ levels ≥6.5% (48 mmol l^–1^)^29^.

^g^Assessed in participants who received lipid-lowering medication between week 0 and week 104.

^h^Assessed in participants who received antihypertensive medication between week 0 and week 104. CI, confidence interval; EDT, estimated treatment difference; HDL, high-density lipoprotein; LDL, low-density lipoprotein; OR, odds ratio; VLDL, very-low-density lipoprotein.

**Extended Data Fig. 3:**
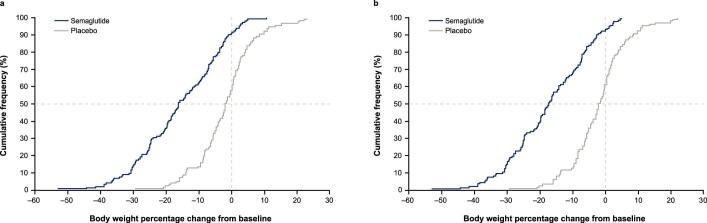
Cumulative distribution plot of change from baseline to week 104 in body weight. (**a**, **b**) Cumulative distribution plot of observed percentage change from baseline over time in body weight for participants in the full analysis set during the in-trial observation period* (a) and on-treatment observation period^†^ (b). *From randomization to last contact with trial site, regardless of treatment discontinuation or rescue intervention. ^†^During treatment with trial product (any dose of trial medication administered within the previous 2 weeks (that is, any period of temporary treatment interruption with trial product was excluded)).

Semaglutide-treated participants, compared with placebo, were also more likely to lose at least 10%, 15% or 20% of baseline body weight at week 104 (*P* < 0.0001 for the OR for the 10% and 15% thresholds (both were confirmatory secondary endpoints); the 20% threshold (a supportive secondary endpoint) was not part of statistical testing hierarchy). For the in-trial observation period, these weight loss thresholds were achieved by 89 (61.8%), 75 (52.1%) and 52 (36.1%) of 144 participants in the semaglutide group versus 17 (13.3%), nine (7.0%) and three (2.3%) of 128 participants in the placebo group, respectively (Table 2 and Extended Data Fig. 3 for cumulative distribution of change from baseline).

**Extended Data Fig. 4:**
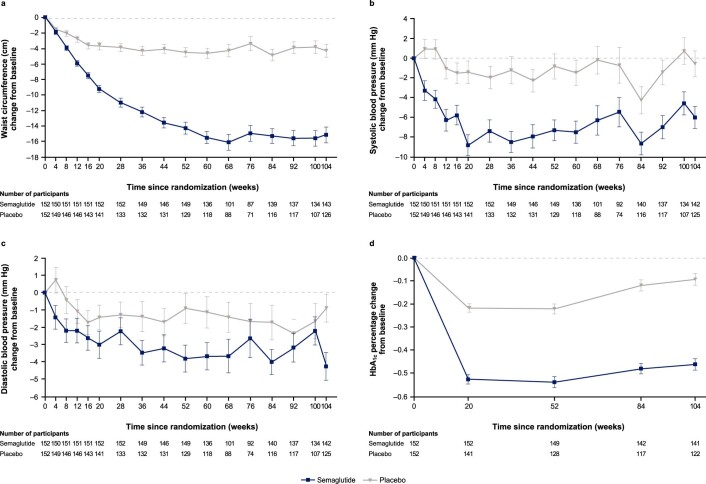
Comparison of change from baseline by week for selected cardiometabolic endpoints for semaglutide versus placebo. (**a**-**d**) Observed mean percentage change from baseline over time for participants in the full analysis set during the in-trial observation period in waist circumference (a), systolic blood pressure (b), diastolic blood pressure (c), and HbA_1c_ (d). Error bars are standard error of the mean; numbers below the panels are the number of participants contributing to the mean.

Semaglutide was associated with greater reductions from baseline to week 104 in waist circumference (–14.4 cm (0.9) with semaglutide versus –5.2 cm (1.2) with placebo; ETD –9.2 cm, 95% CI –12.2 to –6.2, *P* < 0.0001) and systolic blood pressure (–5.7 mmHg (1.1) with semaglutide versus –1.6 (1.2) with placebo; ETD –4.2 mmHg, 95% CI –7.3 to –1.0; *P* = 0.01) (both were confirmatory secondary endpoints; Table 2, Fig. 2 and Extended Data Fig. 4a,b). Compared with placebo, semaglutide also led to improvements in diastolic blood pressure, glycated hemoglobin (HbA_1c_), fasting plasma glucose, fasting serum insulin, C-reactive protein, total cholesterol, low-density lipoprotein cholesterol, very-low-density lipoprotein cholesterol and triglycerides (all were supportive secondary endpoints; Table 2 and Extended Data Fig. 4c,d).

**Extended Data Fig. 5:**
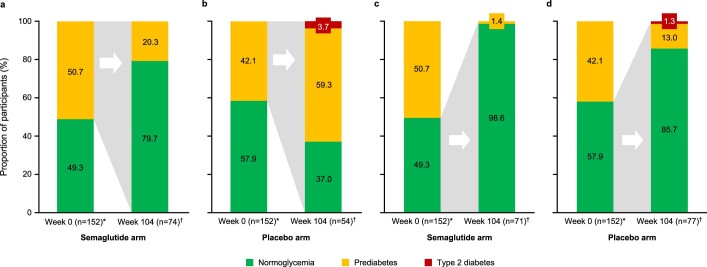
Shift from baseline to week 104 in glycemic status. (**a**-**d**) Observed data for participants in the full analysis set treated with semaglutide 2.4 mg (a, c) or placebo (b, d) during the in-trial period. As illustrated by the gray shading, the week 104 bars present results at this time point among the subgroups of participants with baseline prediabetes (a and b) or baseline normoglycemia (c and d). Glycemic category was determined by investigators on the basis of available information (for example, medical records, concomitant medication, and blood glucose variables) and in accordance with American Diabetes Association criteria,^30^ which for prediabetes includes fasting plasma glucose levels of 100 mg/dL (5.6 mmol/L) to 125 mg/dL (6.9 mmol/L) or HbA_1c_ levels of 5.7–6.4% (39–47 mmol/L), and for type 2 diabetes includes fasting plasma glucose levels of ≥126 mg/dL (7.0 mmol/L) or HbA_1c_ levels ≥6.5% (48 mmol/L). *Number of participants in the full analysis set. ^†^Number of participants with prediabetes (a and b) or normoglycemia (c and d) at baseline and evaluable data at week 104.

Of the participants with prediabetes at baseline who also had a glycemic status assessment at week 104, 59 (79.7%) of 74 treated with semaglutide reverted to normoglycemia at week 104, compared with 20 (37.0%) of 54 participants on placebo (an exploratory endpoint; Table 2 and Extended Data Fig. 5). Of the participants with normoglycemia at baseline who also had a glycemic status assessment at week 104, one (1.4%) of 71 treated with semaglutide had prediabetes at week 104, compared with 10 (13.0%) of 77 participants on placebo. Among participants with a week 104 assessment, none in the semaglutide group and three in the placebo group had type 2 diabetes at week 104 (one had normoglycemia at baseline and two had prediabetes at baseline). The proportion of participants with changes in the use of lipid-lowering and antihypertensive medication (among those receiving such medications during the trial) is reported in Table 2 (both were exploratory endpoints).

### Efficacy endpoint results for the trial product estimand

**Extended Data Fig. 6:**
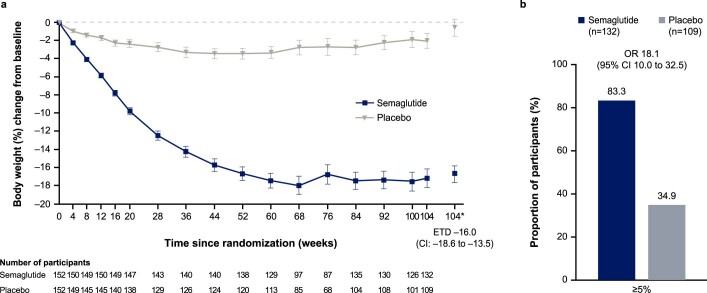
Comparison of body weight parameters for semaglutide versus placebo (trial product estimand). (**a**) Observed mean percentage change from baseline in body weight over time for participants in the full analysis set during the on-treatment observation period (error bars are standard error of the mean; numbers below the panels are the number of participants contributing to the mean) and estimated treatment difference for the percentage change from baseline to week 104 in body weight based on the trial product estimand. (**b**) Observed proportions of participants and odds ratio for achieving weight loss of at least 5% from baseline at week 104 in the full analysis set during the on-treatment observation period, based on the trial product estimand. *Estimated means in percent. A time point is considered as on treatment if any dose of trial product has been administered within the previous 14 days. The trial product estimand assesses treatment effect assuming all participants adhered to treatment and did not receive rescue intervention. CI, confidence interval; ETD, estimated treatment difference.

Mean observed change in body weight over time during the on-treatment period is shown in Extended Data Fig. 6a. For the trial product estimand, the estimated mean (s.e.) change in body weight from baseline to week 104 was –16.7% (0.9) with semaglutide and –0.6% (0.9) for placebo (ETD –16.0 percentage points, 95% CI –18.6 to –13.5). Semaglutide-treated participants, compared with placebo, were more likely to lose at least 5% of baseline body weight at week 104 (OR 18.1 (95% CI 10.0 to 32.5). At week 104, 110 (83.3%) versus 38 (34.9%) participants in the semaglutide and placebo groups, respectively, were observed to have achieved this endpoint (on-treatment period data; among 132 participants for semaglutide and 109 for placebo) (Supplementary Table 1 and Extended Data Fig. 6b). Results of analyses of the confirmatory and selected supportive secondary endpoints for the trial product estimand, are provided in Supplementary Table 1.

### Safety and tolerability

Adverse events leading to discontinuation of trial product were reported by nine participants (5.9%) in the semaglutide group and seven participants (4.6%) in the placebo group (Table 3).

**Table 3:** Adverse events

	Semaglutide (*n* = 152)			Placebo (*n* = 152)		
Adverse event	Participants	Events	Events per 100 patient-years	Participants	Events	Events per 100 patient-years
Any adverse event	146 (96.1%)	1606	532.3	136 (89.5%)	1004	374.8
Serious adverse events	12 (7.9%)	18	6.0	18 (11.8%)	20	7.5
Adverse events leading to trial product discontinuation	9 (5.9%)	12	4.0	7 (4.6%)	8	3.0
Gastrointestinal disorders leading to trial product discontinuation	6 (3.9%)	7	2.3	1 (0.7%)	1	0.4
Fatal events^a,b^	1 (0.7%)	1	0.3	0 (0.0%)		
Adverse events reported in at least 10% of participants^c^						
Nausea	81 (53.3%)	213	70.6	33 (21.7%)	53	19.8
Diarrhea	53 (34.9%)	108	35.8	36 (23.7%)	51	19.0
Constipation	47 (30.9%)	62	20.6	17 (11.2%)	26	9.7
Vomiting	46 (30.3%)	78	25.9	7 (4.6%)	8	3.0
Nasopharyngitis	24 (15.8%)	33	10.9	23 (15.1%)	31	11.6
Abdominal pain upper	22 (14.5%)	23	7.6	10 (6.6%)	13	4.9
Abdominal pain	20 (13.2%)	32	10.6	4 (2.6%)	14	5.2
Dyspepsia	20 (13.2%)	24	8.0	7 (4.6%)	12	4.5
Flatulence	20 (13.2%)	25	8.3	10 (6.6%)	11	4.1
Gastroenteritis	20 (13.2%)	28	9.3	4 (2.6%)	4	1.5
Influenza	20 (13.2%)	23	7.6	16 (10.5%)	19	7.1
Upper respiratory tract infection	20 (13.2%)	31	10.3	23 (15.1%)	30	11.2
Decreased appetite	17 (11.2%)	18	6.0	6 (3.9%)	6	2.2
Eructation	17 (11.2%)	21	7.0	1 (0.7%)	2	0.7
Headache	16 (10.5%)	36	11.9	16 (10.5%)	31	11.6
Back pain	15 (9.9%)	17	5.6	19 (12.5%)	20	7.5
Safety areas of interest^d^						
Gastrointestinal disorders^e^	125 (82.2%)	696	230.7	82 (53.9%)	252	94.1
Gallbladder-related disorders	4 (2.6%)	6	2.0	2 (1.3%)	2	0.7
Hepatobiliary disorders^e^	4 (2.6%)	6	2.0	2 (1.3%)	2	0.7
Cholelithiasis	3 (2.0%)	3	1.0	2 (1.3%)	2	0.7
Hepatic disorders	3 (2.0%)	4	1.3	3 (2.0%)	3	1.1
Acute pancreatitis	0 (0.0%)			0 (0.0%)		
Cardiovascular disorders^a^	17 (11.2%)	19	5.9	32 (21.1%)	45	14.9
Allergic reactions	23 (15.1%)	36	11.9	8 (5.3%)	9	3.4
Injection-site reactions	10 (6.6%)	17	5.6	15 (9.9%)	18	6.7
Malignant neoplasms^a^	2 (1.3%)	2	0.6	4 (2.6%)	4	1.3
Psychiatric disorders^e^	26 (17.1%)	33	10.9	25 (16.4%)	30	11.2
Acute renal failure	0 (0.0%)			0 (0.0%)		
Hypoglycemia	4 (2.6%)	10	3.3	0 (0.0%)		
Rare events	0 (0.0%)			1 (0.7%)	1	0.4
Overdose	0 (0.0%)			1 (0.7%)	1	0.4
COVID-19^f^	16 (10.5%)	17	5.6	8 (5.3%)	8	3.0

Data are *n* (%) of the safety analysis population (all randomized participants exposed to at least one dose of trial drug or placebo); since all participants received at least one dose of drug or placebo, the safety population is the same as the full analysis population. Data are for on-treatment adverse events, occurring during which any dose of semaglutide or placebo given within the previous 49 days (after excluding any temporary interruptions in taking trial intervention), unless indicated otherwise. Adverse events were classified by severity as mild (causing minimal discomfort and not interfering with everyday activities), moderate (causing sufficient discomfort to interfere with normal everyday activities) or severe (preventing normal everyday activities).

^a^In-trial observation period (the time from randomization to last contact with a trial site, regardless of treatment discontinuation or rescue intervention).

^b^Semaglutide group: one death due to acute myocardial infarction occurred in a participant who was a previous smoker with a medical history of hypertension, obstructive sleep apnea and dyslipidemia.

^c^Most common adverse events, by MedDRA preferred term, reported in at least 10% of participants in either treatment group.

^d^A number of safety focus areas were prespecified as being of special interest in the safety evaluation, based on regulatory feedback/requirements and therapeutic experience with GLP-1 receptor agonists. These preferred terms, identified through searches of MedDRA, were judged to be relevant for each of the safety focus areas.

^e^System organ class (for gallbladder-related disorders, ‘hepatobiliary disorders’ is the system organ class and ‘cholelithiasis’ is the preferred term).

^f^COVID-19 adverse events were classed as serious in one participant in the semaglutide group and in two participants in the placebo group; none required permanent discontinuation of the trial product. In addition, COVID-19 pneumonia was reported as an adverse event in one participant in the placebo group, which was classed as serious, and led to temporary interruption of the trial product. One participant in the placebo group reported asymptomatic COVID-19. COVID-19, coronavirus disease 2019; MedDRA, Medical Dictionary for Regulatory Activities, version 22.1.

**Extended Data Fig. 7:**
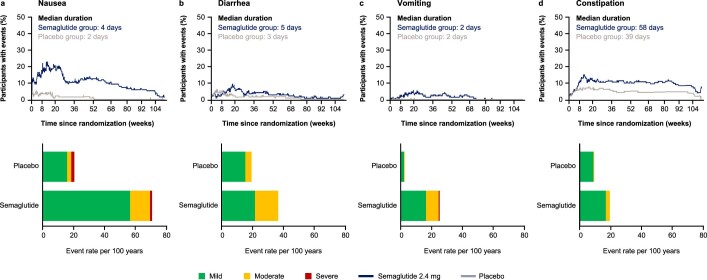
Prevalence and duration of gastrointestinal events by severity. (**a**-**d**) The proportion of participants receiving semaglutide or placebo who reported nausea (a), diarrhea (b), constipation (c), or vomiting (d) events classed as mild, moderate, or severe over the course of the treatment period. Data are from the on-treatment observation period (during treatment with trial product [any dose of trial medication administered within the previous 49 days (that is, any period of temporary treatment interruption with trial product was excluded)). Adverse events were classified by severity as mild (easily tolerated, causing minimal discomfort, and not interfering with everyday activities), moderate (causes sufficient discomfort and interferes with normal everyday activities), or severe (prevents normal everyday activities).

Gastrointestinal disorders, namely nausea, diarrhea, vomiting and constipation, were the most frequently reported adverse events and occurred in more participants treated with semaglutide than with placebo (125 (82.2%) of 152 versus 82 (53.9%) of 152, respectively) (Table 3). Most gastrointestinal adverse events were mild-to-moderate and transient, and such events led to permanent treatment discontinuation in six (3.9%) participants in the semaglutide group and one (0.7%) participant in the placebo group (Table 3 and Extended Data Fig. 7).

Serious adverse events were reported by 12 (7.9%) of 152 participants in the semaglutide group and 18 (11.8%) of 152 participants in the placebo group (Table 3). One death was reported in the semaglutide group and was considered by the independent external event adjudication committee to be unrelated to the trial product (Table 3). In the semaglutide versus placebo groups, gallbladder-related disorders were reported by four (2.6%) versus two (1.3%) participants and malignant neoplasms were reported by two (1.3%) versus four (2.6%), respectively (Table 3; details on malignant neoplasms are shown in Supplementary Table 2). There were no reports of pancreatitis in either treatment group. Additional safety variables are described in Table 3 and Supplementary Table 3. COVID-19 infection was reported by 16 (10.5%) of 152 participants in the semaglutide group versus eight (5.3%) of 152 participants in the placebo group, with very few cases in each group classed as serious and none requiring temporary or permanent interruption of semaglutide treatment.

## Discussion

In STEP 5, once-weekly treatment with semaglutide 2.4 mg as an adjunct to behavioral intervention in adults with overweight (with at least one weight-related comorbidity) or obesity led to a substantial initial reduction in weight, which plateaued after approximately week 60 and was maintained for the remainder of the study. At week 104, participants in the semaglutide group had achieved a mean weight loss of 15.2% from baseline—a difference of 12.6 percentage points versus placebo plus behavioral intervention. This weight loss is comparable to the mean reduction of 14.9% (placebo-corrected weight loss of 12.4 percentage points) seen at week 68 in the STEP 1 trial of semaglutide 2.4 mg versus placebo (both plus behavioral intervention)^7^. Thus, our findings indicate that the substantial weight losses reported during 68 weeks’ treatment with semaglutide 2.4 mg in prior STEP trials^6,7,9^ can be maintained with continued semaglutide treatment up to at least 104 weeks. The mean weight loss of ~15% achieved with semaglutide 2.4 mg at week 104 in STEP 5 exceeds weight loss reported at similar time points in trials with other pharmacotherapies for weight management in adults with overweight or obesity^10–14^.

Weight loss of ≥5%, a threshold widely used to indicate a clinically meaningful response to therapy^15^, was achieved by >75% of participants in the semaglutide group at week 104. Moreover, 61.8% of participants on semaglutide lost ≥10% of baseline weight, and over a third of participants had achieved at least 20% weight loss at week 104 in the semaglutide group. As was seen in prior studies^6,7,9,16^, while the vast majority of participants receiving semaglutide 2.4 mg had lost weight at the end of the STEP 5 study, a small proportion of participants experienced weight gain. We do not know how weight would have changed in these participants had they not been receiving the drug; notably, the proportion of patients with weight gain during the study was substantially higher in the placebo group. There is marked variability in weight change in patients on weight management treatments; the reason for this is still unclear and likely involves complex biological and societal influences.

Obesity is a chronic, relapsing disease that requires continuous effort to control^6,17^. With all nonsurgical interventions and to some extent with bariatric surgery, weight regain after initial weight loss is common^10–14,18–22^. In contrast to findings with behavioral^20–22^ and other pharmacological interventions^10,12,13^, the similar mean weight loss achieved with semaglutide 2.4 mg in STEP 5 at weeks 52 and 104 (–15.6% and –15.2%, respectively) suggests that, on average, there is minimal weight regain over 104 weeks when once-weekly semaglutide therapy is continued. When interpreted together with the findings of the STEP 4 withdrawal trial and STEP 1 off-treatment extension study, which both showed weight regain after semaglutide discontinuation (after 20 weeks’ treatment in STEP 4 and 68 weeks’ treatment in STEP 1)^23,24^, these results support the benefit of continued semaglutide treatment for sustained weight loss.

Prior 68-week trials in adults with overweight or obesity have reported cardiometabolic improvements with semaglutide 2.4 mg (refs. ^6,7,9,16^). Consistent with these findings, in STEP 5 semaglutide treatment improved a range of cardiometabolic risk parameters, including waist circumference, systolic and diastolic blood pressure, HbA_1c_ levels, total cholesterol, low-density lipoprotein cholesterol, very-low-density lipoprotein cholesterol and triglycerides. Collectively, these results indicate a beneficial effect of treatment on overall patient health. In addition, semaglutide treatment reduced C-reactive protein levels, a marker of systemic inflammation that is known to be elevated in patients with obesity^25,26^. The reduction in fasting insulin and glucose with semaglutide is indicative of an increase in insulin sensitivity. Similar to the findings of other studies in the STEP trial program^7,27^, exploratory outcomes showed that in the semaglutide group 80% of participants with prediabetes at baseline reverted to normoglycemia by the end of the trial (compared with 37% of those receiving placebo), while 99% of participants with normoglycemia at baseline maintained normoglycemia at the end of the trial (compared with 86% with placebo). These findings suggest a potential beneficial effect of semaglutide on glycemic status, but whether semaglutide treatment delays or prevents progression to type 2 diabetes requires confirmation. In the 68-week trials^7,9^, reductions in weight, waist circumference, blood pressure and HbA_1c_ appeared to plateau around week 60 with semaglutide. STEP 5 shows that the changes in these parameters were sustained through 104 weeks’ treatment.

The safety profile of semaglutide 2.4 mg in STEP 5 was consistent with that in other STEP program trials^6,7,9,16,23^, and with the GLP-1 receptor agonist class in general^28^. Gastrointestinal disorders were the most common adverse events with semaglutide, typically transient, of mild-to-moderate severity, occurring during dose escalation, and infrequently leading to treatment discontinuation.

Strengths of STEP 5 include the high rates of adherence to treatment and completion of the trial (which contributed to consistency in findings between the two estimands). Limitations include the low proportion of nonwhite participants and the preponderance of female participants. In addition, while the homogenous nature of the prescribed dietary intake deficit, physical activity goal and counseling frequency provided consistency, it may not fully reflect the need for approaches tailored to the health profiles of individuals or to different populations in clinical practice; however, beyond adherence to the stipulated criteria for counseling on diet and physical activity, behavioral intervention was delivered by each study site with no further direction, allowing a degree of local tailoring and aiding real-world applicability.

In conclusion, treatment with once-weekly subcutaneous semaglutide in conjunction with behavioral intervention in adults with overweight (with at least one weight-related comorbidity) or obesity (without diabetes) was associated with clinically ﻿﻿impactful and sustained weight loss of 15.2% at week 104, along with improvements in weight-related cardiometabolic risk factors.

## Methods

### Trial design and participants

This phase 3, randomized, double-blind, placebo-controlled study was conducted at 41 sites across five countries (Canada, Italy, Hungary, Spain and the United States), as described in a previous publication^8^ and listed in the Supplementary information. Most investigators specialized in endocrinology and internal medicine, with others specializing in family medicine, psychiatry and clinical psychology. The trial was conducted in accordance with the Declaration of Helsinki and Good Clinical Practice guidelines. The protocol was approved by independent ethics committees or institutional review boards at each study site (a redacted protocol is provided separately).

Participants were eligible to be included in the trial only if all of the following criteria applied:

- Informed consent obtained before any trial-related activities. Trial-related activities were any procedures that were carried out as part of the trial, including activities to determine suitability for the trial.
- Male or female, aged ≥18 years at the time of signing informed consent.
- BMI ≥ 30.0 kg m^–^^2^ or ≥27.0 kg m^–2^ with the presence of at least one of the following weight-related comorbidities (treated or untreated): hypertension, dyslipidemia, obstructive sleep apnea or cardiovascular disease.
- History of at least one self-reported unsuccessful dietary effort to lose body weight.

Participants were excluded from the trial if any of the following criteria applied:

#### Glycemia-related

- HbA_1c_ ≥ 48 mmol mol^–1^ (6.5%) as measured by the central laboratory at screening.
- History of type 1 or type 2 diabetes.
- Treatment with glucose-lowering agent(s) within 90 days before screening.

#### Obesity-related

- A self-reported change in body weight >5 kg (11 lbs) within 90 days before screening irrespective of medical records.
- Treatment with any medication for the indication of obesity within the past 90 days before screening.
- Previous or planned (during the trial period) obesity treatment with surgery or a weight loss device. However, the following were allowed: (1) liposuction and/or abdominoplasty, if performed >1 year before screening; (2) lap banding, if the band had been removed >1 year before screening; (3) intragastric balloon, if the balloon had been removed >1 year before screening; or (4) duodenal-jejunal bypass sleeve, if the sleeve had been removed >1 year before screening.
- Uncontrolled thyroid disease, defined as thyroid-stimulating hormone >6.0 mIU l^–1^ or <0.4 mIU l^–1^ as measured by the central laboratory at screening.

#### Mental health

- History of major depressive disorder within 2 years before screening.
- Diagnosis of other severe psychiatric disorder (for example, schizophrenia, bipolar disorder).
- A Patient Health Questionnaire-9 score of ≥15 at screening.
- A lifetime history of a suicidal attempt.
- Suicidal behavior within 30 days before screening.
- Suicidal ideation corresponding to type 4 or 5 on the Columbia-Suicide Severity Rating Scale within the past 30 days before screening.

#### General safety

- Presence of acute pancreatitis within the past 180 days before the day of screening.
- History or presence of chronic pancreatitis.
- Calcitonin ≥100 ng l^–1^ as measured by the central laboratory at screening.
- Personal or first-degree relative(s) history of multiple endocrine neoplasia type 2 or medullary thyroid carcinoma.
- Renal impairment measured as estimated glomerular filtration rate value of <15 ml min 1.73 m^–2^ as defined by KDIGO 2012 (ref. ^30^) by the central laboratory at screening.
- History of malignant neoplasms within the past 5 years before screening. Basal and squamous cell skin cancer and any carcinoma in situ were allowed.
- Any of the following: myocardial infarction, stroke, hospitalization for unstable angina or transient ischemic attack within the past 60 days before screening.
- Participant classified as being in New York Heart Association Class IV.
- Surgery scheduled for the duration of the trial, except for minor surgical procedures, in the opinion of the investigator.
- Known or suspected abuse of alcohol or recreational drugs.
- Known or suspected hypersensitivity to trial product(s) or related products.
- Previous participation in the trial. Participation was defined as signed informed consent.
- Participation in another clinical trial within 90 days before screening.
- Other person(s) from the same household participating in any semaglutide trial.
- Female who was pregnant, breast-feeding, or intended to become pregnant, or was of child-bearing potential and not using a highly effective contraceptive method.
- Any disorder, unwillingness or inability not covered by any of the other exclusion criteria which, in the investigator’s opinion, might have jeopardized the participant’s safety or compliance with the protocol.

### Randomization and masking

Randomization (1:1) to semaglutide 2.4 mg or placebo was done centrally by the clinical research organization (Parexel) in a double-blind manner using an interactive web-based response system (IWRS) with a fixed-size blocking schema, without stratification. The IWRS generated the randomization list and assigned patients to the next available treatment according to the randomization schedule. The IWRS allocated dispensing unit numbers for each patient, with the trial product dispensed by the site investigator or study coordinator at the trial site visits. The active product and corresponding placebo product were visually identical to maintain masking of participants and site staff. The people analyzing the data were blinded to treatment/group assignment until breaking the blinding at database lock.

### Procedures

Participants received subcutaneous semaglutide 2.4 mg or placebo once-weekly for 104 weeks, in addition to standard behavioral intervention, followed by 7 weeks without treatment. Semaglutide was initiated at 0.25 mg per week for the first 4 weeks via a pre-filled pen injector, escalating in a fixed-dose regimen every 4 weeks to reach the maintenance dose of 2.4 mg by week 16 (lower maintenance doses were permitted if participants were unable to tolerate 2.4 mg) (Extended Data Fig. 1). Behavioral intervention consisted of counseling by a dietitian or similarly qualified healthcare professional every 4 weeks via in-person visits or telephone on adherence to a reduced-calorie diet (500 kcal deficit a day relative to the energy expenditure estimated at randomization) and increased physical activity (150 minutes a week encouraged, for example, walking), both recorded daily (via a diary, app or other tools, which were reviewed during counseling sessions); beyond these criteria for behavioral intervention, no further standardization of behavioral intervention was applied across study sites. Participants discontinuing treatment prematurely remained in the trial and were encouraged to attend scheduled visits, particularly those at weeks 104 and 111.

Body weight, waist circumference and vital signs (systolic and diastolic blood pressure and pulse) were measured at baseline; these measurements were repeated every 4 weeks until week 20, and every 8 weeks thereafter, until week 100 and week 104 (within 3 days either side of scheduled visit day). These parameters were also measured at the end-of-trial visit at week 111 (within 5 days either side of scheduled visit day). Height was measured at screening. HbA_1c_, fasting plasma glucose, lipids and C-reactive protein were measured at baseline and weeks 20, 52, 84, and 104; electrocardiograms were also performed at these time points. Fasting serum insulin was measured at baseline and week 104. Physical examinations were performed at screening and weeks 52 and 104. Hematology and biochemistry laboratory parameters were measured at screening and weeks 20, 52, 84 and 104. Adverse events were recorded at each visit. Control of eating was assessed in a subset of participants from the United States and Canada; these results will be presented in a separate manuscript.

Given the emergence of COVID-19 in the second year of the study, trial visits were permitted to be conducted via telephone, during which counseling was provided and safety-related information was collected; endpoint assessments were not performed during telephone visits. Assessment data were collected at the next possible in-person visit.

### Outcomes

Co-primary endpoints were percentage change in body weight from baseline to week 104 and achievement of weight loss of at least 5% of baseline weight at week 104. These were tested first in the statistical testing hierarchy, followed by the confirmatory secondary endpoints, which were tested in the following order: achievement of weight loss of at least 10% or 15% at week 104; and change from baseline to week 104 in waist circumference and systolic blood pressure.

Supportive secondary endpoints were not included in the statistical testing hierarchy and were: achievement of weight loss of ≥20% at week 104; change from baseline to week 104 in body weight (in kg), BMI, HbA_1c_, fasting plasma glucose, fasting serum insulin, diastolic blood pressure, lipids (total cholesterol, high-density lipoprotein cholesterol, low-density lipoprotein cholesterol, very-low-density lipoprotein cholesterol, free fatty acids and triglycerides) and C-reactive protein; change from baseline to week 52 in body weight (percentage change and kg change), BMI and waist circumference; and achievement of weight loss of ≥5%, ≥10%, ≥15% and ≥20% at week 52.

Exploratory endpoints reported herein include change from baseline to week 104 in glycemic category, antihypertensive medication use and lipid-lowering medication use. Glycemic category (normoglycemia, prediabetes or type 2 diabetes) was determined by investigators on the basis of available information (for example, medical records, concomitant medication, and blood glucose variables) and in accordance with American Diabetes Association criteria^30^, which for prediabetes includes fasting plasma glucose levels of 100 mg dl^–1^ (5.6 mmol l^–1^) to 125 mg dl^–1^ (6.9 mmol l^–1^) or HbA_1c_ levels of 5.7–6.4% (39–47 mmol l^–1^), and for type 2 diabetes includes fasting plasma glucose levels of ≥126 mg dl^–1^ (7.0 mmol l^–1^) or HbA_1c_ levels ≥6.5% (48 mmol l^–1^). The allowance for investigators to use all available information (for example, concomitant medication) to assess glycemic category was primarily included to account for scenarios in which glucose-lowering medications were initiated during the trial that would confound glycemic category assessment if based purely on fasting plasma glucose or HbA_1c_ levels (for example, if a patient developed diabetes during the study and received a glucose-lowering drug that resulted in their glucose level being below the American Diabetes Association threshold for type 2 diabetes diagnosis). Additional exploratory endpoints for which data are not reported were: permanent discontinuation of trial product between baseline and week 104; time to permanent discontinuation of trial product; and Control of Eating Questionnaire scores from the four domains and 19 individual items (applicable for United States and Canada only).

Safety endpoints included the number of treatment-emergent adverse events and serious adverse events, assessed between baseline and week 111; and change from baseline to week 104 in pulse, amylase, lipase and calcitonin. An independent external event adjudication committee reviewed cardiovascular events, acute pancreatitis and deaths.

### Statistical analysis

A sample size of 300 participants provided an effective power of at least 96% for the two co-primary endpoints, and at least 43% for all confirmatory secondary endpoints, which were tested in a predefined hierarchical order (Supplementary Table 4). The two co-primary endpoints were analyzed independently of each other, and for the trial to be considered to be positive (indicating a significant benefit of semaglutide versus placebo), statistical superiority for both co-primary endpoints was required to be demonstrated.

Efficacy endpoints were analyzed using the full analysis set (all randomized participants according to the intention-to-treat principle). Safety endpoints were analyzed using the safety analysis set of all randomized participants exposed to at least one dose of randomized treatment. Observation periods included the in-trial period (that is, while in the trial, regardless of treatment discontinuation or rescue intervention) and the on-treatment period (with trial product). All results from statistical analyses of confirmatory endpoints were accompanied by two-sided 95% CIs and corresponding *P* values (significance defined as *P* < 0.05). Supportive secondary endpoint analyses were not controlled for multiple comparisons and should not be used to infer definitive treatment effects.

Two estimands were employed to assess treatment efficacy from different perspectives and accounted for intercurrent events and missing data differently, as described in a previous publication^31^. The treatment policy estimand quantified the treatment effect among all randomly assigned participants, regardless of treatment discontinuation or rescue intervention (participants in trial; intention to treat). This estimand was used to assess the superiority of semaglutide versus placebo for the co-primary and confirmatory secondary endpoints in a predefined hierarchical order.

For the treatment policy estimand, continuous endpoint analyses were conducted with the use of the analysis-of-covariance method, with randomized treatment as a factor and baseline endpoint value as a covariate. Analyses of categorical endpoints were conducted with the use of logistic regression, with the same factor and covariate. A multiple imputation approach was used to handle missing data^31^, with imputation based on available data from participants in the same treatment arm with the same treatment status (on-treatment or discontinued). Imputation was performed using a linear regression model, with sex, baseline BMI and timing of last observation as factors, and baseline value and last observation value as covariates. One thousand complete datasets were generated for analysis, with results combined using Rubin’s formula.

The trial product estimand addressed the average treatment effect in all randomly assigned participants, assuming that the drug or placebo was taken as intended (participants on treatment). For the trial product estimand, continuous endpoint analyses were conducted using a mixed model for repeated measures with randomized treatment as a factor and baseline endpoint value as a covariate. Analyses of categorical endpoints were conducted with the use of logistic regression, with categorization for missing data based on values predicted from the mixed model for repeated measures. Analyses of endpoints for the trial product estimand were not adjusted for multiplicity.

Statistical analyses were performed using SAS version 9.4 (SAS Institute Inc.). Additional details on analytic methods per endpoint are in Supplementary Table 4. Exploratory endpoints were assessed with descriptive statistics based on observed data.

The trial is closed and completed. The study is registered with ClinicalTrials.gov, NCT03693430.

### Reporting summary

Further information on research design is available in the Nature Research Reporting Summary linked to this article.

## Online content

Any methods, additional references, Nature Research reporting summaries, source data, extended data, supplementary information, acknowledgments, peer review information; details of author contributions and competing interests; and statements of data and code availability are available at 10.1038/s41591-022-02026-4.

## Supplementary information

Supplementary Information List of investigators in the STEP 5 trial and Supplementary Tables 1–4.

Reporting Summary

Supplementary Data 1 Study protocol.

Supplementary Data 2 ICMJE forms.

## Data Availability

Data will be shared with bona fide researchers submitting a research proposal approved by the independent review board. The research proposal must outline: the scientific rationale and relevance of the proposed research; a short lay summary intended for public disclosure; research methodology and data; statistical analysis plan and publication plan. Data must not be used for commercial purposes. Data will be made available after research completion, and approval of the product and product use in the European Union and the USA. Individual participant data will be shared in datasets in a de-identified and anonymized format. Access request proposals can be found at novonordisk-trials.com.
