# Supplementary material for: Two-year effects of semaglutide in adults with overweight or obesity: the STEP 5 trial
Source: Nat Med. 2022 Oct 10;28(10):2083–91. doi: 10.1038/s41591-022-02026-4 (PMC9556320; doi:10.1038/s41591-022-02026-4)
Supplement: Supplementary file 1 — List of investigators in the STEP 5 trial and Supplementary Tables 1–4. [file 41591_2022_2026_MOESM1_ESM.pdf]

nature medicine

Article

https://doi.org/10.1038/s41591-022-02026-4

Two-year effects of semaglutide in adults with overweight or obesity: the STEP 5 trial

In the format provided by the authors and unedited

Supplementary information

Contents

List of investigators in the Semaglutide Treatment Effect in People with Obesity (STEP) 5 trial 2

Supplementary tables 3

Supplementary Table 1 | Co-primary, confirmatory secondary, and selected supportive secondary and exploratory trial endpoints (trial product estimand).* 3

Supplementary Table 2 | Malignant neoplasms by system organ class and preferred term (in-trial).* 6

Supplementary Table 3 | Supportive secondary safety endpoints (on-treatment).* 7

Supplementary Table 4 | Statistical analysis methodology: analysis and imputation methods to address the treatment policy and trial product estimands for the primary and confirmatory secondary endpoints in the statistical testing hierarchy. 8

List of investigators in the Semaglutide Treatment Effect in People with Obesity (STEP) 5 trial

Canada: Ronald Akhras (Centre Medical Acadie, Montreal), Adam Blackman (Toronto Sleep Institute, Toronto), Daniel Landry (G.A. Research Associates Ltd., Moncton), Derek Lowe (Ocean West Research Clinic, Surrey), Thomas Ransom (Nova Scotia Health Authority, Halifax), Oren Steen (LMC Endocrinology Centres [Toronto] Ltd., Toronto), Richard Tytus (Hamilton Medical Research Group, Hamilton), S. Weisnagel (CHU de Quebec-Universite Laval, Quebec), Sean Wharton (The Wharton Medical Clinic, Hamilton)

Hungary: Péter Faludi (Dr. Vass László Egészségügyi Intézmény Diab. Szakrendelés, Budapest), Iván Ory (MH Egeszsegugyi Kozpont, Budapest), Gizella Petro (Kenezy Korhaz, Debrecen), Katalin Revesz (Selye János Kórház és Rendelointézet, Komarom), Zsolt Sudár (Tolna megyei Balassa János Kórház, Szekszárd), Tímea Tanczer (MED-TIMA Kft., Budapest)

Italy: Silvio Buscemi (Policlinico University Hospital, Palermo), Cristina Ciuoli (Azienda Ospedaliera Universitaria Senese, Siena), Geltrude Mingrone (Fondazione Policlinico Universitario Agostino Gemelli IRCCS, Rome), Uberto Pagotto (A.O. Universitaria S.ORSOLA-MALPIGHI – U.O. Endocrinologia Bologna), Ferruccio Santini (Azienda Ospedaliero Universitaria Pisana Ospedale Cisanello, Pisa)

Spain: Juan José Gorgojo Martínez (Hospital Fundación Alcorcón, Alcorcón), Esteban Jódar (Hospital Universitario QuironSalud Madrid, Pozuelo de Alarcon), Pedro Mezquita Raya (Hospital Univer.Torrecárdenas, Almeria), Margarita Rivas Fernández (Hospital Quirónsalud Infanta Luisa, Sevilla), Francisco Javier Salvador Rodríguez (Clinica Universitaria de Navarra, Pamplona), Nuria Vilarrasa (Hospital Universitari de Bellvitge, Hospitalet de Llobregat)

United States: Thomas Blevins (Texas Diabetes & Endocrinology, P.A., Austin), Kenneth Cohen (New West Physicians, Inc., Golden), Matthew Davis (Rochester Clinical Research, Inc., Rochester), H. Jackson Downey (Westside Center For Clinical Research, Jacksonville), Juan Frias (National Research Institute, Los Angeles), W. Timothy Garvey (University of Alabama Birmingham, Birmingham), Gregg Gerety (Albany Medical College, Albany), David Morin (Holston Medical Group, Kingsport), John Nardandrea, Jr (Renstar Medical Research, Ocala), Kerem Ozer (Texas Diabetes & Endocrinology, P.A., Austin), John Pullman (Mercury Street Medical Group, PLLC, Butte), Domenica Rubino (Washington Center for Weight Management and Research, Inc., Arlington), Timothy Smith (StudyMetrix Research LLC, St. Peters), Joseph Soufer (Chase Medical Research LLC, Waterbury), Holly Wyatt (University of Colorado, Aurora)

Supplementary tables

Supplementary Table 1 │ Co-primary, confirmatory secondary, and selected supportive secondary and exploratory trial endpoints (trial product estimand).*

	Semaglutide (n=152)	Placebo (n=152)	Treatment comparison (95% CI)†
**Co-primary endpoints**			
Body weight change from baseline to week 104, %	–16.7% (0.9)	–0.6% (0.9)	ETD –16.0 (–18.6 to –13.5)
≥5% weight loss at week 104	110/132 (83.3%)	38/109 (34.9%)	OR 18.1 (10.0 to 32.5)
**Confirmatory secondary endpoints**			
≥10% weight loss at week 104	89/132 (67.4%)	14/109 (12.8%)	OR 17.6 (9.4 to 32.9)
≥15% weight loss at week 104	75/132 (56.8%)	7/109 (6.4%)	OR 23.6 (10.4 to 53.8)
Waist circumference – change from baseline to week 104, cm	–15.8 (0.9)	–3.7 (1.0)	ETD –12.1 (–14.7 to –9.4)
Systolic blood pressure – change from baseline to week 104, mm Hg	–6.1 (1.2)	–0.1 (1.2)	ETD –6.1 (–9.4 to –2.7)
**Supportive secondary endpoints**			
≥20% weight loss at week 104	52/132 (39.4%)	3/109 (2.8%)	OR 26.7 (8.1 to 87.7)
Body weight			
Change from baseline to week 104, kg	–17.6 (1.0)	–0.8 (1.0)	ETD –16.8 (–19.7 to –13.9)
Change from baseline to week 52, %	–16.6% (0.7)	–2.3% (0.7)	ETD –14.3% (–16.1% to –12.4%)
Body-mass index – change from baseline to week 104, kg/m^2	–6.5 (0.4)	–0.3 (0.4)	ETD –6.2 (–7.3 to –5.1)
HbA_{1c} – change from baseline to week 104, %	–0.5 (0.02)	–0.1 (0.02)	ETD –0.4 (–0.5 to –0.3)

Fasting plasma glucose – change from baseline to week 104, mmol/L	–0.5 (0.04)	0.1 (0.05)	ETD –0.6 (–0.7 to –0.5)
Diastolic blood pressure – change from baseline to week 104, mm Hg	–4.4 (0.8)	–0.7 (0.8)	ETD –3.7 (–5.8 to –1.5)
Fasting serum insulin – change from baseline to week 104, %‡	–36.0%	–8.7%	Estimated relative percentage difference –30.0% (–39.5% to –18.9%)
Lipids – change from baseline to week 104, %‡			
Total cholesterol	–4.6%	2.4%	Estimated relative percentage difference –6.8% (–9.9% to –3.5%)
HDL cholesterol	10.3%	7.4%	Estimated relative percentage difference 2.7% (–1.8% to 7.3%)
LDL cholesterol	–8.0%	0.5%	Estimated relative percentage difference –8.4% (–13.1% to –3.4%)
VLDL cholesterol	–22.4%	1.0%	Estimated relative percentage difference –23.2% (–29.7% to –16.2%)
Free fatty acids	–4.2%	6.0%	Estimated relative percentage difference –9.6% (–21.2% to 3.7%)
Triglycerides	–22.6%	2.0%	Estimated relative percentage difference –24.1% (–30.6% to –16.9%)
C-reactive protein – change from baseline to week 104, %‡	–59.5%	–8.0%	Estimated relative percentage difference –56.0% (–66.0% to –43.1%)

Data are mean (standard error) or observed n/N (%) unless stated otherwise. Participants in the full analysis set are included in the treatment comparisons. *The trial product estimand assesses treatment effect if trial product was taken as intended (i.e., if all participants adhered to treatment and did not receive rescue intervention); see Table 2 for corresponding data for the treatment policy estimand. Continuous endpoint analyses were conducted using a mixed model for repeated measures (MMRM) with randomized treatment as a factor and baseline endpoint value as a covariate. Analyses of categorical endpoints were conducted with the use of logistic regression, with categorization for missing data based on values predicted from the MMRM. Analyses of endpoints for the trial product estimand were not adjusted for multiplicity. †The difference is the estimated treatment difference between the groups except in the case of fasting serum insulin, lipid, and C-reactive protein levels, for which the comparison is the estimated relative percentage difference between groups. ‡These

parameters were initially analyzed on a log scale as estimated ratio to baseline (within treatment groups) and estimated treatment ratios (between treatment groups). For interpretation, these data are expressed as relative percentage change and estimated relative percentage difference between groups, respectively, and were calculated with the following formula: (estimated ratio – 1) × 100. CI, confidence interval; HDL, high-density lipoprotein; LDL, low-density lipoprotein; SEM, standard error of the mean; VLDL, very-low-density lipoprotein.

Supplementary Table 2 │ Malignant neoplasms by system organ class and preferred term (in-trial).*

	Semaglutide (n=152)			Placebo (n=152)		
	**Participants**	**Events**	**Events per 100 patient-years**	**Participants**	**Events**	**Events per 100 patient-years**
Neoplasms benign, malignant, and unspecified (including cysts and polyps)	2 (1.3%)	2	0.6	4 (2.6%)	4	1.3
Basal cell carcinoma	1 (0.7%)	1	0.3	0 (0.0%)		
Bowen's disease	1 (0.7%)	1	0.3	0 (0.0%)		
Invasive ductal breast carcinoma	0 (0.0%)			2 (1.3%)	2	0.7
Lung adenocarcinoma	0 (0.0%)			1 (0.7%)	1	0.3
Small cell lung cancer metastatic	0 (0.0%)			1 (0.7%)	1	0.3

*Events observed during the in-trial period (the time from random assignment to last contact with a trial site, regardless of treatment discontinuation or rescue intervention).

Supplementary Table 3 │ Supportive secondary safety endpoints (on-treatment).*

	Semaglutide 2.4 mg		Placebo	
	N	Mean	N	Mean
Pulse – bpm				
Baseline	152	73±11	152	72±9
Week 104	130	76±9	106	71±10
Change from baseline to week 104†	111	3.3	96	–0.8
Estimated treatment difference (semaglutide *vs* placebo) [95% CI]†	4.1 (2.0 to 6.2)			
Amylase – U/L				
Baseline	152	50 (39.7)	152	52 (33.9)
Week 104	130	57 (42.0)	106	52 (33.3)
Ratio to baseline at week 104	130	1.13 (20.7)	106	1.02 (15.1)
Lipase – U/L				
Baseline	152	22 (54.4)	152	23 (51.3)
Week 104	130	33 (64.6)	106	23 (52.0)
Ratio to baseline at week 104	130	1.47 (52.3)	106	1.00 (34.4)
Calcitonin – ng/L				
Baseline	152	1.3 (75.8)	152	1.3 (82.1)
Week 104	124	1.3 (69.6)	102	1.4 (83.1)
Ratio to baseline at week 104	124	0.99 (21.5)	102	0.97 (41.0)

Data are descriptive statistics presented as arithmetic mean ± standard deviation or geometric mean (coefficient of variation), unless indicated otherwise. *During treatment with trial product (any dose of trial medication administered within the previous 2 weeks (i.e., any period of temporary treatment interruption with trial product was excluded)). †Trial product estimand data (assesses treatment effect if trial product was taken as intended (i.e., if all participants adhered to treatment and did not receive rescue intervention)) analyzed using a mixed model for repeated measurements. CI, confidence interval.

Supplementary Table 4 │ Statistical analysis methodology: analysis and imputation methods to address the treatment policy and trial product estimands for the primary and confirmatory secondary endpoints in the statistical testing hierarchy.

Objective	Endpoint	Test order	Endpoint type	Estimand	Analysis set	Statistical model	Imputation approach	Sensitivity analyses
**Primary endpoints**								
Primary	% weight change	1	Continuous	Treatment policy*	FAS	ANCOVA	RD-MI	J2R-MI<br>S1-SI<br>S2-SI<br>TP-MI<br>MMRM
				Trial product†	FAS	MMRM		
Primary	5% responders	2	Binary	Treatment policy*	FAS	LR	RD-MI	J2R-MI<br>S1-SI<br>S2-SI<br>TP-MI<br>MMRM<br>Non-responder
				Trial product†	FAS	LR	MMRM	
**Confirmatory secondary endpoints**								
Primary	10% responders	3	Binary	Treatment policy*	FAS	LR	RD-MI	Non-responder
				Trial product†	FAS	LR	MMRM	
Primary	15% responders	4	Binary	Treatment policy*	FAS	LR	RD-MI	Non-responder

				Trial product†	FAS	LR	MMRM	
Primary	Waist circumference change (cm)	5	Continuous	Treatment policy*	FAS	ANCOVA	RD-MI	J2R-MI
				Trial product†	FAS	MMRM		
Secondary	Systolic blood pressure change (mm Hg)	6	Continuous	Treatment policy*	FAS	ANCOVA	RD-MI	J2R-MI
				Trial product†	FAS	MMRM		

*Designated as the primary estimand. †Designated as the secondary estimand. Test order refers to the order of the endpoint in the statistical test hierarchy. All analyses were performed using the full analysis set. All statistical tests were two-sided. ANCOVA, analysis of covariance; FAS, full analysis set; J2R-MI, jump to reference multiple imputation; LR, logistic regression; MMRM, mixed model for repeated measurements; RD-MI, multiple imputation using retrieved subjects; S1-SI and S2-SI, single imputation as done by Sacks; TP-MI, tipping point multiple imputation.
