# Supplementary material for: Two-year effects of semaglutide in adults with overweight or obesity: the STEP 5 trial
Source: Nat Med. 2022 Oct 10;28(10):2083–91. doi: 10.1038/s41591-022-02026-4 (PMC9556320; doi:10.1038/s41591-022-02026-4)
Supplement: Supplementary file 2 — Reporting Summary [file 41591_2022_2026_MOESM2_ESM.pdf]

nature portfolio

Corresponding author(s): W. Timothy Garvey

Last updated by author(s): Aug 23, 2022

Reporting Summary

Nature Portfolio wishes to improve the reproducibility of the work that we publish. This form provides structure for consistency and transparency in reporting. For further information on Nature Portfolio policies, see our Editorial Policies and the Editorial Policy Checklist.

Statistics

For all statistical analyses, confirm that the following items are present in the figure legend, table legend, main text, or Methods section.

n/a	Confirmed	
☐	☒	The exact sample size (*n*) for each experimental group/condition, given as a discrete number and unit of measurement
☐	☒	A statement on whether measurements were taken from distinct samples or whether the same sample was measured repeatedly
☐	☒	The statistical test(s) used AND whether they are one- or two-sided<br>*Only common tests should be described solely by name; describe more complex techniques in the Methods section.*
☐	☒	A description of all covariates tested
☐	☒	A description of any assumptions or corrections, such as tests of normality and adjustment for multiple comparisons
☐	☒	A full description of the statistical parameters including central tendency (e.g. means) or other basic estimates (e.g. regression coefficient) AND variation (e.g. standard deviation) or associated estimates of uncertainty (e.g. confidence intervals)
☐	☒	For null hypothesis testing, the test statistic (e.g. *F*, *t*, *r*) with confidence intervals, effect sizes, degrees of freedom and *P* value noted<br>*Give P values as exact values whenever suitable.*
☒	☐	For Bayesian analysis, information on the choice of priors and Markov chain Monte Carlo settings
☐	☒	For hierarchical and complex designs, identification of the appropriate level for tests and full reporting of outcomes
☒	☐	Estimates of effect sizes (e.g. Cohen's *d*, Pearson's *r*), indicating how they were calculated

Our web collection on statistics for biologists contains articles on many of the points above.

Software and code

Policy information about availability of computer code

Data collection	No software was used
Data analysis	Analyses were done using SAS version 9.4

For manuscripts utilizing custom algorithms or software that are central to the research but not yet described in published literature, software must be made available to editors and reviewers. We strongly encourage code deposition in a community repository (e.g. GitHub). See the Nature Portfolio guidelines for submitting code & software for further information.

Data

Policy information about availability of data

All manuscripts must include a data availability statement. This statement should provide the following information, where applicable:

- Accession codes, unique identifiers, or web links for publicly available datasets
- A description of any restrictions on data availability
- For clinical datasets or third party data, please ensure that the statement adheres to our policy

Data will be shared with bona fide researchers submitting a research proposal approved by the independent review board. The research proposal must outline: the scientific rationale and relevance of the proposed research; a short lay summary intended for public disclosure; research methodology and data; statistical analysis plan and publication plan. Data must not be used for commercial purposes. Data will be made available after research completion, and approval of the product and

product use in the European Union and the USA. Individual participant data will be shared in data sets in a de-identified/anonymized format. Access request proposals can be found at novonordisk-trials.com.

Human research participants

Policy information about studies involving human research participants and Sex and Gender in Research.

Reporting on sex and gender	The research findings do not apply to only one sex or gender. Information on the sex of participants was collected by investigators by selecting from 'male' or 'female' on a case report form. Investigators were not provided with any other guidance or instruction on the approach to determination of sex. Data on gender were not collected. Sex and/or gender were not considered within the study design with the exception that sex was included as a factor within the linear regression model for multiple imputation that formed part of the treatment policy estimand analyses, as noted in the Statistical analysis section of the Methods. Source data contain disaggregated sex data. The overall number of male and female patients is reported in the 'Population characteristics' section of the Reporting Summary, and in Table 1 of the manuscript. An analysis of trial endpoints by sex was not prespecified and therefore is not reported. An exploratory post hoc analysis of change from baseline in body weight at week 104 by sex has been conducted by the sponsor for the purposes of the European Medicines Agency submission for semaglutide 2.4 mg, but is not reported in the present manuscript give its exploratory, post hoc nature. Furthermore, the relatively low number of male participants per treatment group in the trial was insufficient to enable meaningful conclusions to be drawn from this post hoc analysis
Population characteristics	Please see Table 1 in the manuscript. Of the 304 randomized patients, 236 (77.6%) were female and 68 (22.4%) were male. Most participants were white (283 [93.1%] of 304), with a mean age of 47.3 years. Mean body weight was 106.0 kg and mean BMI was 38.5 kg/m2.
Recruitment	This study was conducted at 41 sites across five countries (Canada, Italy, Hungary, Spain, and the USA), as described in a previous publication (Kushner RF, Calanna S, Davies M, et al. Semaglutide 2.4 mg for the treatment of obesity: key elements of the STEP trials 1 to 5. Obesity (Silver Spring) 2020; 28: 1050–61) and listed in the Supplementary Information. In total, 347 participants were screened, of whom 304 were randomized to active treatment or placebo.
Ethics oversight	The trial was conducted in accordance with the Declaration of Helsinki and Good Clinical Practice guidelines. The protocol was approved by independent ethics committees or institutional review boards at each study site (a redacted protocol is provided separately).

Note that full information on the approval of the study protocol must also be provided in the manuscript.

Field-specific reporting

Please select the one below that is the best fit for your research. If you are not sure, read the appropriate sections before making your selection.

☒ Life sciences ☐ Behavioural & social sciences ☐ Ecological, evolutionary & environmental sciences

For a reference copy of the document with all sections, see nature.com/documents/nr-reporting-summary-flat.pdf

Life sciences study design

All studies must disclose on these points even when the disclosure is negative.

Sample size	A sample size of 300 participants provided an effective power of at least 96% for the two co primary endpoints, and at least 43% for all confirmatory secondary endpoints, which were tested in a pre defined hierarchical order (Supplementary Table 4).
Data exclusions	No data were excluded from the analyses
Replication	The study was a prospective clinical trial and thus no replication was foreseen within the scope of the study. Replication of results requires further clinical trials.
Randomization	Randomization (1:1) to semaglutide 2.4 mg or placebo was done centrally by the clinical research organization (Parexel) in a double-blind manner using an interactive web-based response system (IWRS) with a fixed-size blocking schema, without stratification. The IWRS generated the randomization list and assigned patients to the next available treatment according to the randomization schedule. The IWRS allocated dispensing unit numbers for each patient, with the trial product dispensed by the site investigator or study coordinator at the trial site visits.
Blinding	The active product and corresponding placebo product were visually identical to maintain masking of participants and site staff. The people analyzing the data were blinded to treatment/group assignment until breaking the blinding at database lock.

Reporting for specific materials, systems and methods

We require information from authors about some types of materials, experimental systems and methods used in many studies. Here, indicate whether each material, system or method listed is relevant to your study. If you are not sure if a list item applies to your research, read the appropriate section before selecting a response.

Materials & experimental systems

n/a	Involved in the study
☒	☐ Antibodies
☒	☐ Eukaryotic cell lines
☒	☐ Palaeontology and archaeology
☒	☐ Animals and other organisms
☐	☒ Clinical data
☒	☐ Dual use research of concern

Methods

n/a	Involved in the study
☒	☐ ChIP-seq
☒	☐ Flow cytometry
☒	☐ MRI-based neuroimaging

Clinical data

Policy information about clinical studies

All manuscripts should comply with the ICMJE guidelines for publication of clinical research and a completed CONSORT checklist must be included with all submissions.

Clinical trial registration

ClinicalTrials.gov identifier: NCT03693430

Study protocol

A redacted protocol is provided as a separate document.

Data collection

This study was conducted at 41 sites across five countries (Canada, Italy, Hungary, Spain, and the USA) from October 5, 2018 to February 1, 2019, as described in a previous publication (Kushner RF, Calanna S, Davies M, et al. Semaglutide 2.4 mg for the treatment of obesity: key elements of the STEP trials 1 to 5. Obesity (Silver Spring) 2020; 28: 1050–617) and listed in the Supplementary Information.

Outcomes

Co-primary endpoints were percentage change in body weight from baseline to week 104 and achievement of weight loss of at least 5% of baseline weight at week 104. These were tested first in the statistical testing hierarchy, followed by the confirmatory secondary endpoints, which were tested in the following order: achievement of weight loss of at least 10% or 15% at week 104; and change from baseline to week 104 in waist circumference and systolic blood pressure.

Supportive secondary endpoints were not included in the statistical testing hierarchy and were: achievement of weight loss of ≥20% at week 104; change from baseline to week 104 in body weight (in kg), BMI, HbA1c, fasting plasma glucose, fasting serum insulin, diastolic blood pressure, lipids (total cholesterol, high-density lipoprotein cholesterol, low-density lipoprotein cholesterol, very-low-density lipoprotein cholesterol, free fatty acids and triglycerides), and C reactive protein; change from baseline to week 52 in body weight (percentage change and kg change), BMI, and waist circumference; and achievement of weight loss of ≥5%, ≥10%, ≥15% and ≥20% at week 52.

Exploratory endpoints reported herein include change from baseline to week 104 in glycemic category, antihypertensive medication use, and lipid-lowering medication use. Glycemic category (normoglycemia, prediabetes or type 2 diabetes) was determined by investigators on the basis of available information (e.g., medical records, concomitant medication, and blood glucose variables) and in accordance with American Diabetes Association criteria, which for prediabetes includes fasting plasma glucose levels of 100 mg/dL (5.6 mmol/L) to 125 mg/dL (6.9 mmol/L) or HbA1c levels of 5.7–6.4% (39–47 mmol/L), and for type 2 diabetes includes fasting plasma glucose levels of ≥126 mg/dL (7.0 mmol/L) or HbA1c levels ≥6.5% (48 mmol/L) (American Diabetes Association. 2. Classification and diagnosis of diabetes. Diabetes Care 2017; 40(Suppl 1): S11–24). The allowance for investigators to use all available information (e.g. concomitant medication) to assess glycemic category was primarily included to account for scenarios in which glucose-lowering medications were initiated during the trial that would confound glycemic category assessment if based purely on fasting plasma glucose or HbA1c levels (for example, if a patient developed diabetes during the study and received a glucose-lowering drug that resulted in their glucose level being below the American Diabetes Association threshold for type 2 diabetes diagnosis). Additional exploratory endpoints for which data are not reported were: permanent discontinuation of trial product between baseline and week 104; time to permanent discontinuation of trial product; and Control of Eating Questionnaire scores from the four domains and 19 individual items (applicable for US and Canada only).

Safety endpoints included: the number of treatment-emergent adverse events and serious adverse events, assessed between baseline and week 111; and change from baseline to week 104 in pulse, amylase, lipase and calcitonin. An independent external event adjudication committee reviewed cardiovascular events, acute pancreatitis, and deaths.
