# Supplementary material for: Two-year effects of semaglutide in adults with overweight or obesity: the STEP 5 trial
Source: Nat Med. 2022 Oct 10;28(10):2083–91. doi: 10.1038/s41591-022-02026-4 (PMC9556320; doi:10.1038/s41591-022-02026-4)
Supplement: Supplementary file 3 — Study protocol. [file 41591_2022_2026_MOESM3_ESM.pdf]

16.1.1 Protocol and protocol amendments

List of contents

Protocol .... Link

Attachments I and II .... Link

Protocol

Protocol title:

Two-year effect and safety of semaglutide 2.4 mg once-weekly in subjects with overweight or obesity

Substance name: semaglutide

Universal Trial Number: U1111-1202-1740

EudraCT Number: 2017-003726-32

Trial phase: 3b

In the following, Novo Nordisk A/S and its affiliates will be stated as "Novo Nordisk".

This ~~confidential~~ document is the property of Novo Nordisk. ~~No unpublished information contained herein may be disclosed without prior written approval from Novo Nordisk. Access to this document must be restricted to relevant parties.~~

Protocol amendment summary of changes table

DOCUMENT HISTORY		
Document	Date	Version
Updated protocol including amendment 1	29 June 2018	3.0
Original protocol	14 May 2018	2.0

Protocol amendment no. 1 (29 June 2018 version 2.0)

This amendment is considered to be non-substantial based on the criteria set forth in Article 10(a) of Directive 2001/20/EC of the European Parliament and the Council of the European Union[1] because it neither significantly impacts the safety nor physical/mental integrity of subjects nor the scientific value of the trial.

Overall rationale for amendment no. 1:

This protocol amendment is prepared to include the Control of Eating Questionnaire (CoEQ) at the following time points: baseline (week 0), week 20, week 52 and week 104. The questionnaire will be implemented in US and Canada only.

Section # and name	Description of change	Rationale
Section 10.3.3 Exploratory endpoints	Adding that the rating for the CoEQ will be summarised using descriptive statistics	As this is an exploratory endpoint
Section 9 Trial assessments and procedures and 9.1.3	Adding patient reported outcome questionnaires and description of how the subjects should complete the questionnaire	To explain how the questionnaire should be completed
Section 4.2.3 Exploratory endpoints	Adding Control of Eating Questionnaire (CoEQ): Scores from the 4 domains and 19 individual items as an explorative endpoint	GLP-1 is a physiological regulator of appetite. The CoEQ assesses the intensity and type of food cravings, as well as subjective sensations of appetite and mood. Including the CoEQ will provide valuable information on the long-term effect of semaglutide 2.4 mg on appetite and food craving
Section 4.1 Primary, secondary and exploratory objective(s)	Adding control of eating as an explorative objective	Same as above

Table of Contents

Page

Protocol amendment summary of changes table ... **2**

Table of Contents ... **3**

1 Synopsis ... **6**

2 Flowchart ... **9**

3 Introduction ... **13**
- 3.1 Trial rationale ... 13
- 3.2 Background ... 14
  - 3.2.1 Semaglutide ... 14
  - 3.2.2 Trial population ... 14
- 3.3 Benefit-risk assessment ... 15
  - 3.3.1 Benefits ... 15
  - 3.3.2 Risks and precautions ... 15
  - 3.3.3 Conclusion on risk-benefit profile ... 16

4 Objectives and endpoints ... **17**
- 4.1 Primary, secondary and exploratory objective(s) ... 17
- 4.2 Primary, secondary and exploratory endpoint(s) ... 18
  - 4.2.1 Primary endpoint ... 18
  - 4.2.2 Secondary endpoints ... 18
    - 4.2.2.1 Confirmatory secondary endpoints ... 18
    - 4.2.2.2 Supportive secondary endpoints ... 18
  - 4.2.3 Exploratory endpoints ... 19

5 Trial design ... **20**
- 5.1 Overall design ... 20
- 5.2 Subject and trial completion ... 21
- 5.3 End of trial definition ... 21
- 5.4 Scientific rationale for trial design ... 21
- 5.5 Justification for dose ... 21

6 Trial population ... **22**
- 6.1 Inclusion criteria ... 22
- 6.2 Exclusion criteria ... 22
- 6.3 Randomisation criteria ... 24
- 6.4 Lifestyle restrictions ... 24
  - 6.4.1 Meals and dietary restrictions ... 24
  - 6.4.2 Caffeine and tobacco ... 24
- 6.5 Screen failures ... 24

7 Treatments ... **25**
- 7.1 Treatments administered ... 25
  - 7.1.1 Medical devices ... 27
  - 7.1.2 Diet and physical activity counselling ... 27
- 7.2 Dose modification ... 28
- 7.3 Method of treatment assignment ... 28

7.4 Blinding 28
7.5 Preparation/Handling/Storage/Accountability 29
7.6 Treatment compliance 30
7.7 Concomitant medication 30
7.7.1 Rescue medication 30
7.8 Treatment after the end of the trial 30

8 Discontinuation/Withdrawal criteria 31
8.1 Discontinuation of trial treatment 31
8.1.1 Temporary discontinuation of trial treatment 32
8.2 Withdrawal from the trial 32
8.2.1 Replacement of subjects 32
8.3 Lost to follow-up 33

9 Trial assessments and procedures 33
9.1 Efficacy assessments 34
9.1.1 Body measurements 34
9.1.2 Clinical efficacy laboratory assessments 35
9.1.3 Clinical outcome assessment (applicable for US and Canada only) 35
9.2 Adverse events 35
9.2.1 Time period and frequency for collecting AE and SAE information 35
9.2.1.1 Events for adjudication 37
9.2.2 Method of detecting AEs and SAEs 38
9.2.3 Follow-up on AEs and SAEs 38
9.2.4 Regulatory reporting requirements for SAEs 38
9.2.5 Hypoglycaemic, cardiovascular and death events 38
9.2.6 Disease-related events and/or disease-related outcomes not qualifying as an AE or SAE 38
9.2.7 Pregnancies and associated adverse events 38
9.2.8 Medical device incidents (including malfunctions) 39
9.2.9 Technical complaints 39
9.3 Treatment of overdose 40
9.4 Safety assessments 40
9.4.1 Mental health assessment instruments 41
9.4.2 Physical examinations 42
9.4.3 Vital signs 42
9.4.4 Electrocardiograms 42
9.4.5 Clinical safety laboratory assessments 42
9.5 Pharmacokinetics 43
9.6 Pharmacodynamics 43
9.7 Genetics 43
9.8 Biomarkers 43
9.9 Severe hypersensitivity 43

10 Statistical considerations 44
10.1 Sample size determination 44
10.2 Definition of analysis sets 46
10.3 Statistical analyses 47
10.3.1 Primary endpoint 47
10.3.2 Secondary endpoints 52

10.3.2.1 Confirmatory secondary endpoints 52
10.3.2.2 Supportive secondary endpoints 53
10.3.3 Exploratory endpoint 55
10.3.4 Explorative statistical analysis for pharmacogenetics and biomarkers 56
10.3.5 Other analyses 56
10.4 Pharmacokinetic and/or pharmacodynamic modelling 56

11 Appendices 57

Appendix 1 Abbreviations and Trademarks 58

Appendix 2 Clinical laboratory tests 61

Appendix 3 Trial governance considerations 64

Appendix 4 Adverse events: definitions and procedures for recording, evaluation, follow-up, and reporting 74

Appendix 5 Contraceptive guidance and collection of pregnancy information 80

Appendix 6 Technical complaints: Definition and procedures for recording, evaluation, follow-up and reporting 83

Appendix 7 Monitoring of calcitonin 85

Appendix 8 Country-specific requirements 88

12 References 89

Attachment I Global list of key staff and relevant departments and suppliers

Attachment II Country list of key staff and relevant departments

1 Synopsis

Rationale:

Lifestyle intervention in the form of diet and exercise is first line treatment for obesity, but most people with obesity struggle to achieve and maintain their weight loss[2-11]. Surgical treatments offer an effective alternative for some people with severe obesity, but surgery carries a risk in connection with the procedure and is not without complications. Furthermore, surgery requires close follow-up of the individual which can be cumbersome and costly[2-7, 12, 13]. Pharmacotherapy may therefore serve as a valuable adjunct to lifestyle intervention for individuals with obesity in order to achieve and sustain a clinically relevant weight loss, to improve comorbid conditions and to facilitate a healthier lifestyle. Few anti-obesity medications are currently available and there is a need for more safe and effective therapeutic options for treatment of obesity, especially treatments that also target weight maintenance, prevention and treatment of comorbidities[2-6, 14, 15].

The present trial is designed to compare the two-year effect and safety of subcutaneous (s.c.) semaglutide 2.4 mg once weekly versus semaglutide placebo as an adjunct to a reduced-calorie diet and increased physical activity in subjects with overweight or obesity. A two-year period is considered an appropriate period of time for evaluation of long-term treatment with s.c. semaglutide 2.4 mg once weekly.

Objectives and endpoints

Primary objective

To compare the two-year effect of semaglutide s.c. 2.4 mg once weekly versus semaglutide placebo as an adjunct to a reduced-calorie diet and increased physical activity in subjects with overweight or obesity on body weight.

Secondary objective

To compare the two-year effect of semaglutide s.c. 2.4 mg once weekly versus semaglutide placebo as an adjunct to a reduced-calorie diet and increased physical activity in subjects with overweight or obesity on:

- Cardiovascular risk factors
- Glucose metabolism

To compare the one-year effect of semaglutide s.c. 2.4 mg once weekly versus semaglutide placebo as an adjunct to a reduced-calorie diet and increased physical activity in subjects with overweight or obesity on body weight

To compare the two-year safety and tolerability of semaglutide s.c. 2.4 mg once weekly versus semaglutide placebo as an adjunct to a reduced-calorie diet and increased physical activity in subjects with overweight or obesity

Primary estimand

The estimand will quantify the average treatment effect of semaglutide relative to semaglutide placebo after 104 weeks, as an adjunct to a reduced-calorie diet and increased physical activity, in all randomised subjects regardless of adherence to treatment and regardless of starting rescue interventions (weight management drugs or bariatric surgery) ("effectiveness"/"treatment policy" estimand). The estimand will cover all effect-related objectives.

The following expansion of the primary estimand will cover objectives related to weight. The estimand will quantify the average treatment effect of semaglutide relative to semaglutide placebo after 52 weeks, as an adjunct to a reduced-calorie diet and increased physical activity, in all randomised subjects regardless of adherence to treatment and regardless of starting rescue interventions.

Primary endpoint

The primary endpoints addressing the primary objective:

- Change from baseline (week 0) to week 104 in body weight (%)
- Subjects who after 104 weeks achieve (yes/no):
    - Body weight reduction ≥ 5% from baseline (week 0)

Confirmatory secondary endpoint

- Subjects who after 104 weeks achieve (yes/no):
    - Body weight reduction ≥ 10% from baseline (week 0)
    - Body weight reduction ≥ 15% from baseline (week 0)

- Change from baseline (week 0) to week 104 in:
    - Waist circumference (cm)
    - Systolic blood pressure (mmHg)

Overall design:

This is a 104-week, randomised, double-blind, placebo-controlled, two-armed, parallel group, multi-centre, multinational clinical trial comparing semaglutide s.c. 2.4 mg once weekly with semaglutide placebo in subjects with overweight or obesity.

Eligible subjects fulfilling all randomisation criteria at visit 2 will be randomised in a 1:1 manner to receive either semaglutide s.c. 2.4 mg once weekly or semaglutide placebo as an adjunct to a reduced-calorie diet and increased physical activity.

Key inclusion criteria

- Male or female, age ≥ 18 years at the time of signing informed consent

- Body mass index (BMI) ≥ 30 kg/m^2 or ≥ 27 kg/m^2 with the presence of at least one of the following weight-related comorbidities (treated or untreated): hypertension, dyslipidaemia, obstructive sleep apnoea or cardiovascular disease
- History of at least one self-reported unsuccessful dietary effort to lose body weight

Key exclusion criteria

- HbA_{1c} ≥ 48 mmol/mol (6.5%) as measured by the central laboratory at screening
- A self-reported change in body weight > 5 kg (11 lbs) within 90 days before screening irrespective of medical records

Number of subjects:

Approximately 353 subjects will be screened to achieve 300 subjects randomly assigned to trial product.

Treatment groups and duration:

Eligible subjects fulfilling all randomisation criteria at visit 2 will be randomised in a 1:1 manner to receive either s.c. semaglutide 2.4 mg once weekly or semaglutide placebo as an adjunct to a reduced-calorie diet and increased physical activity.

The total trial duration for the individual subject will be approximately 112 weeks. The trial includes a screening period of approximately 1 week with a visit to assess the subject's eligibility. Subjects will in the first 16 weeks be dose escalated from 0.25 mg once-weekly until target dose with visits/phone contacts every 2nd week during dose escalation. From week 20, visits/phone contacts will take place every 4th week for the remaining maintenance period until end of treatment (week 104). A follow-up visit ('End of trial') for safety assessments is scheduled 7 weeks after end of treatment to account for the exposure and to the long half-life of semaglutide.

The following trial products will be supplied by Novo Nordisk A/S for the duration of the trial:

- Semaglutide B 1.0 mg/mL PDS290 and semaglutide placebo, solution for injection, 3 mL PDS290 pre-filled pen-injector
- Semaglutide B 3.0 mg/mL PDS290 and semaglutide placebo, solution for injection, 3 mL PDS290 pre-filled pen-injector

2 Flowchart

	Screening	Randomisa-tion	Dose escalation period								Maintenance period																						End of treatment	End of trial
Visit (V), Phone(P)	V1	V2	P3	V4	P5	V6	P7	V8	P9	V10	P11	V12	P13	V14	P15	V16	P17	V18	P19	V20	P21	V22	P23	V24	P25	V26	P27	V28	P29	V30	P31	V32	V33	V34
Timing of Visit (Weeks)	-1	0	2	4	6	8	10	12	14	16	18	20	24	28	32	36	40	44	48	52	56	60	64	68	72	76	80	84	88	92	96	100	104	111
Visit Window (Days)	-7 to 0	0	±3	±3	±3	±3	±3	±3	±3	±3	±3	±3	±3	±3	±3	±3	±3	±3	±3	±3	±3	±3	±3	±3	±3	±3	±3	±3	±3	±3	±3	±3	±3	0 to +5
SUBJECT RELATED INFOR-MATION AND ASSESSMENTS																																		
Informed consent and Demography [a] Appendix 3	X																																	
Childbearing potential [b] Appendix 5	X																																	
Inclusion criteria (6.1)	X	X																																
Exclusion criteria (6.2)	X	X																																
Randomisation criteria and randomisation (6.3)		X																																
Medical history/Concomitant illness (9.4)	X																																	
Weight History (9)		X																																
History of Gallbladder Disease (9.4)	X																																	
History of Breast Neoplasm [b] (9.4)	X																																	
History of Colon Neoplasm (9.4)	X																																	
History of Skin Cancer (9.4)	X																																	
History of Psychiatric disorder (9.4)	X																																	
Tobacco use [c]	X																																	
Concomitant medication (7.7)	X	X	X	X	X	X	X	X	X	X	X	X	X	X	X	X	X	X	X	X	X	X	X	X	X	X	X	X	X	X	X	X	X	
Trial product compliance (7.1) (7.6)			X	X	X	X	X	X	X	X	X	X	X	X	X	X	X	X	X	X	X	X	X	X	X	X	X	X	X	X	X	X	X	
Evaluation of lipid-lowering treatment (9)													X								X								X				X	
Evaluation of antihypertensive treatment (9)													X								X								X				X	
Evaluation of glycaemic status (9)		X											X								X								X				X	

	Screening	Randomisation	Dose escalation period								Maintenance period																						End of treatment	End of trial
Visit (V), Phone(P)	V1	V2	P3	V4	P5	V6	P7	V8	P9	V10	P11	V12	P13	V14	P15	V16	P17	V18	P19	V20	P21	V22	P23	V24	P25	V26	P27	V28	P29	V30	P31	V32	V33	V34
Timing of Visit (Weeks)	-1	0	2	4	6	8	10	12	14	16	18	20	24	28	32	36	40	44	48	52	56	60	64	68	72	76	80	84	88	92	96	100	104	111
Visit Window (Days)	-7 to 0	0	±3	±3	±3	±3	±3	±3	±3	±3	±3	±3	±3	±3	±3	±3	±3	±3	±3	±3	±3	±3	±3	±3	±3	±3	±3	±3	±3	±3	±3	±3	±3	0 to +5
EFFICACY																																		
Body measurements (9.1.1)																																		
Height	X																																	
Body weight	X	X		X		X		X		X		X		X		X		X		X		X		X		X		X		X		X	X	X
Waist circumference	X	X		X		X		X		X		X		X		X		X		X		X		X		X		X		X		X	X	
HbA$_{1c}$ (Appendix 2)	X	X										X								X								X					X	
Fasting plasma glucose (Appendix 2)		X										X								X								X					X	
Fasting serum insulin (Appendix 2)		X																															X	
Lipids (Appendix 2)		X										X								X								X					X	
Biomarkers (Appendix 2)		X										X								X								X					X	
Vital signs (9.4.3)																																		
Systolic Blood Pressure	X	X		X		X		X		X		X		X		X		X		X		X		X		X		X		X		X	X	X
Diastolic Blood Pressure	X	X		X		X		X		X		X		X		X		X		X		X		X		X		X		X		X	X	X
Control of Eating Questionnaire (CoEQ)[d] 9.1.3		x										x								x													x	
SAFETY																																		
Physical examination (9.4.2)	X																			X													X	
Pregnancy test [b] (9.4.5, Appendix 5)	X	X		X		X		X		X		X		X		X		X		X		X		X		X		X		X		X	X	X
ECG (9.4.4)		X										X								X								X					X	
Adverse event (9.2, Appendix 4)		X	X	X	X	X	X	X	X	X	X	X	X	X	X	X	X	X	X	X	X	X	X	X	X	X	X	X	X	X	X	X	X	X
Technical complaint (9.2.9, Appendix 4)			X	X	X	X	X	X	X	X	X	X	X	X	X	X	X	X	X	X	X	X	X	X	X	X	X	X	X	X	X	X	X	

	Screening	Randomisation	Dose escalation period								Maintenance period																						End of treatment	End of trial
Breast neoplasms follow-up [b] (9.4)																																	X	X
Visit (V), Phone(P)	V1	V2	P3	V4	P5	V6	P7	V8	P9	V10	P11	V12	P13	V14	P15	V16	P17	V18	P19	V20	P21	V22	P23	V24	P25	V26	P27	V28	P29	V30	P31	V32	V33	V34
Timing of Visit (Weeks)	-1	0	2	4	6	8	10	12	14	16	18	20	24	28	32	36	40	44	48	52	56	60	64	68	72	76	80	84	88	92	96	100	104	111
Visit Window (Days)	-7 to 0	0	±3	±3	±3	±3	±3	±3	±3	±3	±3	±3	±3	±3	±3	±3	±3	±3	±3	±3	±3	±3	±3	±3	±3	±3	±3	±3	±3	±3	±3	±3	±5	0 to +5
Colon neoplasms follow-up (9.4)																																	X	X
Haematology (Appendix 2)	X											X								X								X					X	
Biochemistry (Appendix 2)	X											X								X								X					X	
Vital Signs (9.4.3)																																		
Pulse	X	X		X		X		X		X		X		X		X		X		X		X		X		X		X		X		X	X	X
Patient Health Questionnaire– 9 (PHQ-9) (9.4.1)	X	X						X				X				X				X				X				X					X	
Columbia Suicide Severity Rating Scale (C-SSRS) (9.4.1)	X	X						X				X				X				X				X				X					X	
TRIAL MATERIAL																																		
First date on trial product			X																															
IWRS session	X	X				X				X		X		X		X		X		X		X		X		X		X		X		X	X	
Administration of trial product (7.1, 7.5)																																		
Dispensing visit		X				X				X		X		X		X		X		X		X		X		X		X		X		X		
Drug accountability		X				X				X		X		X		X		X		X		X		X		X		X		X		X	X	
REMINDERS																																		
Criteria for discontinuation (8.1)			X	X	X	X	X	X	X	X	X	X	X	X	X	X	X	X	X	X	X	X	X	X	X	X	X	X	X	X	X	X		
Barriers and motivation interview (9)	X																																	
Diet and physical activity counselling (7.1.2)		X		X		X		X		X		X	X	X	X	X	X	X	X	X	X	X	X	X	X	X	X	X	X	X	X	X	X	
Hand out directions for use (7.1.1)		X																																

	Screening	Randomisa-tion	Dose escalation period								Maintenance period																						End of treatment	End of trial
Training in trial product, pen-handling (7.1.1)		X		X		X		X		X		X		X		X		X		X		X		X		X		X		X		X		
Visit (V), Phone(P)	V1	V2	P3	V4	P5	V6	P7	V8	P9	V10	P11	V12	P13	V14	P15	V16	P17	V18	P19	V20	P21	V22	P23	V24	P25	V26	P27	V28	P29	V30	P31	V32	V33	V34
Timing of visit (Weeks)	-1	0	2	4	6	8	10	12	14	16	18	20	24	28	32	36	40	44	48	52	56	60	64	68	72	76	80	84	88	92	96	100	104	111
Visit Window (Days)	-7 to 0	0	±3	±3	±3	±3	±3	±3	±3	±3	±3	±3	±3	±3	±3	±3	±3	±3	±3	±3	±3	±3	±3	±3	±3	±3	±3	±3	±3	±3	±3	±3	±5	0 to +5
Hand out dose reminder card (7.1)		X		X		X		X		X																								
Hand out and instruct in food diary (9)	X																																	
Hand out ID card	X																																	
Attend visit fasting (6.4.1)		X										X								X								X					X	

[a] Demography consists of date of birth, sex, ethnicity, and race (according to local regulation)
[b] For all female subjects
[c] Smoking is defined as smoking at least one cigarette or equivalent daily
[d] Applicable for US and Canada only

3 Introduction

3.1 Trial rationale

The prevalence of obesity has reached epidemic proportions in most countries around the world and the prevalence is still increasing at an alarming rate[16-22]. The medical and societal impacts are considerable and obesity is one of the most significant public health challenges worldwide[16-22]. Obesity is associated with increased risk of a variety of comorbidities including hyperglycaemia, type 2 diabetes (T2D), hypertension, dyslipidaemia, obstructive sleep apnoea, atherosclerosis, osteoarthritis, urinary incontinence, non-alcoholic steatohepatitis, cardiovascular diseases, certain types of cancer, and risk of early death[23-37]. Moreover, obesity adversely affects physical and mental health and reduces health related quality of life[38, 39]. Obesity is also associated with decreased cardiorespiratory fitness, which also increases the risk of cardiovascular diseases and all-cause mortality[40].

The risk of obesity-related complications and comorbidities increases with increasing BMI, and a weight loss of 5−10% has significant health benefits in terms of slowing progression to T2D[41-44]. Furthermore, a weight loss of 5−10% improves many other obesity related comorbidities as well as physical symptoms and quality of life[45-52]. Finally, studies suggest a beneficial impact of weight loss on cardio vascular risk and mortality in both people with diabetes and obesity[53-55].

Lifestyle intervention in the form of diet and exercise is first line treatment for obesity, but most people with obesity struggle to achieve and maintain their weight loss[2-11]. Surgical treatments offer an effective alternative for some people with severe obesity, but surgery carries a risk in connection with the procedure and is not without complications. Furthermore, surgery requires close follow-up of the individual which can be cumbersome and costly[2-7, 12, 13]. Pharmacotherapy may therefore serve as a valuable adjunct to lifestyle intervention for individuals with obesity in order to achieve and sustain a clinically relevant weight loss, to improve comorbid conditions and to facilitate a healthier lifestyle. Few anti-obesity medications are currently available and there is a need for more safe and effective therapeutic options for treatment of obesity, especially treatments that also target weight maintenance, prevention and treatment of co-morbidities[2-6, 14, 15].

The present trial is designed to compare the two-year effect and safety of subcutaneous (s.c.) semaglutide 2.4 mg once weekly versus semaglutide placebo as an adjunct to a reduced-calorie diet and increased physical activity in subjects with overweight or obesity. A two-year period is considered an appropriate period of time for evaluation of long-term treatment with s.c. semaglutide 2.4 mg once weekly.

3.2 Background

3.2.1 Semaglutide

Semaglutide is the next generation glucagon like peptide -1 (GLP-1) receptor agonist (RA) currently under development by Novo Nordisk for the treatment of weight management (NN9536). Semaglutide has been optimised resulting in a longer half-life of approximately 160 hours, making it suitable for once-weekly dosing[56]. GLP-1 is a physiological regulator of appetite and GLP-1 receptors are present in several areas of the brain involved in appetite regulation[57].

A 52-week phase 2 dose-finding trial within weight management (NN9536-4153) has recently been completed. A total of 957 randomised subjects with obesity (without diabetes) were exposed to semaglutide (n=718), liraglutide 3.0 mg (n=103) or placebo (n=136). In this trial, an overall monotone dose-dependent weight loss was observed across the 5 semaglutide doses tested (0.05 to 0.4 mg once-daily). The estimated weight loss at week 52 was 13.8% at the highest dose tested (0.4 mg once-daily) compared to the weight loss of 2.3% achieved by diet, exercise and placebo alone[58].

Clinical[59-63] and non-clinical data[64] indicate that the body weight-reducing effect of semaglutide is mainly mediated by a reduced energy intake. No unexpected safety findings were identified and the tolerability and safety profile was overall consistent with previous findings in the T2D development programme and the GLP-1 RA class in general.

A comprehensive review of results from the non-clinical and clinical studies of semaglutide can be found in the current edition of the investigator's brochure (IB)[58] and any updates hereof.

3.2.2 Trial population

The trial population will consist of subjects with obesity (BMI $\geq$ 30 kg/m^2) or with overweight (BMI $\geq$ 27 kg/m^2) and weight-related comorbidities. These subjects represent a clinically relevant population for pharmacotherapeutic weight management as they are at significant risk for weight-related morbidities and mortality, and are likely to benefit from weight reduction. Information about weight-related comorbidities, including hypertension, dyslipidaemia, obstructive sleep apnoea or cardiovascular disease, will be collected systematically at screening by the investigators as part of the medical history.

Subjects with T2D are excluded from the trial. Subjects with overweight or obesity and T2D will be addressed in a dedicated trial.

First line treatment in weight management should always be lifestyle modification through a reduced calorie diet and increased physical activity. Thus only subjects who have tried but failed a dietary weight loss intervention will be included in accordance with regulatory guidelines[65, 66].

3.3 Benefit-risk assessment

3.3.1 Benefits

Subjects will be treated with a regimen anticipated to be better than or equal to the weight management they receive at the time of entry into the trial. Results from the phase 2 trial (NN9536-4153) demonstrated that semaglutide once-daily as an adjunct to a reduced-calorie diet and increased physical activity was effective for weight loss in subjects with obesity, while displaying a satisfactory tolerability profile. Overall, a monotone dose-dependent weight loss was observed across all tested doses of semaglutide (0.05 to 0.4 mg once-daily). The weight loss was 11.55 percentage points larger for the 0.4 mg group compared with placebo. Weight loss was accompanied by a consistent improvement in the weight-related comorbidities, indicated by cardiovascular risk factors, lipid profile and glycaemic factors, as well as in clinical outcome assessments.

In addition, it is expected that all subjects will benefit from participation through close contact with the trial site and counselling by a dietician or a similar qualified healthcare professional, all of which will most likely result in intensified weight management.

3.3.2 Risks and precautions

The sections below describe identified and potential risks associated with semaglutide treatment. For classification and further details of the risks, please refer to the current version of the IB or any updates hereof. The identified/potential risks are based on findings in non-clinical studies and clinical trials with semaglutide as well as other GLP-1 RAs. For each of these risks, mitigating actions have been implemented to minimise the risks for subjects enrolled in this trial.

- Gastrointestinal adverse events
  - Consistent with findings with other GLP-1 RAs, the most frequently reported adverse events (AE) in clinical trials with semaglutide were gastrointestinal AEs. A low starting dose and dose escalation steps will be implemented in the trial to mitigate the risk of gastrointestinal AEs.
- Cholelithiasis
  - Events of cholelithiasis were the most frequently reported gallbladder events in the phase 2 weight management trial (NN9536-4153) and were in a few instances co-reported with the event adjudication committee (EAC) confirmed acute pancreatitis. As a precaution, if cholelithiasis is suspected, appropriate clinical follow-up is to be initiated at the investigator’s discretion.
- Acute pancreatitis
  - Acute pancreatitis has been observed with the use of GLP-1 RA drug class. As a precaution, subjects with a history of chronic pancreatitis or recent acute pancreatitis will

not be enrolled in the trial. In addition trial product should be discontinued in case of suspicion of acute pancreatitis in accordance to section 8.1.

- Medullary thyroid cancer (MTC) (based on non-clinical data)
  - Expected proliferative thyroid C-cell changes were seen in the mouse and rat carcinogenicity studies after daily exposure to semaglutide for 2 years. No hyperplasia was observed in monkeys after 52 weeks exposure up to 13-fold above the clinical plasma exposure at 2.4 mg/week. In clinical trial with semaglutide, there have been no clinically relevant changes in calcitonin levels. The C-cell changes in rodents are mediated by the GLP-1 receptor, which is not expressed in the normal human thyroid. Accordingly, the risk of GLP-1 receptor-mediated C-cell changes in humans is considered to be low. However, as a precaution, exclusion and discontinuation criteria related to medical history of multiple endocrine neoplasia type 2 (MEN2) or MTC elevated plasma levels of calcitonin (biomarker for MTC) have been implemented in the trial.
- Pancreatic cancer
  - There is currently no support from non-clinical studies, clinical trials or post-marketing data that GLP-1 RA-based therapies increase the risk of pancreatic cancer, but pancreatic cancer has been classified as a potential class risk of GLP-1 RAs by European Medicines Agency (EMA). As a precaution, subjects with a history of malignant neoplasms within the past 5 years prior to screening will be excluded from the trial.
- Allergic reactions
  - As is the case with all protein-based pharmaceuticals, subjects treated with semaglutide are at risk of developing immunogenic and allergic reactions. As a precaution, subjects with known or suspected hypersensitivity to semaglutide or related products will not be enrolled in this trial.
- Pregnancy and fertility (based on non-clinical data)
  - Studies in animals have shown reproductive toxicity. There are limited data from the use of semaglutide in pregnant women. Therefore, semaglutide should not be used during pregnancy. Exclusion and discontinuation criteria related to pregnancy have been implemented in the trial.

3.3.3 Conclusion on risk-benefit profile

Necessary precautions have been implemented in the design and planned conduct of the trial in order to minimise the risks and inconveniences of participation in the trial. The safety profile for semaglutide generated from the clinical and non-clinical development programme has not revealed any safety issues that would prohibit administration of semaglutide 2.4 mg once-weekly. The results of the phase 2 trial (NN9536-4153) indicate that semaglutide will provide a clinically meaningful weight loss.

In conclusion, the potential risk to the subjects in this trial is considered low and outweighed by the anticipated benefits that semaglutide would provide subjects with overweight or obesity.

More detailed information about the known and expected benefits and risks and reasonably expected AEs of semaglutide s.c. may be found in the IB[58] and any updates hereof.

4 Objectives and endpoints

4.1 Primary, secondary and exploratory objective(s)

Primary objective

To compare the two-year effect of semaglutide s.c. 2.4 mg once weekly versus semaglutide placebo as an adjunct to a reduced-calorie diet and increased physical activity in subjects with overweight or obesity on body weight.

Secondary objectives

To compare the two-year effect of semaglutide s.c. 2.4 mg once weekly versus semaglutide placebo as an adjunct to a reduced-calorie diet and increased physical activity in subjects with overweight or obesity on:

- Cardiovascular risk factors
- Glucose metabolism

To compare the one-year effect of semaglutide s.c. 2.4 mg once weekly versus semaglutide placebo as an adjunct to a reduced-calorie diet and increased physical activity in subjects with overweight or obesity on body weight.

To compare the two-year safety and tolerability of semaglutide s.c. 2.4 mg once weekly versus semaglutide placebo as an adjunct to a reduced-calorie diet and increased physical activity in subjects with overweight or obesity.

Exploratory objectives

To compare the two-year effect of semaglutide s.c. 2.4 mg once weekly versus semaglutide placebo as an adjunct to a reduced-calorie diet and increased physical activity in subjects with overweight or obesity on:

- Glycaemic status
- Use of medication for hypertension and dyslipidaemia.
- Treatment discontinuation
- Control of eating (applicable for US and Canada only)

Primary estimand

The estimand will quantify the average treatment effect of semaglutide relative to semaglutide placebo after 104 weeks, as an adjunct to a reduced-calorie diet and increased physical activity, in all randomised subjects regardless of adherence to treatment and regardless of starting rescue

interventions (weight management drugs or bariatric surgery) ("effectiveness"/"treatment policy" estimand). The estimand will cover all effect-related objectives.

The following expansion of the primary estimand will cover objectives related to weight. The estimand will quantify the average treatment effect of semaglutide relative to semaglutide placebo after 52 weeks, as an adjunct to a reduced-calorie diet and increased physical activity, in all randomised subjects regardless of adherence to treatment and regardless of starting rescue interventions.

Secondary estimand

The estimand will quantify the average treatment effect of semaglutide relative to semaglutide placebo after 104 weeks, as an adjunct to a reduced-calorie diet and increased physical activity, in all randomised subjects had they remained on their randomised treatment for the entire planned duration of the trial and not started any rescue intervention (weight management drugs or bariatric surgery) ("efficacy"/"hypothetical" estimand). The estimand will cover the primary objective.

4.2 Primary, secondary and exploratory endpoint(s)

4.2.1 Primary endpoint

The primary endpoints addressing the primary objective:

- Change from baseline (week 0) to week 104 in body weight (%)
- Subjects who after 104 weeks achieve (yes/no):
  - Body weight reduction ≥ 5% from baseline (week 0)

4.2.2 Secondary endpoints

The confirmatory and supportive secondary endpoints addressing the primary and secondary objectives are listed in section 4.2.2.1 and 4.2.2.2

4.2.2.1 Confirmatory secondary endpoints

- Subjects who after 104 weeks achieve (yes/no):
  - Body weight reduction ≥ 10% from baseline (week 0)
  - Body weight reduction ≥ 15% from baseline (week 0)
- Change from baseline (week 0) to week 104 in:
  - Waist circumference (cm)
  - Systolic blood pressure (mmHg)

4.2.2.2 Supportive secondary endpoints

Effect endpoints:

- Change from baseline (week 0) to week 104 in:
  - Body weight (kg)

- BMI (kg/m^2)
- HbA_{1c} (%, mmol /mol)
- FPG (mg/dL)
- Fasting serum insulin (mIU/L)
- Diastolic blood pressure (mmHg)
- Lipids (mg/dL)
    - Total cholesterol
    - High density lipoprotein (HDL) cholesterol
    - Low density lipoprotein (LDL) cholesterol
    - Very low density lipoprotein (VLDL) cholesterol
    - Free fatty acids (FFA)
    - Triglycerides
- High sensitivity C-Reactive Protein (hsCRP) (mg/L)

- Change from baseline (week 0) to week 52 in:
    - Body weight (%, kg)
    - BMI (kg/m^2)
    - Waist circumference (cm)
- Subjects who after 52 weeks achieve (yes/no):
    - Body weight reduction ≥ 5% from baseline (week 0)
    - Body weight reduction ≥ 10% from baseline (week 0)
    - Body weight reduction ≥ 15% from baseline (week 0)

Safety endpoints:

- Number of treatment emergent adverse events (TEAEs) from baseline (week 0) to week 111
- Number of serious adverse events (SAEs) from baseline (week 0) to week 111
- Change from baseline (week 0) to week 104 in:
    - Pulse (bpm)
    - Amylase (U/L)
    - Lipase (U/L)
    - Calcitonin (ng/L)

4.2.3 Exploratory endpoints

The exploratory endpoints addressing the explorative objectives:

- Change from baseline (week 0) to week 104 in:
    - Glycaemic category (normo-glycaemia, pre-diabetes, T2D)
    - Antihypertensive medication (decrease, no change, increase)
    - Lipid-lowering medication (decrease, no change, increase)

- Subjects who from randomisation to week 104 have permanently discontinued randomised trial product (yes/no)
- Time to permanent discontinuation of randomised trial product (weeks)
- Control of Eating Questionnaire (CoEQ): Scores from the 4 domains and 19 individual items (applicable for US and Canada only)

5 Trial design

5.1 Overall design

This is a 104-week, randomised, double-blind, placebo-controlled, two-armed, parallel group, multi-centre, multinational clinical trial comparing semaglutide 2.4 mg once weekly with semaglutide placebo in subjects with overweight or obesity.

The trial includes a screening visit to assess the subject's eligibility followed by visits/phone contacts every 2nd week during dose escalation. From week 20, visits/phone contacts will take place every 4th week for the remaining maintenance period until end of treatment (week 104). A follow-up visit ('End of trial') for safety assessments is scheduled 7 weeks after end of treatment to account for the exposure to the long half-life of semaglutide.

The trial design is outlined in Figure 5-1

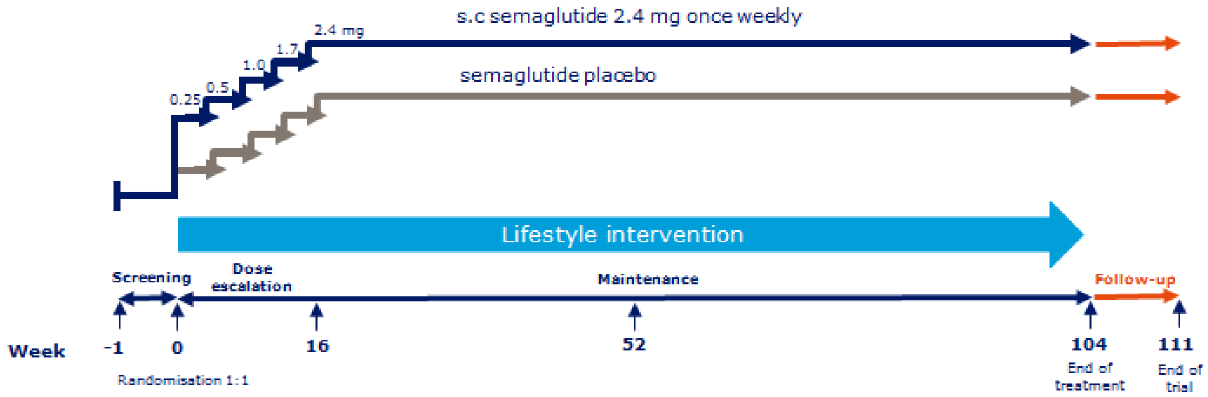

Figure 5-1 A schematic diagram of the trial design with duration of the trial periods including follow-up period.

Eligible subjects fulfilling all randomisation criteria at visit 2 will be randomised in a 1:1 manner to receive either semaglutide 2.4 mg once weekly or semaglutide placebo as an adjunct to a reduced-calorie diet and increased physical activity. A planned total of 300 subjects will be randomised

5.2 Subject and trial completion

Approximately 353 subjects will be screened to achieve 300 subjects randomly assigned to trial product.

Trial period completion for a subject:

Trial period completion is defined as when the randomised subject has completed the final scheduled visit ('end of trial' according to the flowchart).
'Date of trial completion' is the date the subject completed the final scheduled visit.

Treatment period completion for a subject:

Treatment period completion is defined as when the randomised subject has attended the 'end of treatment' visit according to the flowchart.

5.3 End of trial definition

The end of the trial is defined as the date of the last visit of the last subject in the trial.

5.4 Scientific rationale for trial design

The treatment duration of the trial is 104 weeks with an additional 7 weeks follow-up (without treatment). The 7 weeks follow-up period is included to account for the exposure and long half-life of semaglutide. A 104-week treatment duration (including 88 weeks on target dose) is considered sufficient to assess long-term weight loss, safety and tolerability in the phase 3 weight management development programme in accordance with regulatory guidelines[65, 66].

A randomised, double-blinded, placebo-controlled, multi-centre trial design is chosen to minimise bias in the assessment of the effect and safety of semaglutide 2.4 mg once-weekly versus semaglutide placebo, as an adjunct to a reduced calorie diet and increased physical activity.

5.5 Justification for dose

Results from the phase 2 dose-finding trial (NN9536-4153) showed that the semaglutide 0.4 mg once-daily dose was most effective in terms of weight loss while displaying an acceptable tolerability profile. Using population pharmacokinetic modelling, it was estimated that a once-weekly maintenance dose of 2.4 mg semaglutide will result in similar C_{max} at steady-state as that obtained by the once-daily 0.4 mg semaglutide dose in trial NN9536-4153.

A maintenance dose of 2.4 mg semaglutide once-weekly has been chosen for the phase 3 weight management development programme. The once weekly dosing is anticipated to ease the burden of drug administration in clinical practice. Subjects will be initiated at a once-weekly dose of 0.25 mg and follow a fixed-dose escalation regimen, with dose increases every 4 weeks (to doses of 0.5, 1.0, 1.7 and 2.4 mg/week), until the target dose is reached after 16 weeks.

It is well known that to mitigate GI side effects with GLP-1 RA treatment, dose escalation to the target dose is required. Based on experience from the semaglutide T2D development programme, a similar fixed-dose escalation regimen was selected, with dose escalation every 4 weeks until the target dose of 2.4 mg is reached after 16 weeks.

Please refer to Section 7.1 for more details on treatment doses.

6 Trial population

Prospective approval of protocol deviations to recruitment and enrolment criteria, also known as protocol waivers or exemptions, is not permitted.

6.1 Inclusion criteria

Subjects are eligible to be included in the trial only if all of the following criteria apply:

1. Informed consent obtained before any trial-related activities. Trial-related activities are any procedures that are carried out as part of the trial, including activities to determine suitability for the trial
2. Male or female, age ≥ 18 years at the time of signing informed consent
3. Body mass index (BMI) ≥ 30 kg/m^2 or ≥ 27 kg/m^2 with the presence of at least one of the following weight-related comorbidities (treated or untreated): hypertension, dyslipidaemia, obstructive sleep apnoea or cardiovascular disease
4. History of at least one self-reported unsuccessful dietary effort to lose body weight

The criteria will be assessed at the investigator's discretion unless otherwise stated.

For country specific requirements see Appendix 8

6.2 Exclusion criteria

Subjects are excluded from the trial if any of the following criteria apply:

Glycaemia-related:

1. HbA_{1c} ≥ 48 mmol/mol (6.5%) as measured by the central laboratory at screening
2. History of type 1 or type 2 diabetes mellitus
3. Treatment with glucose-lowering agent(s) within 90 days before screening

Obesity–related:

4. A self-reported change in body weight > 5 kg (11 lbs) within 90 days before screening irrespective of medical records
5. Treatment with any medication for the indication of obesity within the past 90 days before screening
6. Previous or planned (during the trial period) obesity treatment with surgery or a weight loss device. However, the following are allowed: (1) liposuction and/or abdominoplasty, if

performed > 1 year before screening, (2) lap banding, if the band has been removed > 1 year before screening, (3) intragastric balloon, if the balloon has been removed > 1 year before screening or (4) duodenal-jejunal bypass sleeve, if the sleeve has been removed > 1 year before screening

7. Uncontrolled thyroid disease, defined as TSH > 6.0 mIU/L or < 0.4 mIU/L as measured by the central laboratory at screening

Mental health:

8. History of major depressive disorder within 2 years before screening
9. Diagnosis of other severe psychiatric disorder (e.g., schizophrenia, bipolar disorder)
10. A Patient Health Questionnaire-9 (PHQ-9) score of ≥ 15 at screening
11. A lifetime history of a suicidal attempt
12. Suicidal behaviour within 30 days before screening
13. Suicidal ideation corresponding to type 4 or 5 on the Columbia-Suicide Severity Rating Scale (C-SSRS) within the past 30 days before screening

General safety:

14. Presence of acute pancreatitis within the past 180 days prior to the day of screening
15. History or presence of chronic pancreatitis
16. Calcitonin ≥ 100 ng/L as measured by the central laboratory at screening
17. Personal or first degree relative(s) history of multiple endocrine neoplasia type 2 or medullary thyroid carcinoma
18. Renal impairment measured as estimated Glomerular Filtration Rate (eGFR) value of eGFR < 15 ml/min/1.73 m^2 as defined by KDIGO 2012[67] by the central laboratory at screening
19. History of malignant neoplasms within the past 5 years prior to screening. Basal and squamous cell skin cancer and any carcinoma in-situ are allowed
20. Any of the following: myocardial infarction, stroke, hospitalisation for unstable angina or transient ischaemic attack within the past 60 days prior to screening
21. Subject presently classified as being in New York Heart Association (NYHA) Class IV
22. Surgery scheduled for the duration of the trial, except for minor surgical procedures, in the opinion of the investigator
23. Known or suspected abuse of alcohol or recreational drugs
24. Known or suspected hypersensitivity to trial product(s) or related products
25. Previous participation in this trial. Participation is defined as signed informed consent
26. Participation in another clinical trial within 90 days before screening
27. Other subject(s) from the same household participating in any semaglutide trial
28. Female who is pregnant, breast-feeding or intends to become pregnant or is of child-bearing potential and not using a highly effective contraceptive method
29. Any disorder, unwillingness or inability, not covered by any of the other exclusion criteria, which in the investigator's opinion, might jeopardise the subject's safety or compliance with the protocol

The criteria will be assessed at the investigator's discretion unless otherwise stated.
For country specific requirements please see Appendix 8

6.3 Randomisation criteria

Subjects are eligible to be randomised in the trial, only if all randomisation criteria apply:

1. Have kept a food diary satisfyingly in the period between screening and randomisation. This is defined as at least one entry per day. However, missed entries for a maximum of two days are allowed
2. A PHQ-9 score of < 15 at randomisation
3. No suicidal behaviour in the period between screening and randomisation
4. No suicidal ideation corresponding to type 4 or 5 on the C-SSRS in the period between screening and randomisation

Re-screening is not allowed. Subjects not fulfilling the randomisation criteria will be considered screen failure, see section 6.5.

6.4 Lifestyle restrictions

To ensure alignment in regards to performance of assessments across subjects and trial sites, the below restrictions apply.

6.4.1 Meals and dietary restrictions

- Subjects must attend the visits fasting according to the flowchart.
- Fasting is defined as at least 8 hours overnight before the visit, without food or liquids, except for water. Trial product and any medication which should be taken with or after a meal should be withheld on the day of the visit until blood samples have been obtained.
- If the subject is not fasting as required, the subject should be called in for a new visit within the visit window to have the fasting procedures done. Procedures requiring subject to fast include blood sampling of fasting plasma glucose (FPG), fasting serum insulin and free fatty acids.

6.4.2 Caffeine and tobacco

- Subject should avoid caffeine and smoking at least 30 minutes prior to measuring the blood pressure.

6.5 Screen failures

Screen failures are defined as subjects who consent to participate in the clinical trial but are not eligible for participation according to in/exclusion criteria or randomisation criteria. A minimal set of screen failure information is required to ensure transparent reporting of screen failure subjects to meet requirements from regulatory authorities. Minimal information includes date of informed

consent, date of visit, demography, screen failure details, eligibility criteria, and any serious adverse event (SAE). A screen failure session must be made in the interactive web response system (IWRS).

Individuals who do not meet the criteria for participation in this trial may not be rescreened. Re-sampling is not allowed if the subject has failed one of the inclusion criteria or fulfilled one of the exclusion criteria related to laboratory parameters. However, in case of technical issues (e.g. haemolysed or lost), re-sampling is allowed for the affected parameters.

7 Treatments

7.1 Treatments administered

- All trial products listed in Table 7-1 are considered investigational medicinal products (IMP).
- Trial product must only be used, if it appears clear and colourless.

Table 7-1 Trial products provided by Novo Nordisk A/S

**Trial product name:**	Semaglutide B 1.0 mg/mL PDS290 or semaglutide placebo*	Semaglutide B 3.0 mg/mL PDS290 or semaglutide placebo
**Dosage form:**	Solution for injection	Solution for injection
**Route of administration:**	Subcutaneous	Subcutaneous
**Dosing instructions:**	Once-weekly	Once-weekly
**Delivery device**	3 mL PDS290 pre-filled pen-injector	3 mL PDS290 pre-filled pen-injector

* Semaglutide B 1.0 mg PDS290/mL/semaglutide placebo will only be dispensed at the first dispensing visit.

- Dose escalation of semaglutide/semaglutide placebo should take place during the first 16 weeks after randomisation as described in Table 7-2. All subjects should aim at reaching the recommended target dose of 2.4 mg semaglutide once-weekly or the corresponding volume of placebo.
- If a subject does not tolerate the recommended target dose of 2.4 mg once-weekly, the subject may stay at the lower dose level of 1.7 mg once weekly. This should only be allowed if the subject would otherwise discontinue trial product completely and if considered safe to continue on trial product, as per the investigator's discretion. It is recommended that the subject makes at least one attempt to re-escalate to the recommended target dose of 2.4 mg once-weekly, as per the investigator's discretion.
- It is recommended that the investigator consults Novo Nordisk in case of persistent deviations from the planned escalation regimen.
- A dose reminder card will be handed out to the subjects at each site visit during the escalation period. This is to remind the subjects of the dose to be taken until next site visit and provide a

conversion of the dose to value shown in the dose counter. Once the target dose has been reached, the dose reminder card is only handed out as needed.

Table 7-2 Dose escalation and maintenance of semaglutide/semaglutide placebo

Trial product name	Dose	Volume	Value shown in dose counter*	Duration
**Dose escalation period**				
Semaglutide B 1.0 mg/mL PDS290 or semaglutide placebo	0.25 mg	0.25 mL	25*	4 weeks
Semaglutide B 1.0 mg/mL PDS290 or semaglutide placebo	0.5 mg	0.50 mL	50*	4 weeks
Semaglutide B 3.0 mg/mL PDS290 or semaglutide placebo	1.0 mg	0.34 mL	34*	4 weeks
Semaglutide B 3.0 mg/mL PDS290 or semaglutide placebo	1.7 mg	0.57 mL	57*	4 weeks
**Maintenance period**				
Semaglutide B 3.0 mg/mL PDS290 or semaglutide placebo	2.4 mg	0.80 mL	80*	88 weeks

* Conversion to dose is calculated on 0.01 mL/value for both strengths.

- Subjects will be instructed to inject semaglutide/semaglutide placebo once-weekly at the same day of the week (to the extent possible) throughout the trial.
- Injections may be administered in the thigh, abdomen or upper arm, at any time of day irrespective of meals.
- If a single dose of trial product is missed, it should be administered as soon as noticed, provided the time to the next scheduled dose is at least 2 days (48 hours). If a dose is missed and the next scheduled dose is less than 2 days (48 hours) away, the subject should not administer a dose until the next scheduled dose. A missed dose should not affect the scheduled dosing day of the week.
- If ≥ 2 consecutive doses of trial product are missed, the subject should be encouraged to re-commence the treatment if considered safe as per the investigator's discretion and if the subject does not meet any of the discontinuation criteria (Section 8.1). The trial product should be continued as early as the situation allows. The missed doses should not affect the scheduled dosing day of the week. The start dose for re-initiation of trial product is at the investigator's discretion. In case of questions related to re-initiation of trial product, the investigator should consult Novo Nordisk global medical experts.
- Auxiliary supplies will be provided in accordance with the trial materials manual (TMM), please see Table 7-3

Table 7-3 Auxiliary supplies provided by Novo Nordisk A/S

Auxiliary supply	Details
Needles	Needles for pre-filled pen system. Details provided in the TMM.<br>Only needles provided and approved by Novo Nordisk must be used for administration of trial product.
Direction for use (DFU)	DFU for 3 ml PDS290 pre-filled pen-injector<br>Not included in the dispensing unit and to be handed out separately

7.1.1 Medical devices

Information about the PDS290 pre-filled pen-injector may be found in the IB and any updates hereof[58].

Information about the use of the PDS290 pre-filled pen-injector for semaglutide 1.0 mg/mL, semaglutide 3.0 mg/mL, and semaglutide placebo can be found in the DFU.

Training in the PDS290 pre-filled pen-injector

The investigator must document that training in DFU has been given to the subjects orally and in writing at the first dispensing visit. Training must be repeated during the trial at regular intervals in order to ensure correct use of the medical device. Training is the responsibility of the investigator or a delegate.

7.1.2 Diet and physical activity counselling

All subjects in both treatment arms will receive counselling with regards to diet (500 kcal deficit per day relative to the estimated total energy expenditure (TEE) calculated once at randomisation) and physical activity (150 min of physical activity per week is encouraged e.g. walking or, use the stairs). Counselling should be done by a dietician or a similar qualified healthcare professional every 4th week via visits/phone contacts.

Subjects will be asked to record their food intake and physical activity at least 3 days prior to the phone contact and clinic visits according to the flowchart to assist their lifestyle intervention. However, the subjects should be encouraged to keep diary of their food intake and physical activity on a daily basis. After randomisation the subjects can use a tool of their own choice (paper/app/other tool) for recording, ensuring it can be reviewed during diet and physical activity counselling. Subjects must receive instructions in how to capture their physical activity and food intake.

Calculation of estimated TEE

The TEE is calculated by multiplying the estimated Basal Metabolic Rate (BMR) (see Table 7-4) with a Physical Activity Level value of 1.3. [68]

$$TTE = BMR \times 1.3$$

Table 7-4 Equation for estimated BMR

Sex	Age	BMR (kcal/day)
Men	18-30 years<br>31-60 years<br>> 60 years	15.057 × weight at randomisation in kg + 692.2<br>11.472 × weight at randomisation in kg + 873.1<br>11.711 × weight at randomisation in kg + 587.7
Women	18-30 years<br>31-60 years<br>> 60 years	14.818 × weight at randomisation in kg + 486.6<br>8.126 × weight at randomisation in kg + 845.6<br>9.082 × weight at randomisation in kg + 658.5

If a BMI ≤ 22.5 kg/m^2 is reached the recommended energy intake should be recalculated with no kcal deficit (maintenance diet) for the remainder of the trial. If deemed necessary the investigator could consult Novo Nordisk to discuss when maintenance diet can be initiated.

7.2 Dose modification

Not applicable for this trial. Please refer to Section 7.1 for description of missed dose(s).

7.3 Method of treatment assignment

All subjects will be centrally randomised using IWRS and assigned to the next available treatment according to randomisation schedule. Trial product will be dispensed at the trial visits summarised in the flowchart.

7.4 Blinding

The active drug and placebo are visually identical for the following trial products:

- Semaglutide B 1.0 mg/mL PDS290 / semaglutide placebo
- Semaglutide B 3.0 mg/mL PDS290 / semaglutide placebo

The IWRS is used for blind-breaking instructions. The blind may be broken in a medical emergency if knowing the actual treatment would influence the treatment of the subject. Novo Nordisk will be notified immediately after breaking the blind. The date when and reason why the blind was broken must be recorded in the subject's medical record.

Whenever the blind is broken, the person breaking the blind must print the "code break confirmation" notification generated by the IWRS, and sign and date the document.

When the blind is broken, the treatment allocation will be accessible to the investigator and the Novo Nordisk Global Safety department. If IWRS is not accessible at the time of blind break, the IWRS helpdesk should be contacted. Contact details are listed in Attachment I.

7.5 Preparation/Handling/Storage/Accountability

Only subjects enrolled in the trial may receive trial product and only authorised site staff may supply or administer trial product.

Table 7-5 Trial product storage conditions

Trial product name	Storage conditions (not-in-use)	In-use conditions	In-use time[a]
Semaglutide B 1.0 mg/mL PDS290<br>Semaglutide placebo	Store in refrigerator (2°C-8°C/36°F-46°F)<br>Do not freeze<br>Protect from light	In-use conditions will be available on the trial product label	In-use time will be available on the trial product label
Semaglutide B 3.0 mg/mL PDS290<br>Semaglutide placebo	Store in refrigerator (2°C-8°C/36°F-46°F)<br>Do not freeze<br>Protect from light	In-use conditions will be available on the trial product label	In-use time will be available on the trial product label

[a]In-use time starts when the product is taken out of the refrigerator in the subject's home.

- Each trial site will be supplied with sufficient trial products for the trial on an on-going basis controlled by the IWRS. Trial product will be distributed to the trial sites according to number of subjects screened and randomised.
- The investigator must confirm that appropriate temperature conditions have been maintained during transit for all trial products received (see Table 7-5) and any discrepancies are reported and resolved before use of the trial products.
- All trial products must be stored in a secure, controlled, and monitored (manual or automated) area in accordance with the labelled storage conditions with access limited to the investigator and authorised site staff.
- The investigator must inform Novo Nordisk immediately if any trial product has been stored outside specified conditions. Additional details regarding handling of temperature deviations can be found in the TMM.
- Trial product that has been stored improperly must not be dispensed to any subject before it has been evaluated and approved for further use by Novo Nordisk.
- The investigator is responsible for drug accountability and record maintenance (i.e. receipt, accountability and final disposition records).
- Drug accountability should be performed on a pen level and must be documented in the IWRS.
- The subject must return all used, partly used and unused trial product including empty packaging materials during the trial as instructed by the investigator.

- Destruction of trial products can be performed on an ongoing basis and will be done according to local procedures after accountability is finalised by the site and reconciled by the monitor.
- Destruction of trial products must be documented in the IWRS.
- All returned, expired or damaged trial products (for technical complaint samples, see Appendix 6) must be stored separately from non-allocated trial products. No temperature monitoring is required.
- Non-allocated trial products including expired or damaged products must be accounted as unused, at the latest at closure of the trial site.

7.6 Treatment compliance

Throughout the trial, the investigator will remind the subjects to follow the trial procedures and requirements to ensure subject compliance. If a subject is found to be non-compliant the investigator will remind the subject of the importance of following the instructions given, including taking the trial products as prescribed.

Treatment compliance of trial product will be assessed by asking subject about missed doses. Information about compliance and missed doses should be described in the subject's medical record, also see section 8.1.1.

7.7 Concomitant medication

Any medication (including over-the-counter or prescription medicines) other than the trial product that the subject is receiving at the time of the first visit or receives during the trial must be recorded along with:

- Trade name or generic name
- Indication
- Dates of administration including start and stop dates
- Dose (only to be recorded for anti-hypertensive and lipid-lowering medication)

During the trial subjects should not initiate any anti-obesity treatment (e.g. medication) which is not part of the trial procedures. If such anti-obesity treatment is initiated, the subject should be instructed to stop the anti-obesity treatment.

Changes in concomitant medication must be recorded at each visit. If a change is due to an AE, then this must be reported according to Section 9.2.

7.7.1 Rescue medication

This section is not applicable for this trial.

7.8 Treatment after the end of the trial

After the end of the trial the subject should be treated at the discretion of the investigator.

8 Discontinuation/Withdrawal criteria

The subject may be discontinued at any time during the trial at the discretion of the investigator for safety, behavioural, compliance or administrative reasons.

Efforts must be made to have the subjects, who discontinue trial product, to continue in the trial. Subjects must be educated about the continued scientific importance of their data, even if they discontinue trial product. Only subjects who withdraw consent will be considered as withdrawn from the trial.

8.1 Discontinuation of trial treatment

- Discontinuation of trial treatment can be decided by either the investigator or the subject.
- Subjects who discontinue trial product should continue with the scheduled visits and assessments to ensure continued counselling and data collection.
  - If the subject does not wish to attend the scheduled clinic visits efforts should be made to have the visits converted to phone contacts. However all efforts should be made to attend at least the 'end of treatment' clinic visit containing the final data collection of primary and confirmatory secondary efficacy endpoints, and the 'end of trial' visit.
- The 'end of trial' visit is scheduled approximately 7 weeks after the final data collection, to ensure the safety of the subject.
  - If the subject refuses to attend the 'end of treatment' and/or 'end of trial' visit, information about the attempts to follow up with the subject must be documented in the subject's medical record.

The subject must be discontinued from trial product, if any of the following applies:

1. Safety concern as judged by the investigator
2. Calcitonin ≥ 100 ng/L (see Appendix 7)
3. Suspicion of pancreatitis
4. Pregnancy
5. Intention of becoming pregnant
6. Simultaneous participation in another clinical trial of an approved or non-approved investigational medicinal product.

If acute pancreatitis is suspected appropriate management should be initiated, including local measurement of amylase and lipase (see Appendix 4 for reporting)

Subjects meeting discontinuation of trial product criterion no. 3 are allowed to resume trial product if the Atlanta criteria [69] are not fulfilled and thus, the suspicion of acute pancreatitis is not confirmed. Trial product may be resumed for subjects with a gallstone-induced pancreatitis in case of cholecystectomy.

Subjects meeting discontinuation of trial product criteria no. 1, 4 and 5 are allowed to resume trial product, if the criteria are no longer met (see Section 8.1.1).

The primary reason for discontinuation of trial product must be specified in the source data at the time of discontinuation, and subject should continue to follow the visit and assessment schedule. A change in 'treatment status' must be made in IWRS to discontinue trial product. If subject is not allowed to resume trial product, then the reason for discontinuation will be recorded in the 'end of treatment' form in the case report form (CRF), and final drug accountability must be performed.

8.1.1 Temporary discontinuation of trial treatment

If a subject has discontinued trial product due to temporary safety concern not related to trial product and is allowed to resume, the subject should follow the guide for missed doses (Section 7.1). Similarly, a subject who discontinue trial product on their own initiative should be encouraged to resume trial product (Section 7.1).

Each missed dose should be recorded in the case report form (CRF), as per subject's recollection. If a 'treatment status' session previously has been made in IWRS to indicate discontinuation of trial product, a new 'treatment status' session must be made to resume trial product.

8.2 Withdrawal from the trial

A subject may withdraw consent at any time at his/her own request.

If a subject withdraws consent, the investigator must ask the subject if he/she is willing, as soon as possible, to have assessment performed according to the 'end of treatment' visit. See the flowchart for data to be collected.

Final drug accountability must be performed even if the subject is not able to come to the trial site. The investigator must make a 'treatment status' session in IWRS to discontinue trial product.

If a subject withdraws from the trial, he/she may request destruction of any samples taken and not tested, and the investigator must document this in the medical record.

If the subject withdraws consent, Novo Nordisk may retain and continue to use any data collected before such a withdrawal of consent.

Although a subject is not obliged to give his/her reason(s) for withdrawing, the investigator must make a reasonable effort to ascertain the reason(s), while fully respecting the subject's rights. Where the reasons are obtained, the primary reason for withdrawal must be specified in the 'end of trial' form in the CRF.

8.2.1 Replacement of subjects

Subjects who discontinue trial product or withdraw from trial will not be replaced.

8.3 Lost to follow-up

A subject will be considered lost to follow-up if he or she repeatedly fails to return for scheduled visits and is unable to be contacted by the trial site.

The following actions must be taken if a subject fails to return to the trial site for a required visit:

- The site must attempt to contact the subject and reschedule the missed visit as soon as possible and counsel the subject on the importance of maintaining the assigned visit schedule and ascertain whether or not the subject wishes to and/or should continue in the trial.
- Before a subject is deemed lost to follow-up, the investigator must make every effort to regain contact with the subject (where possible, at least three telephone calls and, if necessary, a certified letter to the subject's last known mailing address or local equivalent methods). If attempts have failed, family members or other contacts consented by the subject can be contacted for alternative contact details. These contact attempts should be documented in the subject's source document.
- Should the subject continue to be unreachable at 'end of' treatment' visit, he/she will be considered to have withdrawn from the trial with a primary reason of 'lost to follow-up'.

9 Trial assessments and procedures

- Trial procedures and their timing are summarised in the flowchart.
- Informed consent must be obtained before any trial related activity, see Appendix 3.
- All screening evaluations must be completed and reviewed to confirm that potential subjects meet all eligibility criteria.
- The investigator will maintain a screening log to record details of all subjects screened and to confirm eligibility or record reason for screen failure, as applicable.
- At screening, subjects will be provided with a card stating that they are participating in a trial and giving contact details of relevant trial site staff.
- Adherence to the trial design requirements, including those specified in the flowchart, is essential and required for trial conduct.
- Assessments should be carried out according to the clinic's standard of practice unless otherwise specified in the current section. Efforts should be made to limit the bias between assessments. The suggested order of the assessments:
  1. Electrocardiogram (ECGs) and vital signs
  2. Blood samples
  3. Patient reported outcome questionnaires (see section 9.1.3) (applicable for US and Canada only) and mental health assessment instruments (see Section 9.4.1)
  4. Other assessments
- Source data of clinical assessments performed and recorded in the CRF must be available and will usually be the subject's medical records. Additional recording to be considered source data includes, but is not limited to laboratory reports, ECG, diary and recordings.

- Subject must receive instructions in how to capture their daily food intake in the handed out food diary from screening to randomisation. Entries must be evaluated in accordance with the randomisation criteria.
- Only the subject can make entries and corrections in the diaries, unless the section is specified for site staff.
- The barriers and motivation interview identifies barriers to and motivation for lifestyle change and compliance with the protocol. The interview must be conducted at screening to assist in identifying subjects who are unable or unwilling to comply with protocol procedures as per the exclusion criteria. In addition, the interview will ensure that any minor barriers are addressed during lifestyle counselling.
    - The results of the interview will not be entered into the CRF. It will be at the investigator's discretion to evaluate the motivation of the subject and related eligibility.
- Subject's weight history must be recorded in the subject's medical record.
- Review of mental health assessment instruments, ECG, laboratory reports must be documented either on the documents or in the subject's medical record. If clarification of entries or discrepancies is needed, the subject must be questioned and a conclusion made in the subject's source documents. Care must be taken not to bias the subject.
- Repeat laboratory samples may be taken for technical issues and unscheduled samples or assessments may be taken for safety reasons. Please refer to Appendix 2 for further details on laboratory samples.
- For subjects receiving antihypertensive or lipid-lowering treatment, the investigator should evaluate changes in the subjects' treatment intensity within each therapeutic area. The evaluation should be based on whether an overall change from randomisation until the time of the evaluation has occurred (i.e., either increase, decrease or no change) after reviewing all available relevant information e.g., changes in drug dose, drug class, number of drugs or a combination of these.

9.1 Efficacy assessments

Planned time points for all efficacy assessments are provided in the flowchart.

9.1.1 Body measurements

- Body weight should be measured at all site visits without shoes, on an empty bladder and only wearing light clothing. It should be measured on a digital scale and recorded in kilograms or pounds (one decimal) using the same scale throughout the trial. The scale must be calibrated yearly as a minimum.
- Height is measured without shoes in centimetres or inches (one decimal). BMI will be calculated by the CRF from screening data and must be in agreement with inclusion criterion no. 3.
- Waist circumference is defined as the abdominal circumference located midway between the lower rib margin and the iliac crest. Measures must be obtained in standing position with a non-

stretchable measuring tape and to the nearest cm or inch. The tape should touch the skin but not compress soft tissue and twists in the tape should be avoided. The subject should be asked to breathe normally. The same measuring tape should be used throughout the trial. The measuring tape will be provided by Novo Nordisk to ensure standardisation.

9.1.2 Clinical efficacy laboratory assessments

All protocol-required laboratory assessments, as defined in Appendix 2, must be conducted in accordance with the flowchart and the laboratory manual.

9.1.3 Clinical outcome assessment (applicable for US and Canada only)

Subjects should be given the opportunity to complete the questionnaire by themselves without interruption. The questionnaire takes approximately 10 minutes to complete.

The following patient reported outcome questionnaire will be used:

- Control of Eating Questionnaire (CoEQ) is designed to assess the intensity and type of food cravings, as well as subjective sensations of appetite and mood. For this trial a 19 items version will be used. The 19 individual items in the questionnaire are grouped into 4 domains: Craving Control, Positive Mood, Carving for Sweet, and Craving for Savoury. The questionnaire will be available in a linguistically validated translated version[70].

9.2 Adverse events

The definitions of AEs and SAEs can be found in Appendix 4.

The investigator is responsible for detecting, documenting, recording and following up on events that meet the definition of an AE or SAE.

9.2.1 Time period and frequency for collecting AE and SAE information

All AEs will be collected from the first trial-related activity after obtaining informed consent and until 'end of trial' visit, at the time points specified in the flowchart.

All SAEs will be recorded and reported to Novo Nordisk or designee within 24 hours, as indicated in Appendix 4. The investigator must submit any updated SAE data to Novo Nordisk within 24 hours of it being available.

Investigators are not obligated to actively seek for AE or SAE in former trial subjects. However, if the investigator learns of any SAE, including a death, at any time after a subject has been discontinued from/completed the trial, and the investigator considers the event to be possibly/probably related to the investigational trial product or trial participation, the investigator must promptly notify Novo Nordisk.

The method of recording, evaluating and assessing causality of AE and SAE and the procedures for completing and transmitting SAE reports are provided in Appendix 4.

Timelines for reporting of AEs including events for adjudication, Section 9.2.1.1, are listed in Figure 9-1.

Some AEs require additional data collection via a specific event form. This includes medication errors observed during the trial. The relevant specific events are listed in Table 9-1 and the reporting timelines in Figure 9-1

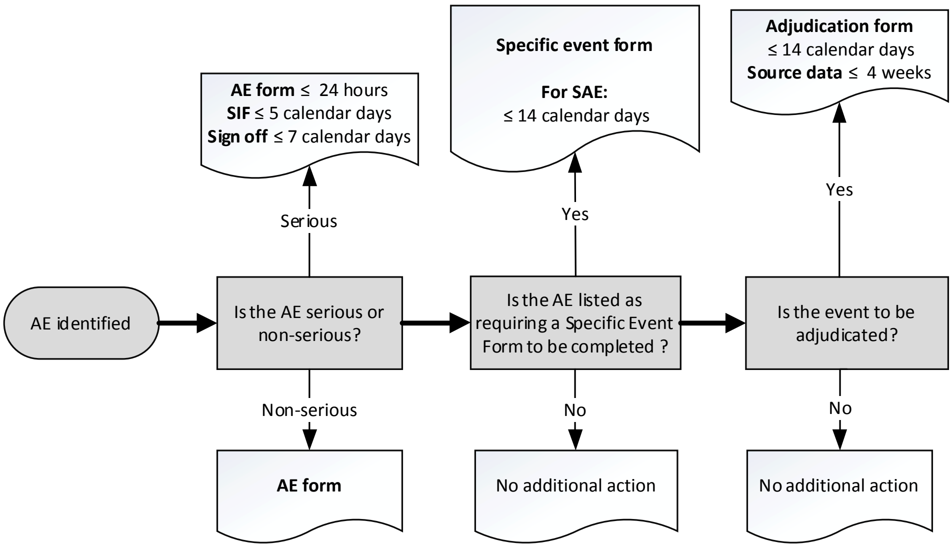

Timelines are from the awareness of an AE .
Queries to be resolved ≤ 14 calendar days.

AE: Adverse Events, SAE: Serious Adverse Events, SIF: Safety Information Form

Figure 9-1 Decision tree for determining the event type and the respective forms to complete with associated timelines

Table 9-1 AEs requiring additional data collection (via specific event form) and events for adjudication

Event type	AE via specific event form	Event for adjudication (Section 9.2.1.1)
Medication error<br>Misuse or abuse of trial product*	X	
Death		X
Cardiovascular events		
Acute Coronary Syndrome		X
Cerebrovascular event		X
Heart failure		X

Coronary artery revascularisation		X
Acute pancreatitis	X	X
Acute gallbladder disease	X	
Malignant neoplasms	X	
Hepatic event	X	

*Additional data for Misuse or abuse of trial product is reported on the medication error event form.

9.2.1.1 Events for adjudication

Event adjudication will be performed for adverse events in randomised subjects. These events are reviewed by an independent external event adjudication committee (EAC) in a blinded manner, refer to Appendix 3 for further details.

There are four ways to identify events relevant for adjudication as described below:

- Investigator-reported events for adjudication: When reporting AEs, the investigator must select the appropriate AE category based on pre-defined criteria (see Table 9-1 and Appendix 4)
- Death
- AE Search (standardised screening): All AEs not directly reported by the investigator as requiring adjudication, will undergo screening to identify potential events for adjudication. The investigator can be queried to provide additional information related to the reported AE, e.g. alternative aetiology, underlying cause(s) and/or clinical details.
- EAC-identified events: When reviewing source documents provided for another event for adjudication, the EAC can identify additional events in scope for adjudication that were not initially reported by the investigator. In these instances, the investigator will be notified of the newly identified event and has the option to report the EAC-identified event. Regardless of whether the investigator decides to report the event, it will undergo adjudication. Occasionally, EAC-identified events may require the investigator to collect additional source documents, which should be provided by uploading to the event adjudication system (EAS).

With the exception of EAC-identified events, an event-specific adjudication form for the event in question should be completed in the CRF within 14 calendar days of the investigator's first knowledge of the event.

Copies of collected source documents should be labelled with trial ID, subject and AE number, redacted (anonymised of personal identifiers) and uploaded to the event adjudication system within 4 weeks according to instructions outlined in the event adjudication site manual. If no, or insufficient source documents are provided to the adjudication supplier, the investigator can be asked to complete a clinical narrative to be uploaded to the EAS.

If new information becomes available for an event sent for adjudication, it is the responsibility of the investigator to ensure the new information is uploaded to the EAS.

9.2.2 Method of detecting AEs and SAEs

Care should be taken not to introduce bias when detecting AEs and/or SAEs. Open-ended and non-leading verbal questioning of the subject is the preferred method to inquire about events.

9.2.3 Follow-up on AEs and SAEs

After the initial AE/SAE report, the investigator is required to proactively follow each subject at subsequent visits/contacts. All SAEs will be followed until resolution, stabilization, or if the event is otherwise explained (e.g. chronic condition) or the subject is lost to follow-up (as defined in Section 8.3). Further information on follow-up procedures is given in Appendix 4.

9.2.4 Regulatory reporting requirements for SAEs

Prompt notification by the investigator to Novo Nordisk of a SAE is essential so that legal obligations and ethical responsibilities towards the safety of subjects and the safety of a trial product under clinical investigation are met.

Novo Nordisk has a legal responsibility to notify both the local regulatory authority and other regulatory agencies about the safety of a trial product under clinical investigation. Novo Nordisk will comply with country-specific regulatory requirements relating to safety reporting to the regulatory authority, IRB/IEC, and investigators.

Investigator safety reports must be prepared for suspected unexpected serious adverse reactions (SUSARs) according to local regulatory requirements and Novo Nordisk policy and forwarded to investigators as necessary.

An investigator who receives an investigator safety report describing a SAE or other specific safety information (e.g. summary or listing of SAEs), from Novo Nordisk will review and then file it along with the IB and will notify the IRB/IEC, if appropriate according to local requirements.

9.2.5 Hypoglycaemic, cardiovascular and death events

Hypoglycaemic, cardiovascular and death events will be handled and reported according to AE/SAEs description in Section 9.2.1.

9.2.6 Disease-related events and/or disease-related outcomes not qualifying as an AE or SAE

Not applicable for this trial.

9.2.7 Pregnancies and associated adverse events

Details of pregnancies in female subjects will be collected after the first-trial-related activity after obtaining informed consent and until the 'end of trial' visit.

If a pregnancy is reported in female subjects, the investigator should inform Novo Nordisk within 14 calendar days of learning of the pregnancy and should follow the procedures outlined in Figure 9-2and Appendix 5.

Pregnancy outcome should be documented in the subject's medical record. Abnormal pregnancy outcome (e.g. spontaneous abortion, foetal death, stillbirth, congenital anomalies and ectopic pregnancy) is considered an SAE.

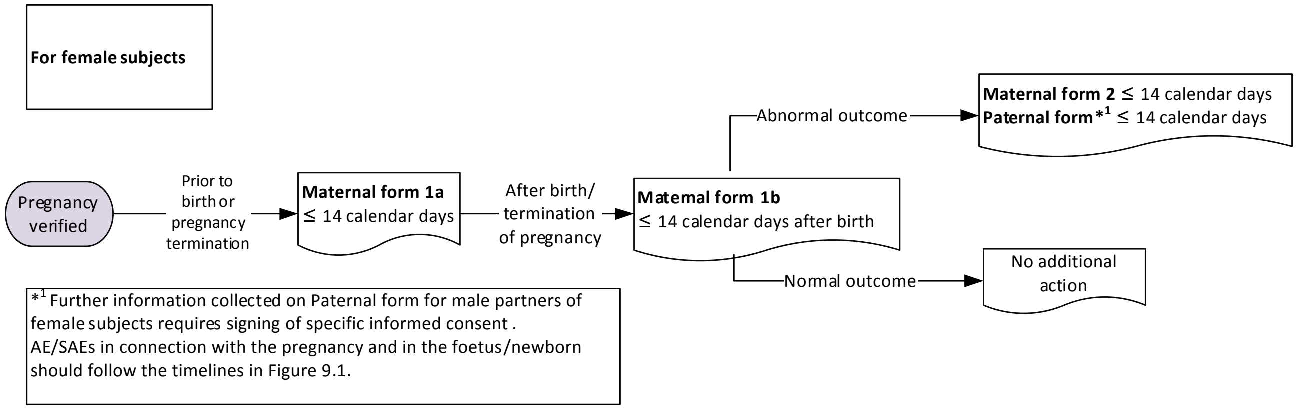

Figure 9-2 Decision tree for determining the forms to complete with associated timelines for pregnancy.

9.2.8 Medical device incidents (including malfunctions)

Section is not applicable for this trial. Refer to technical complaints in Section 9.2.9

9.2.9 Technical complaints

The investigator must assess whether a technical complaint is related to an AE.

The definitions and reporting process for technical complaints can be found in Appendix 6.

Timelines for reporting technical complaints are listed in Figure 9-3

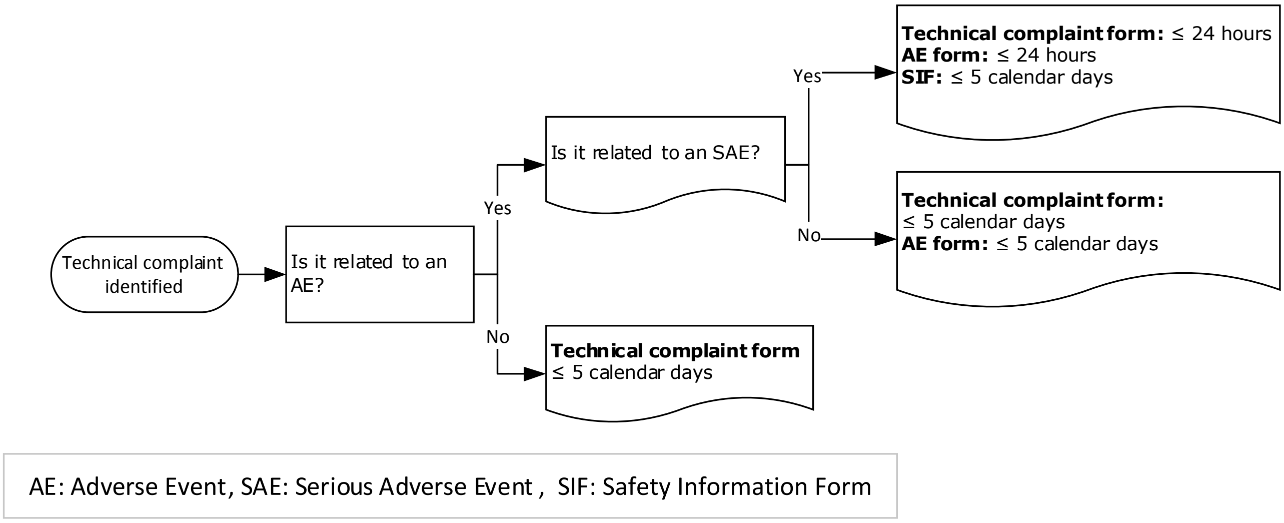

Figure 9-3 Decision tree for determining the forms to complete with associated timelines for technical complaints.

9.3 Treatment of overdose

Overdoses of up to 4 mg in a single dose, and up to 4 mg in a week have been reported in clinical trials. The most commonly reported AE was nausea. All subjects recovered without complications.

There is no specific antidote for overdose with semaglutide. In the event of an overdose, appropriate supportive treatment should be initiated according to subject's clinical signs and symptoms.

The overdose must be reported as a medication error (Appendix 4) and for reporting times see Section 9.2.1 and Figure 9-1.

In the event of an overdose, the investigator should closely monitor the subject for overdose-related AE/SAE and laboratory abnormalities. A prolonged period of observation and treatment may be necessary, taking into account the long half-life of semaglutide of approximately one week.

Decisions regarding dose interruptions or modifications will be made by the investigator based on the clinical evaluation of the subject.

For more information on overdose, also consult the current version of the IB[58] and any updates hereof.

9.4 Safety assessments

Planned time points for all safety assessments are provided in the flowchart.

A **concomitant illness** is any illness that is present at the start of the trial (i.e. at the first visit) or found as a result of a screening procedure or other trial procedures performed before exposure to trial product.

Medical history is a medical event that the subject has experienced in the past. Only relevant and significant medical history as judged by the investigator should be recorded. Findings of specific medical history should de described in designated forms.

As part of the medical history information related to history of gallbladder disease, breast neoplasm, colon neoplasm, skin cancer and psychiatric disorder will be recorded. Follow-up questions will be asked at the end of trial related to the breast neoplasm and colon neoplasm.

In case of an abnormal and clinically significant finding, the investigator must record the finding on the Medical History/Concomitant Illness form if it is present at screening. Any new finding fulfilling the AE definition (see Appendix 4) during the trial and any clinically significant worsening from baseline must be reported as an AE (see Section 9.2).

9.4.1 Mental health assessment instruments

- PHQ-9[71] is a 9-item depression module of the patient health questionnaire, which is a self-administered diagnostic tool used for assessment of mental disorders. The questionnaire will be available in a linguistically validated translated version.
- C-SSRS[72] is a detailed questionnaire assessing both suicidal behaviour and suicidal ideation. The questionnaire will be administered as an interview by the investigator or a qualified delegate. The questionnaire (C-SSRS Baseline and C-SSRS Since Last Visit) will be available in a linguistically validated translated version.
  - Prior to administering the C-SSRS questionnaire, the investigator or qualified delegate must complete sufficient training.

If a subject has a PHQ-9 score of 10-14 both inclusive the subject should be referred to a mental health professional (MHP) if judged relevant by the investigator. If referral is not deemed relevant this, along with the reason why, must be documented in the subject's medical records.

A subject must be referred to a MHP if:
- the subject has a PHQ-9 score ≥15 or
- the subject has any suicidal behaviour or
- the subject has any suicidal ideation of type 4 or type 5 on any C-SSRS assessment or
- in the opinion of the investigator, it is necessary for the safety of the subject

If one or more of the referral criteria are met, the investigator should explain to the subject why the referral and psychiatric evaluation by a MHP is needed. If the subject refuses to be referred to a MHP, the subject's decision should be documented in subject's medical record and the investigator

must assess if it is safe for the subject to continue in the trial or if the subject should be discontinued from trial product.

If a subject's psychiatric disorder can be adequately treated with psychotherapy and/or pharmacotherapeutic treatment, then the subject, at the discretion of the investigator (and in agreement with the MHP), may continue in the trial. Otherwise, the subject must be discontinued from trial product due to safety concern as judged by the investigator.

9.4.2 Physical examinations

- A physical examination will include assessments of the general appearance, thyroid gland, breast (females), and abdomen, as well as the cardiovascular and respiratory systems.
- Investigators should pay special attention to clinical signs related to previous serious illnesses.

9.4.3 Vital signs

- The method for measuring systolic and diastolic blood pressure needs to follow the standard clinical practice at site.
- Blood pressure (diastolic and systolic) and pulse measurements should be preceded by at least 5 minutes of rest for the subject in a quiet setting without distractions (e.g. television, cell phones).
- Blood pressure and pulse measurements will be assessed in a sitting position with a completely automated device. Manual techniques will be used only if an automated device is not available.

9.4.4 Electrocardiograms

- 12-lead ECG will be obtained as outlined in the flowchart using a local ECG machine that automatically calculates the heart rate and measures PR, QRS and QT intervals.

9.4.5 Clinical safety laboratory assessments

All protocol-required laboratory assessments, as defined in Appendix 2, must be conducted in accordance with the flowchart and the laboratory manual.

- If the laboratory finding based on the results from central laboratory meets the criteria for laboratory outliners, a laboratory outliner form in the CRF should be completed. Please refer to Appendix 2 for the criteria for the laboratory outliners.

- Urine pregnancy tests provided by central laboratory must be performed for women of childbearing potential at screening and as specified in the flowchart. Urine pregnancy test must be repeated at any time during the trial if pregnancy is suspected. Further instructions can be found in the laboratory manual.

9.5 Pharmacokinetics

Not applicable for this trial.

9.6 Pharmacodynamics

Not applicable for this trial.

9.7 Genetics

Not applicable for this trial.

9.8 Biomarkers

Collection of samples for biomarker research is part of this trial to support the effect objectives. The following samples must be conducted in accordance with the laboratory manual and the flowchart:

Biomarkers linked to cardiovascular risk

- High sensitive C-reactive protein (hsCRP)

9.9 Severe hypersensitivity

In the event of a severe immediate hypersensitivity reaction to trial product, blood sampling for assessment of anti-semaglutide IgE and binding antibodies should be conducted after 1–2 weeks and 7 weeks of trial product wash-out (i.e. after the subject had the last dose of the trial product).

In these cases, it is also recommended to test for tryptase (total and/or mature tryptase) within 3 hours of the hypersensitivity reaction. In case a tryptase sample was collected within 3 hours of the event of hypersensitivity reaction, a baseline tryptase sample should be taken at the same time as the IgE sample is obtained (after 1-2 weeks of trial product wash-out). Tryptase concentrations (if measured) as well as results of anti-semaglutide antibody and IgE isotype anti-semaglutide antibodies will be collected by Novo Nordisk and the results will be reported in the CTR.

10 Statistical considerations

Taxonomy of week 104 assessments

For each subject a given assessment at week 104 may be available or missing and Table 10-1 describes the taxonomy for this. Note, this is done per assessment and per subject; subjects may be a different type for different assessments (a subject may have "available on randomised treatment (AT)" for body weight but "missing on randomised treatment (MT)" for waist circumference).

Table 10-1 Taxonomy for subjects based on week 104 assessments

Assessment at week 104	Subjects on randomised treatment at week 104	Type description	Type Abbreviation
Available	Yes	**Available on randomised treatment**:<br>Subjects who complete the trial on randomised treatment with an assessment at week 104: Includes those that stop and restart trial product.	AT
	No	**Available but discontinued**<br>Subjects who discontinued randomised treatment prematurely but returned to have an assessment at week 104. These are also called retrieved subjects	AD
Missing	Yes	**Missing on randomised treatment**:<br>Subjects who complete the trial on randomised treatment without an assessment at week 104: Includes those that stop and restart trial product.	MT
	No	**Missing and discontinued**:<br>Subjects who discontinued randomised treatment prematurely and did not return to have an assessment at week 104. These are also called non-retrieved subjects	MD

10.1 Sample size determination

The sample size was primarily defined to ensure sufficient power for the two primary endpoints. Given the trial sample size, the power of statistical tests for all confirmatory endpoints is described below.

The tests of superiority of semaglutide 2.4 mg to semaglutide placebo for the primary and confirmatory secondary endpoints are performed using the fixed-sequence statistical strategy. This strategy tests the endpoints using a predefined hierarchical order, all at the significance level of 5%, moving to test the next endpoint only after a statistically significant superiority result (p-value < 5%) on the previous endpoint. The test hierarchy is given in Table 10-2 with underlying assumptions, marginal power and effective power. The effective power is calculated under the assumption of independence of endpoints by multiplying the respective marginal powers successively. As the two primary endpoints are included in the statistical testing hierarchy, significant superiority of semaglutide 2.4 mg vs. semaglutide placebo must be demonstrated for each of the primary endpoints.

In the analysis approach addressing the primary estimand, week 104 assessments from retrieved subjects (AD) are used. These data are also used to impute missing measurements at week 104 for non-retrieved subjects (MD). The imputation is done separately within each treatment arm (see

description below). However, for the power calculations missing values (MT and MD), regardless of treatment arm, are assumed to be similar to semaglutide placebo subjects. These assumptions are likely conservative with respect to the power, and correspond to the jump to reference sensitivity analysis planned below.

Assumptions

The common assumptions for the power calculations are

- The significance level is 5%
- The randomisation ratio is 1:1
- For continuous endpoints the t-test on the mean difference assuming equal variances is used
- For binary endpoints the Pearson chi-square test for two independent proportions is used
- 40% of subjects discontinue permanently and 50% of these are retrieved (AD) at week 104
- All subjects in the semaglutide placebo arm are assumed to have same effect as subjects who complete the trial on semaglutide placebo (AT)
- Retrieved subjects (AD) in the semaglutide 2.4 mg arm are assumed to have an effect corresponding to 25% of the treatment difference (compared to semaglutide placebo) of subjects who complete the trial on semaglutide 2.4 mg (AT)
- Non-retrieved subjects (MD) in the semaglutide 2.4 mg arm are assumed to have an effect corresponding to semaglutide placebo

Further assumptions made to calculate the power for each of the primary and confirmatory secondary endpoints are based on findings from other projects conducted by Novo Nordisk (NN8022 (SCALE), NN9535 (SUSTAIN), NN9924 (PIONEER)), and trial NN9536-4153 and are presented in Table 10-2. For weight, waist circumference and systolic blood pressure a 2 year completer was assumed to have 90% the effect of a 1 year completer.

Given these assumptions, the sample size of 300 subjects (150 in each arm), gives an effective power (marginal powers multiplied) of 43%. As sample size is primarily driven by the two primary endpoints, additional scenarios for assumptions are not included due to the high power for these endpoints.

Table 10-2 Assumptions, marginal power and effective power for each endpoint in the hierarchical testing procedure given an anticipated number of 300 randomised subjects

Order	Endpoint	Assumed mean (±SD) or proportion for completers		Expected mean (±SD) or proportion Semaglutide 2.4 mg	Expected difference or proportion ratio	Marginal power (%)	Effective power (%)
		Semaglutide 2.4 mg	Semaglutide placebo				
1	% weight change #	12.6 (±11)	3.0 (±11)	9.2 (±12)	6.2%-points	> 99	> 99
2	5% responders	76%	43%	64%	1.5	96	96
3	10% responders	59%	26%	48%	1.8	97	93
4	15% responders	41%	14%	31%	2.2	96	89
5	WC change (cm) #	9.3 (±11)	4 (±11)	7.4 (±12)	3.4 cm	69	62
6	sBP change (mmHg) #	7.6 (±13)	1.5 (±13)	5.5 (±14)	4 mmHg	69	43

SD = standard deviation; WC = waist circumference; sBP = systolic blood pressure; # shown as a positive number

10.2 Definition of analysis sets

Two analysis sets are defined:

- The *full analysis set* (FAS) includes all randomised subjects according to the intention-to-treat principle.
- The *safety analysis set* (SAS) includes all randomised subjects exposed to at least one dose of randomised treatment.

Any observation excluded from the analysis will be documented before database lock with the reason for exclusion provided.

Two observation periods are defined for each subject:

- In-trial: The *in-trial period* is defined as the uninterrupted time interval from date of randomisation to date of last contact with trial site.
- On-treatment (with trial product): A time-point is considered as 'on-treatment' if any dose of trial product has been administered within the prior 2 weeks (14 days). The *on-treatment period* is defined as all times which are considered on-treatment.
  - In general, the on-treatment period will therefore be from the date of first trial product administration to date of last trial product administration excluding potential off-treatment time intervals triggered by at least two consecutive missed doses.
  - For the evaluation of adverse events the lag time for each on-treatment time interval is 7 weeks (49 days)

The in-trial and on-treatment periods define the patient years of observation (PYO) and patient years of exposure (PYE), respectively, as the total time duration in the periods.

10.3 Statistical analyses

If necessary, a statistical analysis plan (SAP) may be written in addition to the protocol, including a more technical and detailed elaboration of the statistical analyses. The SAP will be finalised before database lock.

Effect endpoints will be analysed using the FAS; safety endpoints will be analysed using the SAS.

Results from statistical analyses will generally be accompanied by two-sided 95% confidence intervals and corresponding p-values. Superiority will be claimed if p-values are less than 5% and the estimated treatment contrasts favours semaglutide 2.4 mg.

Handling of missing baseline data

The last available and eligible observation at or before randomisation, is used as the baseline value. If no assessments are available, the mean value at randomisation across all subjects is used as the baseline value.

10.3.1 Primary endpoint

Definition of primary endpoint: % weight change

Change from baseline (week 0) to week 104 in body weight (%) is defined as

$$\%\text{ weight change} = \frac{(\text{body weight at week 104} - \text{body weight at baseline})}{\text{body weight at baseline}} \times 100.$$

Definition of primary endpoint: 5% responders

A body weight reduction of at least 5% from baseline (week 0) to week 104 is defined as

$$5\%\text{ responder} = \begin{cases} 1 \text{ if } \%\text{ weight change} \le -5\% \\ 0 \text{ if } \%\text{ weight change} > -5\% \end{cases}.$$

Analyses addressing the primary estimand

The following statistical analyses and imputation methods are designed to address the primary estimand, i.e. to assess the effectiveness of semaglutide 2.4 mg.

The analysis model for % weight change is a linear regression (ANCOVA) of % weight change with randomised treatment as a factor and baseline body weight (kg) as covariate. The estimated treatment difference between semaglutide 2.4 mg and semaglutide placebo will be reported together with the associated two-sided 95% CI and corresponding p-value.

The analysis model for the 5% responder endpoint is a logistic regression using randomised treatment as a factor and baseline body weight (kg) as covariate. The estimated odds ratio (OR)

between semaglutide 2.4 mg and semaglutide placebo will be reported together with the associated two-sided 95% CI and corresponding p-value.

The superiority tests of semaglutide 2.4 mg vs. semaglutide placebo will be carried out as follows for the two analysis models.

Let $\mu_{semaglutide}$ and $\mu_{semaglutide\ placebo}$ denote the true mean of % weight change for semaglutide 2.4 mg and semaglutide placebo group, respectively. The null and alternative hypotheses tested are

$$H: \mu_{semaglutide} \geq \mu_{semaglutide\ placebo}\ vs$$
$$H_A: \mu_{semaglutide} < \mu_{semaglutide\ placebo}.$$

The hypothesis will be rejected and superiority claimed, if the upper limit of the estimated two-sided 95% CI is below 0.

Let $OR_{semaglutide/semaglutide\ placebo}$ denote the true odds ratio between semaglutide 2.4 mg and semaglutide placebo. The null and alternative hypotheses tested are

$$H: OR_{semaglutide/semaglutide\ placebo} \leq 1\ vs$$
$$H_A: OR_{semaglutide/semaglutide\ placebo} > 1.$$

The hypothesis will be rejected and superiority claimed, if the lower limit of the estimated two-sided 95% CI is above 1.

Handling of missing week 104 values for the primary estimand

All available data at week 104 (AT and AD) are used and missing values (MT and MD) at week 104 will be imputed and the endpoints will be derived from the imputed values. Several approaches for imputation will be applied. First, a description of the primary imputation approach to address the primary estimand for the primary endpoints is given followed by a description of the sensitivity analyses used to assess the robustness of the primary analysis results. The sensitivity analyses investigate how assumptions on body weight development after discontinuation of randomised treatment impact the estimated treatment contrasts between semaglutide 2.4 mg and semaglutide Figure 10-1.

Primary imputation approach for the primary estimand

Multiple imputation approach using retrieved subjects (RD-MI): The primary imputation approach for the primary estimand is a multiple imputation similar to the one described by McEvoy[73] . Missing body weight measurement at week 104 for non-retrieved subjects (MD) are imputed using assessments from retrieved subjects (AD) in each randomised treatment arm. This will be done according to the timing of last available observation (LAO) of body weight. Missing body weight measurements at week 104 for subjects on randomised treatment (MT) are imputed by sampling from available measurements at week 104 from subjects on randomised treatment (AT) in the relevant randomised treatment arm. The multiple imputation approach is done in three steps:

1. **Imputation**: Defines an imputation model using retrieved subjects (AD) from FAS and done within groups defined by randomised treatment and the timing of the LAO of body weight. The model will be a linear regression of body weight (kg) at week 104 with gender (male/female), baseline BMI (kg/m^2) (in categories 27-<35, 35-<40, ≥40) as factors and baseline body weight (kg) and LAO of body weight (kg) as covariates. No interactions will be included. The grouping of timing will be done by quarters (intervals of 26 weeks for endpoints evaluating the change after 104 weeks, intervals of 13 weeks for endpoints evaluating the change after 52 weeks). If timing by quarters is too restrictive, halves (intervals of 52 weeks for endpoints evaluating the change after 104 weeks, intervals of 26 weeks for endpoints evaluating the change after 52 weeks) or excluding timing will be used. If any subjects are MT, an imputation model for missing body weight measurements at week 104 for MT subjects will also be defined using AT subjects in a similar way. The estimated posterior distribution for the parameters (regression coefficients and variances) in the imputation models are then used to impute missing week 104 body weight values for each randomised treatment arm. This will be done 1,000 times and results in 1,000 complete data sets.
2. **Analysis**: Analysis of each of the 1,000 complete data sets, using the analysis models (ANCOVA and logistic regression) results in 1,000 times 2 estimations.
3. **Pooling**: Integrates the 1,000 times 2 estimation results into two final results using Rubin's formula.

Based on NN9536-4153 phase 2 results 1,000 copies should be sufficient to establish stable results. If 1,000 copies are insufficient, 10,000 copies will be used. The multiple imputations will be generated using Novo Nordisk trial number 95364378 as seed number.

Sensitivity analyses

Jump to reference multiple imputation approach (J2R-MI): Missing values of body weight at week 104 (MT and MD) for both the semaglutide 2.4 mg and semaglutide placebo group are imputed by sampling among all available assessments at week 104 in the semaglutide placebo group (AT and AD). This approach makes the assumption that subjects instantly after discontinuation lose any effect of randomised treatment beyond what can be expected from semaglutide placebo treatment as adjunct to reduced-calorie diet and increased physical activity[74]. The multiple imputation approach is done as above with the first step replaced by

1. **Imputation**: Defines an imputation model using semaglutide placebo subjects from FAS with a week 104 measurement (AT and AD). The model will be a linear regression of body weight (kg) at week 104 with gender (male/female), BMI (kg/m^2) (in categories 27-<35, 35-<40, ≥40) as factors and baseline body weight (kg) as covariate. No interactions will be included. The estimated posterior distribution for the parameters (regression coefficients and variances) in the imputation models are then used to impute missing week 104 body weight values for each randomised treatment arm. This will be done 1,000 times and results in 1,000 complete data sets.

The jump to reference approach is the basis for the sample size calculations.

A single imputation approach as done by Sacks[75] *(S1-SI and S2-SI)*: Missing weight measurements at week 104 for non-retrieved subjects (MD) are imputed using a weight regain rate of 0.3 kg/month after LAO but truncated at no change from baseline whenever the extrapolation would lead to a positive weight gain relative to baseline. If a subject's weight at drug discontinuation represented a gain in weight relative to baseline, no additional gain will be imputed, and the unfavourable gain is carried forward to week 104. The weight regain imputation will be done for both randomised arms (S1-SI). Additionally, a version where only the semaglutide 2.4 mg arm uses the regain rate while the semaglutide placebo arm uses last available observation (corresponding to a weight regain rate of 0 kg/month) will be performed (S2-SI). For both versions, missing weight measurements at week 104 for subjects on randomised treatment (MT) are imputed by using LAO.

Tipping-point multiple imputation analysis (TP-MI): First, missing data are imputed according to the primary multiple imputation approach. Second, for the semaglutide 2.4 mg treatment arm a penalty will be added to the imputed values at week 104. The approach is to gradually increase this penalty until all confirmed conclusions from the primary analysis are reversed. For each hypothesis tested the specific value of the penalty that reverses the conclusion will be used to evaluate the robustness of the primary analysis results. This sensitivity analysis evaluates the robustness of the superiority conclusions.

Mixed model for repeated measurements (MMRM): This 'MMRM for effectiveness' will use all assessments regardless of adherence to randomised treatment, including assessments at week 104 for retrieved drop-outs (AD). The MMRM for effectiveness will be fitted using the same factor and covariate as for the primary analyses all nested within visit. An unstructured covariance matrix for measurements within the same subject will be employed, assuming that measurements for different subjects are independent.

Non-retrieved subjects as non-responders: For the 5% responder analysis an analysis using non-retrieved subjects as non-responders in the logistic regressions will be done.

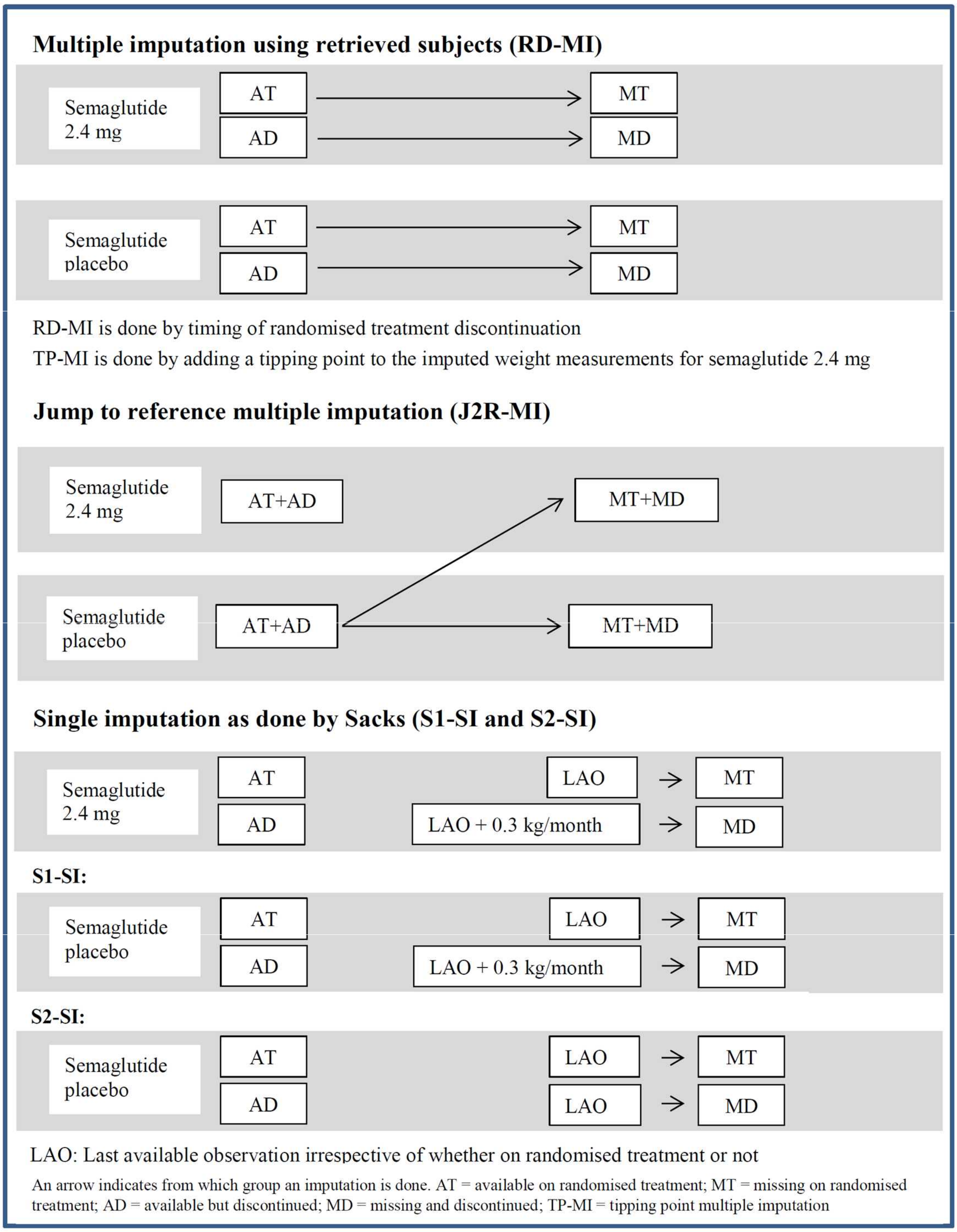

Figure 10-1 Illustration of imputation approaches for the effectiveness estimand

Analysis addressing the secondary estimand

The secondary estimand for % weight change addresses the efficacy of semaglutide 2.4 mg and will be assessed using a 'MMRM for efficacy'. Week 104 assessments for retrieved drop-outs (AD) are not used in this analysis. The MMRM for efficacy will use assessments only from subjects who are taking the randomised treatment until end of treatment or until first discontinuing of randomised treatment. The derived date of the second consecutive missed dose will be used as the latest date for using assessments in this MMRM. The assessment closest in time and before the derived date of the second consecutive missed dose will be used as last assessment on randomised treatment. For subjects who initiate rescue interventions before completion or first discontinuing of randomised treatment, the date of starting weight management drugs or undergoing bariatric surgery will be used as latest date for using assessments in this MMRM. Similarly, the assessment closest in time and before the date of starting weight management drugs or undergoing bariatric surgery will be used as last assessment on randomised treatment. The MMRM for efficacy will be fitted using % weight change and the same factor and covariate as for the primary analyses all nested within visit. An unstructured covariance matrix for measurements within the same subject will be employed, assuming that measurements for different subjects are independent.

The secondary estimand for 5% responders will be assessed using the same MMRM for efficacy. From the MMRM individually predicted values for % weight change at week 104 will be used to classify each subject as 5% responder or not. This classification will then be analysed using a logistic regression model with treatment as the only factor.

An overview of all analysis and imputation methods to address the effectiveness and efficacy estimands for the primary endpoints is given in Table 10-3

10.3.2 Secondary endpoints

10.3.2.1 Confirmatory secondary endpoints

Confirmatory secondary endpoints are listed in section 4 and are all included in the fixed-sequence statistical strategy, see above. All tests are tests of superiority of semaglutide 2.4 mg to semaglutide placebo.

Analyses addressing the primary estimand

All confirmatory secondary endpoints will be analysed using the same imputation approach as used for the primary endpoints and to address the primary estimand. The imputation model is the same as for the primary endpoints with body weight replaced by assessments of the endpoint to be analysed. The statistical model for continuous endpoints will be ANCOVA with factor and covariate as for the primary endpoint % weight change with baseline body weight replaced by the baseline assessment of the endpoint to be analysed. The statistical model for body weight responder endpoints will be logistic regression with factors and covariate as for the primary endpoint 5% responders.

Analyses addressing the secondary estimand

The confirmatory secondary endpoints which relate to the primary objective will be analysed to address the secondary estimand using the same MMRM for efficacy described for the primary endpoints.

Sensitivity analyses for confirmatory secondary endpoints

For all continuous confirmatory secondary endpoints a sensitivity analysis using jump to reference as imputation approach will be carried out. For all binary confirmatory secondary endpoints a sensitivity analysis using non-retrieved subjects as non-responders will be carried out.

An overview of all analysis and imputation methods to address the effectiveness and efficacy estimands for confirmatory secondary endpoints is given in Table 10-3.

Table 10-3 Analysis and imputation methods to address the effectiveness and efficacy estimands for the primary and confirmatory secondary endpoints in the statistical testing hierarchy

Objective	Endpoint	Test order	Endpoint type	Estimand	Analysis set	Statistical model	Imputation approach	Sensitivity analyses
**Primary endpoints**								
Primary	% weight change	1	Continuous	Primary	FAS	ANCOVA	RD-MI	J2R-MI<br>S1-SI<br>S2-SI<br>TP-MI<br>MMRM
				Secondary	FAS	MMRM	-	-
Primary	5% responders	2	Binary	Primary	FAS	LR	RD-MI	J2R-MI<br>S1-SI<br>S2-SI<br>TP-MI<br>MMRM<br>Non-responder
				Secondary	FAS	LR	MMRM	-
**Confirmatory secondary endpoints**								
Primary	10% responders	3	Binary	Primary	FAS	LR	RD-MI	Non-responders
				Secondary	FAS	LR	MMRM	-
Primary	15% responders	4	Binary	Primary	FAS	LR	RD-MI	Non-responders
				Secondary	FAS	LR	MMRM	-
Primary	WC change (cm)	5	Continuous	Primary	FAS	ANCOVA	RD-MI	J2R-MI
				Secondary	FAS	MMRM	-	-
Secondary	sBP change (mmHg)	6	Continuous	Primary	FAS	ANCOVA	RD-MI	J2R-MI

FAS = full analysis set; ANCOVA = analysis of covariance; RD-MI = multiple imputation using retrieved subjects; J2R-MI = jump to reference multiple imputation; S1-SI and S2-SI = single imputation as done by Sacks; TP-MI = tipping point multiple imputation; MMRM = mixed model for repeated measurements; LR = logistic regression; WC = waist circumference; sBP = systolic blood pressure

10.3.2.2 Supportive secondary endpoints

Supportive secondary endpoints are listed in section 4. All tests are tests of superiority of semaglutide 2.4 mg to semaglutide placebo.

Analyses addressing the primary estimand

The effect-related supportive secondary endpoints will be analysed using the same imputation approach as used for the primary endpoints and to address the primary estimand. The imputation model is the same as for the primary endpoints with body weight replaced by assessments of the endpoint to be analysed. The statistical model for continuous endpoints will be ANCOVA with factor and covariate as for the primary endpoint % weight change with baseline body weight replaced by the baseline assessment of the endpoint to be analysed.
For lipids and hsCRP a multiplicative model will be used, i.e. the ratio between post randomisation measurements and baseline will be calculated instead of differences, and both the dependent variable and covariate will be log-transformed.

Analyses addressing the secondary estimand

The supportive secondary endpoints which relate to the primary objective will be analysed to address the secondary estimand using the same MMRM for efficacy described for the primary endpoints.

Additional considerations for statistical analyses

Supportive secondary endpoints evaluating the one-year effect of semaglutide 2.4 mg will be analysed using the same imputation approach as used for the primary endpoints and to address the primary estimand. The imputation model is the same as for the primary endpoints. The statistical model for continuous endpoints will be ANCOVA with factor and covariate as for the primary endpoint % weight change. The statistical model for body weight responder endpoints will be logistic regression with factor and covariate as for the primary endpoint 5% responders.

The supportive secondary endpoint “Change from baseline to week 52 in body weight (%)” will be compared to the primary endpoint “Change from baseline to week 104 in body weight (%)”. The comparison will be done using the primary estimand at week 52 and week 104.

Sensitivity analyses for supportive secondary endpoints

For supportive secondary endpoints no sensitivity analysis will be carried out.

Analysis of safety endpoints

The safety endpoint pulse will be analysed using an MMRM for efficacy as described in section 10.3.1. For amylase, lipase and calcitonin descriptive statistics will be provided. The analysis of calcitonin will be stratified by gender.

Adverse events will be defined as “treatment-emergent” (TEAE), if the onset of the event occurs in the on-treatment period (see definition in section 10.2). TEAEs and SAEs will be summarised by descriptive statistics, such as frequencies and rates. No formal statistical inference will be carried out based on the number of TEAEs and SAEs.

An overview of all analysis and imputation methods to address the effectiveness and efficacy estimands for supportive secondary endpoints is given in Table 10-4.

Table 10-4 Analysis and imputation methods to address the effectiveness and efficacy estimands for supportive secondary endpoints

Objective	Endpoint	Endpoint type	Estimand	Analysis set	Statistical model	Imputation approach	Sensitivity analyses
**Supportive secondary endpoints (effect related)**							
From baseline to week 104							
Primary	Weight change (kg)	Continuous	Primary	FAS	ANCOVA	RD-MI	-
			Secondary	FAS	MMRM	-	-
Primary	BMI change (kg/m^2)	Continuous	Primary	FAS	ANCOVA	RD-MI	-
			Secondary	FAS	MMRM	-	-
Secondary	HbA_{1c} change (%, mmol/mol)	Continuous	Primary	FAS	ANCOVA	RD-MI	-
Secondary	FPG change (mg/dL)	Continuous	Primary	FAS	ANCOVA	RD-MI	-
Secondary	Fasting serum insulin change (mIU/L)	Continuous	Primary	FAS	ANCOVA	RD-MI	-
Secondary	dBP change (mmHg)	Continuous	Primary	FAS	ANCOVA	RD-MI	-
Secondary	Total cholesterol change (mg/dL)	Continuous	Primary	FAS	ANCOVA	RD-MI	-
Secondary	HDL change (mg/dL)	Continuous	Primary	FAS	ANCOVA	RD-MI	-
Secondary	LDL change (mg/dL)	Continuous	Primary	FAS	ANCOVA	RD-MI	-
Secondary	VLDL change (mg/dL)	Continuous	Primary	FAS	ANCOVA	RD-MI	-
Secondary	FFA change (mg/dL)	Continuous	Primary	FAS	ANCOVA	RD-MI	-
Secondary	Triglycerides change (mg/dL)	Continuous	Primary	FAS	ANCOVA	RD-MI	-
Secondary	hsCRP change (mg/L)	Continuous	Primary	FAS	ANCOVA	RD-MI	-
From baseline to week 52							
Secondary	Weight change (%, kg)	Continuous	Primary	FAS	ANCOVA	RD-MI	-
Secondary	BMI change (kg/m^2)	Continuous	Primary	FAS	ANCOVA	RD-MI	-
Secondary	WC change (cm)	Continuous	Primary	FAS	ANCOVA	RD-MI	-
Secondary	5% responders	Binary	Primary	FAS	LR	RD-MI	-
Secondary	10% responders	Binary	Primary	FAS	LR	RD-MI	-
Secondary	15% responders	Binary	Primary	FAS	LR	RD-MI	-
**Supportive secondary endpoints (safety related)**							
Secondary	Number of TEAEs	Continuous	-	SAS	-	-	-
Secondary	Number of SAEs	Continuous	-	SAS	-	-	-
Secondary	Pulse change (bpm)	Continuous	-	SAS	MMRM	-	-
Secondary	Amylase change (U/L)	Continuous	-	SAS	Descriptive statistics	-	-
Secondary	Lipase change (U/L)	Continuous	-	SAS	Descriptive statistics	-	-
Secondary	Calcitonin change (ng/L)	Continuous	-	SAS	Descriptive statistics	-	-

FAS = full analysis set; ANCOVA = analysis of covariance; RD-MI = multiple imputation using retrieved subjects; MMRM = mixed model for repeated measurements; BMI = body mass index; HbA_{1c} = Hemoglobin A1c; FPG = fasting plasma glucose; dBP = diastolic blood pressure; HDL = high density lipoprotein; LDL = low density lipoprotein; VLDL = very low density lipoprotein; FFA = free fatty acids; hsCRP = high sensitivity C-Reactive Protein; WC = waist circumference; LR = logistic regression; TEAEs = treatment emergent adverse events; SAEs = serious adverse events

10.3.3 Exploratory endpoint

Exploratory endpoints are listed in section 4. Observed data for exploratory endpoints will be summarised by descriptive statistics.

Food cravings will be assessed by the CoEQ at baseline (week 0), week 20, week 52 and week 104, with each of the 19 items rated on a 10 points scale. The rating for each question will be summarised using descriptive statistics (applicable for US and Canada only).

10.3.4 Explorative statistical analysis for pharmacogenetics and biomarkers

The statistical analysis of biomarker endpoints is described under section 10.3.2.2.

10.3.5 Other analyses

All collected data that were not defined as endpoints will be summarised by descriptive statistics.

10.4 Pharmacokinetic and/or pharmacodynamic modelling

Not applicable for this trial.

11 Appendices

Appendix 1 Abbreviations and Trademarks

AD	available but discontinued
AE	adverse event
ALT	alanine aminotransferase
ANCOVA	analysis of covariance
AST	aspartate aminotransferase
AT	available on randomised treatment
BMI	body mass index
CLAE	clinical laboratory adverse event
CoEQ	control of eating questionnaire
CRF	case report form
DFU	direction for use
DUN	dispensing unit number
EAC	event adjudication committee
EAS	event adjudication system
ECG	electrocardiogram
eGFR	estimated glomerular filtration rate
FAS	full analysis set
FDA	U.S. Food and Drug Administration
FDAAA	FDA Amendments Act
FPG	fasting plasma glucose
FSH	follicle-stimulating hormone
GCP	Good Clinical Practice
GLP-1 RA	glucagon-like peptide-1 receptor agonist
HbA_{1c}	glycated haemoglobin
HRT	hormone replacement therapy
hsCRP	high sensitive C-reactive protein
IB	investigator’s brochure
ICH	International Council for Harmonisation
IEC	independent ethics committee
IMP	investigational medicinal product

IRB	institutional review board
IWRS	interactive web response system
KDIGO	kidney disease improving global outcome
LAO	last available observation
LCD	low-calorie diet
LDL	low-density lipoprotein
LPLV	last patient last visit
LR	logistic regression
MEN2	multiple endocrine neoplasia type 2
MD	missing and discontinued
MMRM	mixed model for repeated measurements
MT	missing on randomised treatment
MTC	medullary thyroid cancer
OAD	oral antidiabetic drug
OR	odds ratio
PAI-1	plasminogen activator inhibitor-1
PCD	primary completion date
RD-MI	multiple imputation using retrieved subjects
SAS	safety analysis set
SAE	serious adverse event
SAP	statistical analysis plan
sBP	systolic blood pressure
s.c.	subcutaneus
SD	standard deviation
SUSAR	suspected unexpected serious adverse reactions
T2D	type 2 diabetes
TEAE	treatment emergent adverse event
TEE	total energy expenditure
TMM	trial materials manual
TSH	thyroid stimulating hormone
WC	waist circumference

WOCBP	woman of child bearing potential

Appendix 2 Clinical laboratory tests

- The tests detailed in Table 11-1 and Table 11-2 will be performed by the central laboratory.
- Laboratory samples specified in the protocol should be sent to the central laboratory for analysis.
- Additional tests may be performed at local laboratory any time during the trial as determined necessary by the investigator or required by local regulations.
- The laboratory equipment may provide analyses not requested in the protocol but produced automatically in connection with the requested analyses according to specifications in the laboratory standard operating procedures. Such data will not be transferred to the trial database, but abnormal values will be reported to the investigator.
- The investigator must review all laboratory results for concomitant illnesses and AEs.
- Laboratory samples will be destroyed no later than at finalisation of the clinical trial report.
- For haematology samples (differential count) where the test result is not normal, then a part of the sample may be kept for up to two years or according to local regulations.

Table 11-1 Protocol-required efficacy laboratory assessments

Laboratory assessments	Parameters
Glucose metabolism	• Fasting plasma glucose[1]<br>• HbA_{1c}<br>• Fasting serum insulin
Lipids	• Cholesterol<br>• High density lipoprotein (HDL) cholesterol<br>• Low density lipoprotein (LDL) cholesterol<br>• Triglycerides<br>• Very-low-density lipoprotein (VLDL) cholesterol<br>• Free fatty acids
Biomarkers	• Serum hsCRP
NOTES : [1] A FPG result ≤ 3.9 mmol/L (70 mg/dL) in relation to planned fasting visits should not be reported as a hypoglycaemic episode but as a CLAE at the discretion of the investigator Appendix 4	

Table 11-2 Protocol-required safety laboratory assessments

Laboratory assessments	Parameters
Haematology	• Basophils<br>• Eosinophils<br>• Erythrocytes<br>• Haematocrit<br>• Haemoglobin<br>• Leucocytes<br>• Lymphocytes<br>• Monocytes<br>• Neutrophils<br>• Thrombocytes
Biochemistry[1]	• Alanine Aminotransferase (ALT)[2]<br>• Albumine<br>• Albumine corrected calcium<br>• Alkaline phosphatase<br>• Amylase[3]<br>• Aspartate Aminotransferase (AST)[2]<br>• Calcitonin[3]<br>• Creatine kinase<br>• Creatinine<br>• Lipase[3]<br>• Potassium<br>• Sodium<br>• Thyroid stimulating hormone (TSH)[4]<br>• Total bilirubin<br>• Urea
Pregnancy Testing	• Urine human chorionic gonadotropin (hCG) pregnancy test (as needed for women of childbearing potential[5]
Other tests	• eGFR calculated by the central laboratory based on the creatinine value using the CKD EPI equation as defined by KDIGO 2012[67]<br>• (Tryptase in case of severe hypersensitivity, see Section 9.9)
Notes :<br>[1]Details of required actions and follow-up assessments for increased liver parameters including any discontinuation criteria are given in Appendix 4 (Hy's Law) and Section 8.1.<br>[2]If ALT or AST >3 upper normal limit (UNL), additional blood samples should be taken from the subject to analyse international normalised ratio (INR) by central laboratory (except at screening visit). Repeat testing of the abnormal lab assessments should be performed for the subject until abnormalities return to normal or baseline state.<br>[3]Not collected at week 84.<br>[4]If TSH level is out of normal range, additional testing will be performed by central lab: total and free T3 and T4 except at screening visit.<br>[5]Local urine testing will be standard unless serum testing is required by local regulation or IRB/IEC.	

- Laboratory outlier: If the following laboratory parameters are above/below the cut-off-values in Table 11-3 they are considered to be laboratory outliers and should be reported by completing a laboratory outlier form in the CRF:

Table 11-3 Criteria for laboratory outliers

	Cut-off
**Haematology**	
Leucocytes	$< 1 \times 10^9$/L
Lymphocytes	$< 0.2 \times 10^9$/L
Thrombocytes	$< 25 \times 10^9$/L
**Biochemistry**	
Albumin corrected calcium	< 1.50 mmol/L or > 3.4 mmol/L
Alkaline phosphatase	> 20 × ULN
Calcitonin	> 100 ng/L
Creatinine	> 6 × ULN
Creatine kinase	> 10 × ULN
Potassium	< 2.50 mmol/L or > 7.0 mmol/L
Sodium	< 120 mmol/L or > 160 mmol/L

Hepatic laboratory outlier: if the following hepatic laboratory parameters are above the cut-offs values in Table 11-4, it is considered to be a hepatic laboratory outlier and should be reported by completing a hepatic event form in the CRF. It is at the investigator's discretion to determine whether it should also be reported as an adverse event (Appendix 4).

Table 11-4 Criteria for hepatic laboratory outliers

	Cut-off
Alkaline phosphatase	>20 x UNL
ALT	>5 x UNL
AST	>5 x UNL
Total bilirubin	>10 x UNL

Please note that in case of a hepatic event defined as ALT or AST >3 x UNL and total bilirubin > 2 x UNL, where no alternative aetiology exists (Hy's law), this must be reported as an SAE using the important medical event criterion if no other seriousness criteria are applicable

Appendix 3 Trial governance considerations

1) Regulatory and ethical considerations

- This trial will be conducted in accordance with the protocol and with the following:
    1. Consensus ethical principles derived from international guidelines including the Declaration of Helsinki [76] and applicable ICH Good Clinical Practice (GCP) Guideline[77]
    2. Applicable laws and regulations
- The protocol, informed consent form, IB (as applicable) and other relevant documents (e.g. advertisements), must be submitted to an IRB/IEC and reviewed and approved by the IRB/IEC before the trial is initiated.
- Regulatory authorities will receive the clinical trial application, protocol amendments, reports on SAEs, and the clinical trial report according to national requirements.
- Any amendments to the protocol will require IRB/IEC approval before implementation of changes made to the trial design, except for changes necessary to eliminate an immediate safety hazard to trial subjects.
- Before a trial site is allowed to start screening subjects, written notification from Novo Nordisk must be received.
- The investigator will be responsible for:
    1. providing written summaries of the status of the trial annually or more frequently in accordance with the requirements, policies, and procedures established by the IRB/IEC and/or regulatory authorities
    2. notifying the IRB/IEC of SAEs or other significant safety findings as required by IRB/IEC procedures
    3. providing oversight of the conduct of the trial at the site and adherence to requirements of ICH guidelines, the IRB/IEC, and all other applicable local regulations
    4. ensuring submission of the clinical trial report synopsis to the IRB/IEC.

2) Financial disclosure

Investigators and subinvestigators will provide Novo Nordisk with sufficient, accurate financial information as requested to allow Novo Nordisk to submit complete and accurate financial certification or disclosure statements to the appropriate regulatory authorities. Investigators are responsible for providing information on financial interests during the course of the trial and one year after completion of the trial.
For US trial sites: verification under disclosures per Code of Federal Regulations (CFR) of Financial Conflict of Interest.

3) Informed consent process

- The investigator or his/her representative will explain the nature of the trial to the subject and answer all questions regarding the trial.
- The investigator must ensure the subject ample time to come to a decision whether or not to participate in the trial.
- Subjects must be informed that their participation is voluntary.
- Subjects will be required to sign and date a statement of informed consent that meets the requirements of local regulations, ICH guidelines [77], Declaration of Helsinki [76] and the IRB/IEC or trial site.
- The medical record must include a statement that written informed consent was obtained before any trial related activity and the date when the written consent was obtained. The authorised person obtaining the informed consent must also sign and date the informed consent form before any trial related activity.
- The responsibility of seeking informed consent must remain with the investigator, but the investigator may delegate the task of informing to a medically qualified person, in accordance with local requirements.
- Subjects must be re-consented to the most current version of the informed consent form(s) during their participation in the trial.
- A copy of the informed consent form(s) must be provided to the subject.

4) Information to subjects during trial

The site will be offered a communication package for the subject during the conduct of the trial. The package content is issued by Novo Nordisk. The communication package will contain written information intended for distribution to the subjects. The written information will be translated and adjusted to local requirements and distributed to the subject at the discretion of the investigator.

All written information to subjects must be sent to IRB/IEC for approval/favourable opinion and to regulatory authorities for approval or notification according to local regulations.

Different initiatives for subject retention will be implemented throughout this trial. Site retention activities may include cooking classes, group meetings, and others. Materials and items will be supplied if locally acceptable. The retention items will be relevant for the subjects' participation in the trial and/or their obesity and will not exceed local fair market value.

The initiatives for subjects must be sent to IRB/IEC for approval/favourable opinion and to regulatory authorities for approval or notification according to local regulations.

5) Data protection

- Subjects will be assigned a 6-digit unique identifier, a subject number. Any subject records or datasets that are transferred to Novo Nordisk will contain the identifier only; subject names or any information which would make the subject identifiable will not be transferred.
- The subject and any biological material obtained from the subject will be identified by subject number, visit number and trial ID. Appropriate measures such as encryption or leaving out certain identifiers will be enforced to protect the identity of subjects as required by local, regional and national requirements.
- The subject must be informed that his/her personal trial related data will be used by Novo Nordisk in accordance with local data protection law. The disclosure of the data must also be explained to the subject.
- The subject must be informed that his/her medical records may be examined by auditors or other authorised personnel appointed by Novo Nordisk, by appropriate IRB/IEC members, and by inspectors from regulatory authorities.

6) Committee structure

Novo Nordisk safety committee

Novo Nordisk will constitute an internal Semaglutide s.c. safety committee to perform ongoing safety surveillance. The Semaglutide s.c. safety committee may recommend unblinding of any data for further analysis, and in this case an independent ad hoc group will be established in order to maintain the blinding of the trial personnel.

Event adjudication committee

An independent external EAC is established to perform ongoing blinded adjudication of selected types of events and deaths (see Table 9-1and Appendix 4). The EAC will evaluate events sent for adjudication using pre-defined definitions and guidelines in accordance with the EAC Charter. The evaluation is based on review of pre-defined clinical data collected by the trial sites.

The EAC is composed of permanent members covering all required medical specialities. EAC members must disclose any potential conflicts of interest and must be independent of Novo Nordisk. The EAC will have no authorisations to impact on trial conduct, trial protocol or amendments.

The assessment made by both the EAC and the investigator will be presented in the clinical trial report.

In this trial, cardiovascular events will be adjudicated in order to adequately characterize the cardiovascular safety profile, since cardiovascular disease is an important and serious comorbidity of obesity[78]. In addition, events of acute pancreatitis will be adjudicated because Novo Nordisk

monitors these events closely as treatment with GLP-1 RAs has been associated with acute pancreatitis.

7) Publication policy

The information obtained during the conduct of this trial is considered confidential, and may be used by or on behalf of Novo Nordisk for regulatory purposes as well as for the general development of the trial product. All information supplied by Novo Nordisk in connection with this trial shall remain the sole property of Novo Nordisk and is to be considered confidential information.

No confidential information shall be disclosed to others without prior written consent from Novo Nordisk. Such information shall not be used except in the performance of this trial.
The information obtained during this trial may be made available to other investigators who are conducting other clinical trials with the trial product, if deemed necessary by Novo Nordisk.
Provided that certain conditions are fulfilled, Novo Nordisk may grant access to information obtained during this trial to researchers who require access for research projects studying the same disease and/or trial product studied in this trial.

Novo Nordisk may publish on its clinical trials website a redacted clinical trial report for this trial.

One (or two) investigator(s) will be appointed by Novo Nordisk to review and sign the clinical trial report (signatory investigator) on behalf of all participating investigators. The signatory investigator(s) will be appointed based upon the criteria defined by the International Committee of Medical Journal Editors for research publications[79] .

Communication of results

Novo Nordisk commits to communicate and disclose results of trials regardless of outcome. Disclosure includes publication of a manuscript in a peer-reviewed scientific journal, abstract submission with a poster or oral presentation at a scientific meeting or disclosure by other means.

The results of this trial will be subject to public disclosure on external web sites according to international and national regulations. Novo Nordisk reserves the right to defer the release of data until specified milestones are reached, for example when the clinical trial report is available. This includes the right not to release the results of interim analyses, because the release of such information may influence the results of the entire trial.

At the end of the trial, one or more scientific publications may be prepared collaboratively by the investigator(s) and Novo Nordisk. Novo Nordisk reserves the right to postpone publication and/or communication for up to 60 days to protect intellectual property.

In all cases the trial results will be reported in an objective, accurate, balanced and complete manner, with a discussion of the strengths and limitations. In the event of any disagreement on the

content of any publication, both the investigators' and Novo Nordisk opinions will be fairly and sufficiently represented in the publication.

Authorship

Novo Nordisk will work with one or more investigator(s) and other experts who have contributed to the trial concept or design, acquisition, analysis or interpretation of data to report the results in one or more publications.

Authorship of publications should be in accordance with the Recommendations for the Conduct, Reporting, Editing and Publication of Scholarly Work in Medical Journals by the International Committee of Medical Journal Editors[79].

All authors will be provided with the relevant statistical tables, figures, and reports needed to evaluate the planned publication.

Where required by the journal, the investigator from each trial site will be named in an acknowledgement or in the supplementary material, as specified by the journal.

Site-specific publication(s) by investigator(s)

For a multicentre clinical trial, analyses based on single-site data usually have significant statistical limitations and frequently do not provide meaningful information for healthcare professionals or subjects, and therefore may not be supported by Novo Nordisk. Thus, Novo Nordisk may deny a request or ask for deferment of the publication of individual site results until the primary manuscript is accepted for publication. In line with Good Publication Practice, such individual reports should not precede the primary manuscript and should always reference the primary manuscript of the trial.

Investigator access to data and review of results

As owner of the trial database, Novo Nordisk has the discretion to determine who will have access to the database.
Individual investigators will have their own subjects' data, and will be provided with the randomisation code after results are available.

8) Dissemination of clinical trial data

Information of the trial will be disclosed at clinicaltrials.gov and novonordisk-trials.com. It will also be disclosed according to other applicable requirements such as those of the International Committee of Medical Journal Editors (ICMJE)[80], the Food and Drug Administration Amendment Act (FDAAA)[81], European Commission Requirements[1, 82] and other relevant recommendations or regulations. If a subject requests to be included in the trial via the Novo Nordisk e-mail contact at these web sites, Novo Nordisk may disclose the investigator's contact details to the subject. As a result of increasing requirements for transparency, some countries require public disclosure of investigator names and their affiliations.

The Primary Completion Date (PCD) is the last assessment of the primary endpoint, and is for this trial Last Subject First Treatment (LSFT) + 104 weeks corresponding to V33 ('end of treatment' visit). If the last subject is withdrawn early, the PCD is considered the date when the last subject would have completed V33 ('end of treatment' visit). The PCD determines the deadline for results disclosure at clinicaltrials.gov according to FDAAA.

9) Data quality assurance

Case Report Forms (CRFs)

- Novo Nordisk or designee is responsible for the data management of this trial including quality checking of the data.
- All subject data relating to the trial will be recorded on electronic CRFs unless transmitted electronically to Novo Nordisk or designee (e.g. laboratory data). The investigator is responsible for verifying that data entries are accurate and correct by physically or electronically signing the CRF.
- For some data both electronic and paper CRFs are used.
- The following will be provided as paper CRFs:
    1. Pregnancy forms

- The following will be provided as paper CRFs to be used when access to the electronic CRF is revoked or temporarily unavailable:
    1. AE forms
    2. Safety information forms
    3. Technical complaint forms (also to be used to report complaints that are not subject related, e.g. discovered at trial site before allocation)

- Corrections to the CRF data may be made by the investigator or the investigator's delegated staff. An audit trail will be maintained in the CRF application containing as a minimum: the old and the new data, identification of the person entering the data, date and time of the entry and reason for the correction. If corrections are made by the investigator's delegated staff after the date when the investigator signed the CRF, the CRF must be signed and dated again by the investigator.
- The investigator must ensure that data is recorded in the CRF as soon as possible, preferably within 5 working days after the visit. Once data has been entered, it will be available to Novo Nordisk for data verification and validation purposes.

Monitoring

- The investigator must permit trial-related monitoring, audits, IRB/IEC review, and regulatory agency inspections and provide direct access to source data documents (original documents, data and records). Direct access includes permission to examine, analyse, verify and reproduce

any record(s) and report(s) that are important to the evaluation of the trial. If the electronic medical record does not have a visible audit trail, the investigator must provide the monitor with signed and dated printouts. In addition the relevant trial site staff should be available for discussions at monitoring visits and between monitoring visits (e.g. by telephone).

- Trial monitors will perform ongoing source data verification to confirm that data entered into the CRF by authorised site personnel are accurate, complete and verifiable from source documents; that the safety and rights of subjects are being protected, to monitor drug accountability and collect completed paper CRF pages, if applicable, and that the trial is being conducted in accordance with the currently approved protocol and any other trial agreements, ICH GCP, and all applicable regulatory requirements.

- Monitoring will be conducted using a risk based approach including risk assessment, monitoring plans, centralised monitoring (remote assessment of data by Novo Nordisk) and visits to trial sites.

- Monitors will review the subject's medical records and other source data e.g. the diaries and mental health assessment instruments, to ensure consistency and/or identify omissions compared to the CRF.

Protocol compliance

Deviations from the protocol should be avoided. If deviations do occur, the investigator must inform the monitor without delay and the implications of the deviation must be reviewed and discussed.

Deviations must be documented and explained in a protocol deviation by stating the reason, date, and the action(s) taken. Some deviations, for which corrections are not possible, can be acknowledged and confirmed via edit checks in the CRF or via listings from the trial database.

10) Source documents

- All data entered in the CRF must be verifiable in source documentation other than the CRF.
- The original of the completed diaries must not be removed from the trial site, unless they form part of the CRF and a copy is kept at the site. For food and physical activity diary, if the subject uses an app or a tool other than the paper diaries, the medical record or dietician's notes from the diet and physical activity counselling can be used as source document.
- Source documents provide evidence for the existence of the subject and substantiate the integrity of the data collected. Source documents are filed at the trial site.
- Data reported in the CRF that are transcribed from source documents must be consistent with the source documents or the discrepancies must be explained. The investigator may need to

request previous medical records or transfer records. Also, current medical records must be available.

- It must be possible to verify subject's medical history in the source documents such as subject's medical record.
- Subjects completing electronic patient reported outcome instruments are the data originators. Data will be transmitted to a technology service provider database, thus the service provider database is the source.
- The investigator must document any attempt to obtain external medical information by noting the date(s) when information was requested and who was contacted.
- Definition of what constitutes source data can be found in a source document agreement at each trial site. There will only be one source document defined at any time for any data element.

11) Retention of clinical trial documentation

- Records and documents, including signed informed consent forms, pertaining to the conduct of this trial must be retained by the investigator for 15 years after end of trial unless local regulations or institutional policies require a longer retention period. No records may be destroyed during the retention period without the written approval of Novo Nordisk. No records may be transferred to another location or party without written notification to Novo Nordisk.
- The investigator must be able to access his/her trial documents without involving Novo Nordisk in any way. If applicable, electronic CRF and other subject data will be provided in an electronic readable format to the investigator before access is revoked to the systems and/or electronic devices supplied by Novo Nordisk. Site-specific CRFs and other subject data (in an electronic readable format or as paper copies or prints) must be retained by the trial site. If the provided electronic data (e.g. the CD-ROM) is not readable during the entire storage period, the investigator can request a new copy. A copy of all data will be stored by Novo Nordisk.
- Subject's medical records must be kept for the maximum period permitted by the hospital, institution or private practice

12) Trial and site closure

Novo Nordisk reserves the right to close the trial site or terminate the trial at any time for any reason at the sole discretion of Novo Nordisk. If the trial is suspended or terminated, the investigator must inform the subjects promptly and ensure appropriate therapy and follow-up. The investigator and/or Novo Nordisk must also promptly inform the regulatory authorities and IRBs/IECs and provide a detailed written explanation.

Trial sites will be closed upon trial completion. A trial site is considered closed when all required documents and trial supplies have been collected and a trial site closure visit has been performed.

The investigator may initiate trial site closure at any time, provided there is reasonable cause and sufficient notice is given in advance of the intended termination.

Reasons for the early closure of a trial site by Novo Nordisk or investigator may include but are not limited to:

- failure of the investigator to comply with the protocol, the requirements of the IRB/IEC or local health authorities, Novo Nordisk procedures or GCP guidelines
- inadequate recruitment of subjects by the investigator
- discontinuation of further trial product development.

13) Responsibilities

The investigator is accountable for the conduct of the trial at his/her site and must ensure adequate supervision of the conduct of the trial at the trial site. If any tasks are delegated, the investigator must maintain a log of appropriately qualified persons to whom he/she has delegated specified trial-related duties. The investigator must ensure that there is adequate and documented training for all staff participating in the conduct of the trial. It is the investigator's responsibility to supervise the conduct of the trial and to protect the rights, safety, and well-being of the subjects.
A qualified physician, who is an investigator or a sub-investigator for the trial, must be responsible for all trial-related medical decisions.

The investigator is responsible for filing essential documents (i.e. those documents which individually and collectively permit evaluation of the conduct of a trial and the quality of the data produced) in the investigator trial master file. The documents, including the subject identification code list must be kept in a secure locked facility so that no unauthorized persons can get access to the data.

The investigator will take all necessary technical and organisational safety measures to prevent accidental or wrongful destruction, loss or deterioration of data. The investigator will prevent any unauthorised access to data or any other processing of data against applicable law. The investigator must be able to provide the necessary information or otherwise demonstrate to Novo Nordisk that such technical and organisational safety measures have been taken.

During any period of unavailability, the investigator must delegate responsibility for medical care of subjects to a specific qualified physician who will be readily available to subjects during that time.

If the investigator is no longer able to fulfil the role as investigator (e.g. if he/she moves or retires) a new investigator will be appointed in consultation with Novo Nordisk.
The investigator and other site personnel must have sufficient English skills according to their assigned task(s).

14) Indemnity statement

Novo Nordisk carries product liability for its products, and liability as assumed under the special laws, acts and/or guidelines for conducting clinical trials in any country, unless others have shown negligence.

Novo Nordisk assumes no liability in the event of negligence or any other liability of the sites or investigators conducting the trial or by persons for whom the site or investigator are responsible.

Novo Nordisk may pay additional costs incurred in relation to assessments relevant for following the safety of the subject. Investigator must contact Novo Nordisk on a case by case basis for whether the costs will be covered.

Appendix 4 Adverse events: definitions and procedures for recording, evaluation, follow-up, and reporting

AE definition
• An AE is any untoward medical occurrence in a clinical trial subject that is temporally associated with the use of a medicinal product, whether or not considered related to the medicinal product.<br>• An AE can be any unfavourable and unintended sign, including an abnormal laboratory finding, symptom or disease (new or exacerbated) temporally associated with the use of a medicinal product.

**Events meeting the AE definition**
• Any abnormal laboratory test results or safety assessments, including those that worsen from baseline, considered clinically significant in the medical and scientific judgment of the investigator.<br>• A CLAE: a clinical abnormal laboratory finding which is clinically significant, i.e. an abnormality that suggests a disease and/or organ toxicity and is of a severity that requires active management. Active management includes active treatment or further investigations, for example change of medicine dose or more frequent follow-up due to the abnormality.<br>• Exacerbation of a chronic or intermittent pre-existing condition including either an increase in frequency and/or intensity of the condition.<br>• Signs, symptoms or the clinical sequelae of a suspected drug-drug interaction.<br>• Signs, symptoms or the clinical sequelae of a suspected overdose of trial product regardless of intent.<br>• A "lack of efficacy" or "failure of expected pharmacological action" per se will not be reported as an AE or SAE. Such instances will be captured in the efficacy assessments. However, the signs, symptoms and/or clinical sequelae resulting from lack of efficacy will be reported as AE or SAE if they fulfil the definition.
**Events NOT meeting the AE definition**
• Pre-existing conditions, anticipated day-to-day fluctuations of pre-existing conditions, including those identified during screening or other trial procedures performed before exposure to trial product.<br>Note: pre-existing conditions should be recorded as medical history/concomitant illness.<br>• Pre-planned procedures, unless the condition for which the procedure was planned has worsened from the first trial related activity after the subject has signed the informed consent.

**Definition of an SAE**
**An SAE is an AE that fulfils at least one of the following criteria:**
• **Results in death**
• **Is life-threatening**<br>The term 'life-threatening' in the definition of 'serious' refers to an event in which the subject was at risk of death at the time of the event. It does not refer to an event which hypothetically might have caused death, if it were more severe.
• **Requires inpatient hospitalisation or prolongation of existing hospitalisation**<br>• Hospitalisation signifies that the subject has been detained at the hospital or emergency ward for observation and/or treatment that would not have been appropriate in the physician's office or outpatient setting. Complications that occur during hospitalisation are AEs. If a complication prolongs hospitalisation or fulfils any other serious criteria, the event is serious. When in doubt as to whether "hospitalisation" occurred or was necessary, the AE should be considered serious.<br>• Hospitalisation for elective treatment of a pre-existing condition that did not worsen from baseline is not considered an AE.<br>Note:<br>▪ Hospitalisations for administrative, trial related and social purposes do not constitute AEs and should therefore

not be reported as AEs or SAEs.<br>▪ Hospital admissions for surgical procedures, planned before trial inclusion, are not considered AEs or SAEs.
• **Results in persistent disability/incapacity**<br>• The term disability means a substantial disruption of a person's ability to conduct normal life functions.<br>• This definition is not intended to include experience of relatively minor medical significance such as uncomplicated headache, nausea, vomiting, diarrhoea, influenza, and accidental trauma (e.g. sprained ankle), which may interfere with or prevent everyday life functions but do not constitute a substantial disruption.
• **Is a congenital anomaly/birth defect**
• **Important medical event:**<br>• Medical or scientific judgment should be exercised in deciding whether SAE reporting is appropriate in other situations. This includes important medical events that may not be immediately life-threatening or result in death or hospitalisation, but may jeopardise the subject or may require medical or surgical intervention to prevent one of the other outcomes listed in the above definition. These events should usually be considered serious and reported as SAEs using the important medical event criterion.<br>• The following AEs must always be reported as SAEs using the important medical event criterion, if no other seriousness criteria are applicable:<br>▪ suspicion of transmission of infectious agents via the trial product.<br>▪ risk of liver injury defined as ALT or AST >3 x UNL and total bilirubin >2 x UNL, where no alternative aetiology exists (Hy's law).

Description of AEs requiring additional data collection (via specific event form) and events for adjudication.

AEs requiring additional data collection

AEs requiring additional data collection are AEs where the additional data will benefit the evaluation of the safety of the trial product (see Table 9-1). The selection of these events is based on the non-clinical and clinical data with semaglutide, knowledge from the GLP-1 RA drug class as well as regulatory requirements.

Event type	Description
Acute gallbladder disease	Events of symptomatic acute gallbladder disease (including gallstones and, cholecystitis)
Acute pancreatitis	The diagnosis of acute pancreatitis requires two of the following three features:<br>(1) abdominal pain consistent with acute pancreatitis (acute onset of a persistent, severe, epigastric pain often radiating to the back)<br>(2) serum lipase activity (and/or amylase activity) at least three times greater than the upper limit of normal<br>(3) characteristic findings of acute pancreatitis on imaging.
Malignant neoplasm	Malignant neoplasm by histopathology or other substantial clinical evidence
Hepatic event	Hepatic event defined as:<br>– Disorders of the liver including cholestatic conditions and liver related signs and symptoms<br>– ALT or AST > 3x UNL and total bilirubin > 2x UNL or INR > 1.5x*<br>– ALT or AST > 3x UNL with the appearance of fatigue, nausea, vomiting, right upper quadrant pain or tenderness, fever, rash, and/or eosinophilia (>5%).<br>*Please note that in case of a hepatic event defined as ALT or AST > 3x

	UNL and total<br>bilirubin > 2 x UNL, where no alternative aetiology exists (Hy's law), this must be reported as an SAE using the important medical event criterion if no other seriousness criteria are applicable.
Medication error:	A medication error concerning trial products is defined as:<br>• Administration of wrong drug.<br>• Note: Use of wrong dispensing unit number (DUN) is not considered a medication error unless it results in a confirmed administration of wrong drug.<br>• Wrong route of administration, such as intramuscular instead of subcutaneous.<br>• Accidental administration of more than 2.4 mg/week or a higher dose than intended during dose escalation, however, the administered dose must deviate from the intended dose to an extent where clinical consequences for the trial subject were likely to happen as judged by the investigator, although they did not necessarily occur.
Misuse or abuse of trial product*	• Misuse is when the trial product is intentionally and inappropriately used. Abuse of trial product is persistent or sporadic, intentional excessive use, which is accompanied by harmful physical or psychological effects (e.g. overdose with the intention to cause harm).

*Additional data for Misuse or abuse of trial product is reported on the medication error event form.

Events for adjudication

**Event type**	**Description**	**Adjudication outcome**
Death	All-cause death	• Cardiovascular death (including undetermined cause of death)<br>• Non-Cardiovascular death
Acute Coronary Syndrome	Acute Coronary Syndrome conditions include all types of acute myocardial infarction and hospitalisation for unstable angina pectoris	• Acute myocardial infarction (including subgroup classifications)<br>• Hospitalisation for unstable angina pectoris
Cerebrovascular events	Episode of focal or global neurological dysfunction that could be caused by brain, spinal cord, or retinal vascular injury as a result of haemorrhage or infarction	• Ischaemic stroke<br>• Haemorrhagic stroke<br>• Undetermined stroke<br>• Transient Ischaemic Attack
Coronary artery revascularisation	Coronary revascularisation procedure is a catheter-based (PCI) or a surgical procedure (CABG) designed to improve myocardial blood flow	• Coronary revascularisation procedure
Heart failure	Presentation of the patient for an urgent, unscheduled clinic/office/emergency department visit or hospital admission, with a primary diagnosis of heart failure (new episode or worsening of existing heart failure)	• Heart failure hospitalisation<br>• Urgent heart failure visit
Acute pancreatitis	The diagnosis of acute pancreatitis requires two of the following three	• Acute pancreatitis<br>• Mild

		features:<br>(1) abdominal pain **consistent** with acute pancreatitis (acute onset of a persistent, severe, epigastric pain often radiating to the back)<br>(2) serum lipase activity (and/or amylase activity) at least three times greater than the upper limit of normal<br>(3) characteristic findings of acute pancreatitis on imaging	• Moderately severe<br>• Severe	

**AE and SAE recording**
• The investigator will record all relevant AE/SAE information in the CRF.<br>• The investigator will attempt to establish a diagnosis of the event based on signs, symptoms, and/or other clinical information. In such cases, the diagnosis (not the individual signs/symptoms) will be documented as the AE/SAE.<br>• When an AE/SAE occurs, it is the responsibility of the investigator to review all documentation (e.g. hospital progress notes, laboratory and diagnostics reports) related to the event.<br>• There may be instances when copies of source documents (e.g. medical records) for certain cases are requested by Novo Nordisk. In such cases, all subject identifiers, with the exception of the subject number, will be redacted on the copies of the source documents before submission to Novo Nordisk.<br>• For all non-serious AEs the applicable forms should be signed when the event is resolved or at the end of the trial at the latest. For sign-off of SAE related forms refer to "SAE reporting via paper CRF" later in this section.<br>• Novo Nordisk products used as concomitant medication: if an AE is considered to have a causal relationship with a Novo Nordisk marketed product used as concomitant medication in the trial, it is important that the suspected relationship is reported to Novo Nordisk, e.g. in the alternative aetiology section on the safety information form. Novo Nordisk may need to report this AE to relevant regulatory authorities.

**Assessment of severity**
The investigator will assess intensity for each event reported during the trial and assign it to one of the following categories:<br>• **Mild**: An event that is easily tolerated by the subject, causing minimal discomfort and not interfering with everyday activities.<br>• **Moderate**: An event that causes sufficient discomfort and interferes with normal everyday activities.<br>• **Severe**: An event that prevents normal everyday activities.<br>Note: Severe is a category used for rating the intensity of an event; and both an AE and SAE can be assessed as severe. An event is defined as 'serious' when it meets at least one of the outcomes described in the definition of an SAE and not when it is rated as severe.

**Assessment of causality**
The investigator is obligated to assess the relationship between trial product and the occurrence of each AE/SAE.<br><br>Relationship between an AE/SAE and the relevant trial product*(s)* should be assessed as:<br>• Probable - Good reason and sufficient documentation to assume a causal relationship.<br>• Possible - A causal relationship is conceivable and cannot be dismissed.<br>• Unlikely - The event is most likely related to aetiology other than the trial product.

Alternative aetiology, such as underlying disease(s), concomitant medication, and other risk factors, as well as the temporal relationship of the event to trial product administration will be considered and investigated.

The investigator should use the IB for the assessment. For each AE/SAE, the investigator must document in the medical records that he/she has reviewed the AE/SAE and has provided an assessment of causality.

There may be situations in which an SAE has occurred and the investigator has minimal information to include in the initial report. However, **it is important that the investigator always makes an assessment of causality for every event before the initial transmission of the SAE data**.

The investigator may change his/her opinion of causality in light of follow-up information and send a follow-up report with the updated causality assessment.

The causality assessment is one of the criteria used when determining regulatory reporting requirements.

Final outcome

The investigator will select the most appropriate outcome:

- **Recovered/resolved:** The subject has fully recovered, or by medical or surgical treatment the condition has returned to the level observed at the first trial-related activity after the subject signed the informed consent.
- **Recovering/resolving:** The condition is improving and the subject is expected to recover from the event. This term is only applicable if the subject has completed the trial or has died from another AE.
- **Recovered/resolved with sequelae:** The subject has recovered from the condition, but with lasting effect due to a disease, injury, treatment or procedure. If a sequelae meets an SAE criterion, the AE must be reported as an SAE.
- **Not recovered/not resolved:** The condition of the subject has not improved and the symptoms are unchanged or the outcome is not known.
- **Fatal:** This term is only applicable if the subject died from a condition related to the reported AE. Outcomes of other reported AEs in a subject before he/she died should be assessed as "recovered/resolved", "recovering/resolving", "recovered/resolved with sequelae" or "not recovered/not resolved". An AE with a fatal outcome must be reported as an SAE.
- **Unknown:** This term is only applicable if the subject is lost to follow-up.

Follow-up of AE and SAE

The investigator is obligated to perform or arrange for the conduct of supplemental measurements and/or evaluations as medically indicated or as requested by Novo Nordisk to elucidate the nature and/or causality of the AE or SAE as fully as possible (e.g. severe hypersensitivity reactions). This may include additional laboratory tests (e.g. skin prick test) or investigations, histopathological examinations, or consultation with other health care professionals.
If a subject dies during participation in the trial or during a recognised follow-up period, the investigator should provide Novo Nordisk with a copy of autopsy report including histopathology.

New or updated information will be recorded in the CRF.

SAE reporting via electronic CRF

- Relevant forms (AE and safety information form) must be completed in the CRF.
- For reporting and sign-off timelines (see box below).
- If the CRF is unavailable for more than 24 hours, then the site will use the paper AE form and if the CRF is unavailable for more than 5 calendar days then the site will use the paper safety information form (see box below).
- The site will enter the SAE data into the CRF as soon as it becomes available, see Section 9.2.1.
- After the trial is completed at a given site, the CRF will be decommissioned to prevent the entry of new data or

changes to existing data. If a site receives a report of a new SAE from a subject or receives updated data on a previously reported SAE after CRF decommission, then the site can report this information on a paper AE and safety information form (see box below) or to Novo Nordisk by telephone.

SAE reporting via paper CRF

- Relevant CRF forms (AE and safety information form) must be forwarded to Novo Nordisk either by fax, e-mail or courier.
- Initial notification via telephone is acceptable, although it does not replace the need for the investigator to complete the AE and safety information form within the designated reporting time frames (as illustrated in Figure 9-1):
  - AE form within 24 hours.
  - Safety information form within 5 calendar days.
  - Both forms must be signed within 7 calendar days.

Contact details for SAE reporting can be found in the investigator trial master file.

Appendix 5 Contraceptive guidance and collection of pregnancy information

It must be recorded in the CRF whether female subjects are of childbearing potential.

Definitions

Woman of Childbearing Potential (WOCBP)

A woman is considered fertile following menarche and until becoming postmenopausal unless permanently sterile.

Women in the following categories are not considered WOCBP

1. Premenarcheal
2. Premenopausal female with one of the following:
   - Documented hysterectomy
   - Documented bilateral salpingectomy
   - Documented bilateral oophorectomy

   Note: Documentation can come from the site personnel's review of subject's medical records, medical examination or medical history interview.
3. Postmenopausal female

   - A postmenopausal state is defined as no menses for 12 months without an alternative medical cause. A high Follicle Stimulating Hormone (FSH) level in the postmenopausal range may be used to confirm a postmenopausal state in women not using hormonal contraception or Hormonal Replacement Therapy (HRT). However, in the absence of 12 months of amenorrhea, a single FSH measurement is insufficient.
   - Females on HRT and whose menopausal status is in doubt will be required to use one of the non-hormonal highly effective contraception methods if they wish to continue their HRT during the trial. Otherwise, they must discontinue HRT to allow confirmation of postmenopausal status before trial enrolment.

Contraception guidance

Male subjects
No contraception measures are required for male subjects as the risk of teratogenicity/fetotoxicity caused by transfer of semaglutide in seminal fluid is unlikely.

Female subjects
Female subjects of childbearing potential are eligible to participate if they agree to use methods of contraception consistently and correctly as described in table(s) below:

Table 11-5 Highly effective contraceptive methods

**Highly effective contraceptive methods that are user dependent** [a and b]<br>Failure rate of <1% per year when used consistently and correctly.
Combined (oestrogen and progestogen containing) hormonal contraception associated with inhibition of ovulation<br>• oral<br>• intravaginal<br>• transdermal
Progestogen only hormonal contraception associated with inhibition of ovulation<br>• oral<br>• injectable
**Highly effective methods that are user independent** [b]
Implantable progestogen only hormonal contraception associated with inhibition of ovulation<br>• Intrauterine Device<br>• Intrauterine hormone-releasing System<br>• Bilateral tubal occlusion
**Vasectomised partner**<br>A vasectomised partner is a highly effective contraception method provided that the partner is the sole male sexual partner of the WOCBP and the absence of sperm has been confirmed. If not, an additional highly effective method of contraception should be used.
**Sexual abstinence** [b]<br>Sexual abstinence is considered a highly effective method only if defined as refraining from heterosexual intercourse during the entire period of risk associated with the trial product. The reliability of sexual abstinence needs to be evaluated in relation to the duration of the trial and the preferred and usual lifestyle of the subject.
**Notes:**<br>[a] Typical use failure rates may differ from < 1 % per year, if not used consistently and correctly. Use should be consistent with local regulations regarding the used of contraceptive methods for subjects participating in clinical trials.<br>[b] Contraception should be utilised during the treatment period and for at least 49 days after the last dose of trial product.

In certain cases, it is accepted to use double barrier methods (a condom combined with an occlusive cap (e.g. diaphragm) with/without the use of spermicide). This should only be allowed in females with:

1) known intolerance to the highly effective methods mentioned in Table 11-5 -or where the use of any of the listed highly effective contraceptive measures are contraindicated in the individual subject, and/or

2) if the risk of initiating treatment with a specific highly effective method outweighs the benefit for the female.

Justification for accepting double barrier method should be at the discretion of the investigator taking into consideration his/hers knowledge about the female's obesity history, concomitant illness, concomitant medication and observed AEs. The justification must be stated in the medical records.

Pregnancy testing

- WOCBP should only be included after a negative highly sensitive urine pregnancy test.
- Urine pregnancy testing should be performed at every site visit (every 4-8 weeks) during the treatment period, at end of treatment and after the 7 weeks off-drug follow-up period according to the flow chart.
- Additional urine pregnancy testing should be performed at monthly intervals during the treatment period, if required locally (Appendix 5).
- Pregnancy testing should be performed whenever a menstrual cycle is missed or when pregnancy is otherwise suspected.

Collection of pregnancy information

Female subjects who become pregnant

- Investigator will collect pregnancy information on any female subject, who becomes pregnant while participating in this trial.
- Information will be recorded on the appropriate form and submitted to Novo Nordisk within 14 calendar days of learning of a subject's pregnancy.
- Subject will be followed to determine the outcome of the pregnancy. The investigator will collect follow-up information on subject and neonate, which will be forwarded to Novo Nordisk. Generally, follow-up will not be required for longer than 1 month beyond the delivery date.
- Any termination of pregnancy will be reported, regardless of foetal status (presence or absence of anomalies) or indication for procedure.
- While pregnancy itself is not considered to be an AE or SAE, any pregnancy complication or elective termination of a pregnancy will be reported as an AE or SAE.
- A spontaneous abortion is always considered to be an SAE and will be reported as such.
- Any SAE occurring as a result of a post-trial pregnancy which is considered possibly/probably related to the trial product by the investigator will be reported to Novo Nordisk as described in Appendix 4. While the investigator is not obligated to actively seek this information in former subjects, he or she may learn of an SAE through spontaneous reporting.

Any female subject who becomes pregnant while participating in the trial will discontinue trial product.

Appendix 6 Technical complaints: Definition and procedures for recording, evaluation, follow-up and reporting

**Technical complaint definition**
• A technical complaint is any written, electronic or oral communication that alleges product (medicine or device) defects. The technical complaint may be associated with an AE, but does not concern the AE itself.

Examples of technical complaints:

- Problems with the physical or chemical appearance of trial products (e.g. discoloration, particles or contamination).
- Problems with packaging material including labelling.
- Problems related to medical devices (e.g. to the injection mechanism, dose setting mechanism, dose button or interface between the pen-injector and the needle).

**Time period for detecting technical complaints**
All technical complaints, which occur from the time of receipt of the product at trial site until the time of the last usage of the product, must be collected for products predefined on the technical complaint form.

**Reporting of technical complaints to Novo Nordisk**
Contact details (fax, e-mail and address) for Customer Complaint Center – refer to Attachment I<br>Technical complaints must be reported on a separate technical complaint form:<br>1. One technical complaint form must be completed for each affected DUN<br>2. If DUN is not available, a technical complaint form for each batch, code or lot number must be completed

**Timelines for reporting of technical complaints to Novo Nordisk**
The investigator must complete the technical complaint form in the CRF within the timelines specified in Figure 9-3.<br><br>If the CRF is unavailable or when reporting a technical complaint that is not subject related, the information must be provided on a paper form by fax, e-mail or courier to Customer Complaint Center, Novo Nordisk, within the same timelines as stated above. When the CRF becomes available again, the investigator must enter the information on the technical complaint form in the CRF.

**Follow-up of technical complaints**
The investigator is responsible for ensuring that new or updated information will be recorded on the originally completed form.

**Collection, storage and shipment of technical complaint samples**
The investigator must collect the technical complaint sample and all associated parts that were packed in the same DUN and notify the monitor within 5 calendar days of obtaining the sample at trial site. The sample and all associated parts must be sent as soon as possible to Customer Complaint Center, Novo Nordisk, together with a copy of the completed technical complaint form. The technical complaint sample should contain the batch, code or lot number and, if available, the DUN. If the technical complaint sample is unobtainable, the reason must be stated on the technical complaint form. If several samples are shipped in one shipment, the sample and the corresponding technical complaint

form should be kept together.<br>Storage of the technical complaint sample must be done in accordance with the conditions prescribed for the product.

**Reporting of technical complaints for Novo Nordisk products not included in technical complaint form**
Technical complaints on Novo Nordisk products not included in the technical complaint form should be reported to local Novo Nordisk affiliate with a reference to trial ID.

All technical complaints are handled by Customer Complaint Center at Novo Nordisk. Only technical complaints related to adverse events will be included in the CTR.

Appendix 7 Monitoring of calcitonin

Background

Treatment with GLP-1 RAs has been shown to be associated with thyroid C-cell changes in rodents but not in non-human primates. The human relevance of this finding is unknown. However, based on the findings in rodents, monitoring of serum calcitonin (a sensitive biomarker for C-cell activation) is currently being performed in clinical trials with semaglutide.

While there is general agreement on the clinical interpretation of substantially elevated calcitonin levels (> 100 ng/L) as likely indicative of C-cell neoplasia, the interpretation of values between upper normal range (5.0 and 8.4 ng/L for women and men, respectively) and 100 ng/L is less clear with regards to indication of disease.

There are several known confounding factors affecting calcitonin levels, e.g.:

- renal dysfunction
- tobacco use
- autoimmune thyroiditis
- several drug classes (e.g. proton pump inhibitors, beta-blockers, H_2-blockers and glucocorticoids)

Physiology of C-cell activation in various clinical conditions and in different patient populations (i.e. with various comorbidities) is poorly understood. There may be various clinical conditions not identified so far which mildly or moderately affect calcitonin secretion by C-cells.

Calcitonin monitoring

A blood sample will be drawn at pre-specified trial visits for measurement of calcitonin.

In case a subject has a calcitonin value ≥ 10 ng/L, the algorithm outlined in Figure 11-1 and described below should be followed. The algorithm applies for all calcitonin values in the trial.

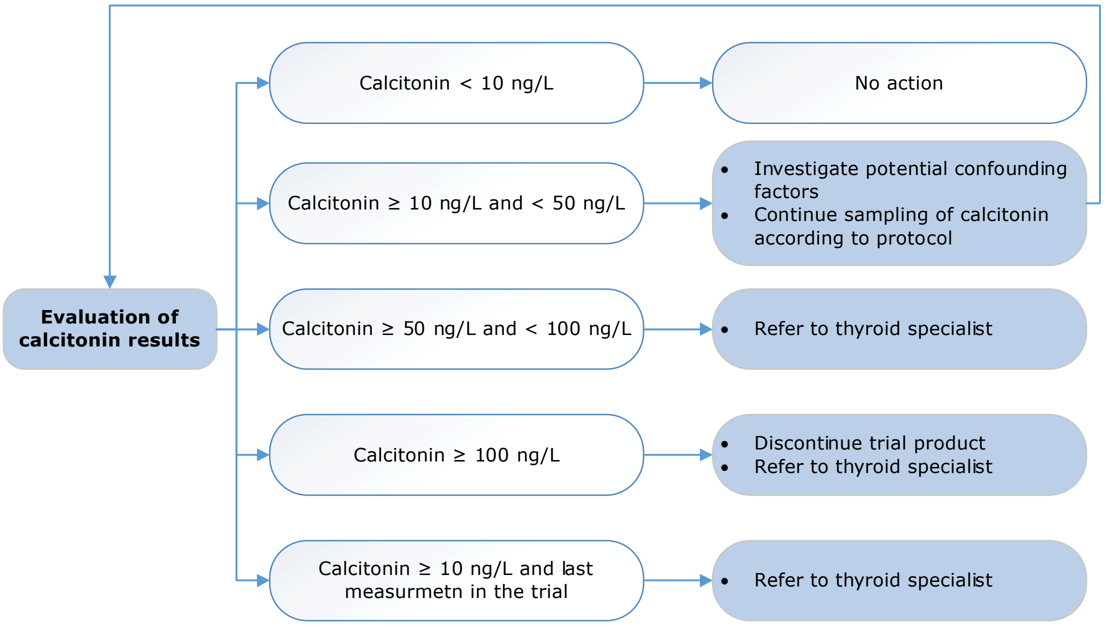

Figure 11-1 Flow of calcitonin monitoring

Calcitonin ≥ 100 ng/L
Action: The subject (even if a screen failure) must immediately be referred to a thyroid specialist for further evaluation and the trial product must be discontinued (see Section 8.1). The subject should remain in the trial; however, all medications suspected to relate to this condition must be discontinued until diagnosis has been established.

Background: These values were found in 9 (0.15%) of a population of 5817 patients with thyroid nodular disease [83] . All of these patients were diagnosed with MTC, resulting in a positive predictive value of 100%.

Diagnostic evaluation should include:

- thyroid ultrasound examination
- fine needle aspiration of any nodules > 1 cm
- potentially, surgery with neck dissection

In case a subject is diagnosed with MTC, it is common clinical practice to explore the family history of MTC or MEN2 and perform a genetic test for RET proto-oncogene mutation.

Calcitonin ≥ 50 and < 100 ng/L
Action: The subject (even if a screen failure) should be referred to a thyroid specialist for further evaluation. The subject should remain in the trial and can continue on trial product.

Background: These values were found in 8 (0.14%) of the population of 5817 patients with thyroid nodular disease [83]. Two of these subjects were diagnosed with MTC and two were diagnosed with C cell hyperplasia, resulting in a positive predictive value of a C-cell anomaly of 50%.

Diagnostic evaluation should include:

- thyroid ultrasound examination
- if available, and if there are no contraindications, a pentagastrin stimulation test should be done. For subjects with positive pentagastrin stimulation test, surgery should be considered.
- if pentagastrin stimulation test is not available, thyroid ultrasound and fine needle aspiration biopsy may add important clinical information about the need for surgery.

Calcitonin ≥ 10 and < 50 ng/L

Action: The subject can continue in the trial on trial product. Continue sampling of calcitonin according to the protocol.

If the subject is a screen failure, or if the value is from the last sample taken in the trial, the subject should be referred to a thyroid specialist for further evaluation.

Background: Calcitonin values from 20–50 ng/L were found in up to 1% of subjects of the population of 5817 patients with thyroid nodular disease [83]. The predictive value of a C-cell anomaly for this calcitonin level was 8.3%. However, the likelihood of having a medullary carcinoma >1 cm with calcitonin in this range is extremely low.

For calcitonin values between 10-20 ng/L Costante et al.[83] identified 216 (3.7%) patients. One patient out of the 216 had a subsequent basal (unstimulated) calcitonin value of 33 ng/L, and had C-cell hyperplasia at surgery. Two other studies used a cut-off of calcitonin > 10 ng/L to screen for C-cell disease, but they do not provide sufficient information on patients with basal CT > 10 and < 20 ng/L to allow conclusions [84, 85].

Appendix 8 Country-specific requirements

The below list is not an exhausted list of country specific requirements. The list will only be updated in case of a global protocol amendment.

For Spain: Double-barrier contraceptive methods are not considered as 'highly effective' methods according to the EMA guideline. Therefore double-barriers methods could not be considered as valid in Spain to assess exclusion criterion number 28.

12 References

1. The European Parliament and the Council of the European Council. Directive 2001/20/EC of the European Parliament and of the Council of 4 April 2001 on the approximation of the laws, regulations and administrative provisions of the Member States relating to the implementation of good clinical practice in the conduct of clinical trials on medicinal products for human use, article 11. Official Journal of the European Communities. 01 May 2001.
2. The American Society for Metabolic and Bariatric Surgery, The Obesity Society, The American Society of Bariatric Physicians and the American Association of Clinical Endocrinologists. Obesity is a Disease: Leading Obesity Groups Agree (Joint Press Release). 19 June 2013.
3. Mechanick JI, Garber AJ, Handelsman Y, Garvey WT. American Association of Clinical Endocrinologists' position statement on obesity and obesity medicine. Endocr Pract. 2012;18(5):642-8.
4. Toplak H, Woodward E, Yumuk V, Oppert JM, Halford JC, Frühbeck G. 2014 EASO Position Statement on the Use of Anti-Obesity Drugs. Obes Facts. 2015;8(3):166-74.
5. Fruhbeck G, Toplak H, Woodward E, Yumuk V, Maislos M, Oppert JM, et al. Obesity: the gateway to ill health - an EASO position statement on a rising public health, clinical and scientific challenge in Europe. Obes Facts. 2013;6(2):117-20.
6. Apovian CM, Aronne LJ, Bessesen DH, McDonnell ME, Murad MH, Pagotto U, et al. Pharmacological management of obesity: an endocrine Society clinical practice guideline. J Clin Endocrinol Metab. 2015;100(2):342-62.
7. Ferguson C, David S, Divine L, Kahan S, Gallagher C, Gooding M, et al. Obesity Drug Outcome Measures. A Consensus Report of Considerations Regarding Pharmacologic Intervention. Avaliable from: https://publichealth.gwu.edu/pdf/obesitydrugmeasures.pdf. 2012.
8. Sumithran P, Prendergast LA, Delbridge E, Purcell K, Shulkes A, Kriketos A, et al. Long-term persistence of hormonal adaptations to weight loss. N Engl J Med. 2011;365(17):1597-604.
9. Schwartz A, Doucet E. Relative changes in resting energy expenditure during weight loss: a systematic review. Obes Rev. 2010;11(7):531-47.
10. Pasman WJ, Saris WH, Westerterp-Plantenga MS. Predictors of weight maintenance. Obes Res. 1999;7(1):43-50.
11. Dombrowski SU, Knittle K, Avenell A, Araujo-Soares V, Sniehotta FF. Long term maintenance of weight loss with non-surgical interventions in obese adults: systematic review and meta-analyses of randomised controlled trials. BMJ. 2014;348:g2646.
12. Brethauer SA, Chand B, Schauer PR. Risks and benefits of bariatric surgery: current evidence. Cleve Clin J Med. 2006;73(11):993-1007.
13. Schauer PR, Ikramuddin S, Gourash W, Ramanathan R, Luketich J. Outcomes after laparoscopic Roux-en-Y gastric bypass for morbid obesity. Ann Surg. 2000;232(4):515-29.
14. Yumuk V, Tsigos C, Fried M, Schindler K, Busetto L, Micic D, et al. European Guidelines for Obesity Management in Adults. Obes Facts. 2015;8(6):402-24.
15. Haslam D. Weight management in obesity - past and present. Int J Clin Pract. 2016;70(3):206-17.

16. Kelly T, Yang W, Chen CS, Reynolds K, He J. Global burden of obesity in 2005 and projections to 2030. Int J Obes (Lond). 2008;32(9):1431-7.
17. Ogden CL, Carroll MD, Kit BK, Flegal KM. Prevalence of childhood and adult obesity in the United States, 2011-2012. JAMA. 2014;311(8):806-14.
18. Stevens GA, Singh GM, Lu Y, Danaei G, Lin JK, Finucane MM, et al. National, regional, and global trends in adult overweight and obesity prevalences. Popul Health Metr. 2012;10(1):22.
19. NCD Risk Factor Collaboration. Trends in adult body-mass index in 200 countries from 1975 to 2014: a pooled analysis of 1698 population-based measurement studies with 19·2 million participants. Lancet. 2016;387(10026):1377-96.
20. Finkelstein EA, DiBonaventura M, Burgess SM, Hale BC. The costs of obesity in the workplace. J Occup Environ Med. 2010;52(10):971-6.
21. Van Nuys K, Globe D, Ng-Mak D, Cheung H, Sullivan J, Goldman D. The association between employee obesity and employer costs: evidence from a panel of U.S. employers. Am J Health Promot. 2014;28(5):277-85.
22. Wang YC, Pamplin J, Long MW, Ward ZJ, Gortmaker SL, Andreyeva T. Severe Obesity In Adults Cost State Medicaid Programs Nearly $8 Billion In 2013. Health Aff (Millwood). 2015;34(11):1923-31.
23. Cusi K. Role of obesity and lipotoxicity in the development of nonalcoholic steatohepatitis: pathophysiology and clinical implications. Gastroenterology. 2012;142(4):711-25.e6.
24. Guh DP, Zhang W, Bansback N, Amarsi Z, Birmingham CL, Anis AH. The incidence of co-morbidities related to obesity and overweight: a systematic review and meta-analysis. BMC Public Health. 2009;9:88.
25. Pischon T, Boeing H, Hoffmann K, Bergmann M, Schulze MB, Overvad K, et al. General and abdominal adiposity and risk of death in Europe. N Engl J Med. 2008;359(20):2105-20.
26. Berghofer A, Pischon T, Reinhold T, Apovian CM, Sharma AM, Willich SN. Obesity prevalence from a European perspective: a systematic review. BMC Public Health. 2008;8:200.
27. Masters RK, Reither EN, Powers DA, Yang YC, Burger AE, Link BG. The impact of obesity on US mortality levels: the importance of age and cohort factors in population estimates. Am J Public Health. 2013;103(10):1895-901.
28. Arnold M, Pandeya N, Byrnes G, Renehan AG, Stevens GA, Ezzati M, et al. Global burden of cancer attributable to high body-mass index in 2012: a population-based study. Lancet Oncol. 2015;16(1):36-46.
29. World Health Organization. Obesity: Preventing and Managing the Global Epidemic. Report of a WHO Consultation. World Health Organisation Geneva, Switzerland. 2000.
30. Thomsen M, Nordestgaard BG. Myocardial Infarction and Ischemic Heart Disease in Overweight and Obesity With and Without Metabolic Syndrome. JAMA internal medicine. 2013;174(1):15-22.
31. Eckel RH, Kahn SE, Ferrannini E, Goldfine AB, Nathan DM, Schwartz MW, et al. Obesity and type 2 diabetes: what can be unified and what needs to be individualized? Journal of clinical endocrinology and metabolism. 2011;96(6):1654-63.
32. Khaodhiar L, Cummings S, Apovian CM. Treating diabetes and prediabetes by focusing on obesity management. Curr Diab Rep. 2009;9(5):348-54.

33. Wheaton AG, Perry GS, Chapman DP, Croft JB. Sleep disordered breathing and depression among U.S. adults: National Health and Nutrition Examination Survey, 2005 -2008. Sleep. 2012;35(4):461-7.
34. Prospective Studies Collaboration, Whitlock G, Lewington S, Sherliker P, Clarke R, Emberson J, et al. Body-mass index and cause-specific mortality in 900 000 adults: collaborative analyses of 57 prospective studies. Lancet. 2009;373(9669):1083-96.
35. Must A, Spadano J, Coakley EH, Field AE, Colditz G, Dietz WH. The disease burden associated with overweight and obesity. JAMA. 1999;282(16):1523-9.
36. Church TS, Kuk JL, Ross R, Priest EL, Biltoft E, Biltoff E, et al. Association of cardiorespiratory fitness, body mass index, and waist circumference to nonalcoholic fatty liver disease. Gastroenterology. 2006;130(7):2023-30.
37. Blagojevic M, Jinks C, Jeffery A, Jordan KP. Risk factors for onset of osteoarthritis of the knee in older adults: a systematic review and meta-analysis. Osteoarthritis Cartilage. 2010;18(1):24-33.
38. Garner RE, Feeny DH, Thompson A, Bernier J, McFarland BH, Huguet N, et al. Bodyweight, gender, and quality of life: a population-based longitudinal study. Qual Life Res. 2012;21(5):813-25.
39. Kearns B, Ara R, Young T, Relton C. Association between body mass index and health-related quality of life, and the impact of self-reported long-term conditions - cross-sectional study from the south Yorkshire cohort dataset. BMC Public Health. 2013;13:1009.
40. Wei M, Kampert JB, Barlow CE, Nichaman MZ, Gibbons LW, Paffenbarger RS, et al. Relationship between low cardiorespiratory fitness and mortality in normal-weight, overweight, and obese men. JAMA. 1999;282(16):1547-53.
41. Tuomilehto J, Lindstrom J, Eriksson JG, Valle TT, Hamalainen H, Ilanne-Parikka P, et al. Prevention of type 2 diabetes mellitus by changes in lifestyle among subjects with impaired glucose tolerance. N Engl J Med. 2001;344(18):1343-50.
42. Knowler WC, Barrett-Connor E, Fowler SE, Hamman RF, Lachin JM, Walker EA, et al. Reduction in the incidence of type 2 diabetes with lifestyle intervention or metformin. N Engl J Med. 2002;346(6):393-403.
43. Li G, Zhang P, Wang J, Gregg EW, Yang W, Gong Q, et al. The long-term effect of lifestyle interventions to prevent diabetes in the China Da Qing Diabetes Prevention Study: a 20-year follow-up study. Lancet. 2008;371(9626):1783-9.
44. Diabetes Prevention Program Research Group, Knowler WC, Fowler SE, Hamman RF, Christophi CA, Hoffman HJ, et al. 10-year follow-up of diabetes incidence and weight loss in the Diabetes Prevention Program Outcomes Study. Lancet. 2009;374(9702):1677-86.
45. Foster GD, Borradaile KE, Sanders MH, Millman R, Zammit G, Newman AB, et al. A Randomized Study on the Effect of Weight Loss on Obstructive Sleep Apnea Among Obese Patients With Type 2 Diabetes The Sleep AHEAD Study. Archives of Internal Medicine. 2009;169(17):1619-26.
46. Felson DT, Zhang Y, Anthony JM, Naimark A, Anderson JJ. Weight loss reduces the risk for symptomatic knee osteoarthritis in women. The Framingham Study. Ann Intern Med. 1992;116(7):535-9.
47. Dengo AL, Dennis EA, Orr JS, Marinik EL, Ehrlich E, Davy BM, et al. Arterial destiffening with weight loss in overweight and obese middle-aged and older adults. Hypertension. 2010;55(4):855-61.

48. Dattilo AM, Kris-Etherton PM. Effects of weight reduction on blood lipids and lipoproteins: a meta-analysis. Am J Clin Nutr. 1992;56(2):320-8.
49. Anderson JW, Konz EC. Obesity and disease management: effects of weight loss on comorbid conditions. Obes Res. 2001;9 Suppl 4:326S-34S.
50. Garber AJ, Abrahamson MJ, Barzilay JI, Blonde L, Bloomgarden ZT, Bush MA, et al. American Association of Clinical Endocrinologists' Comprehensive Diabetes Management Algorithm 2013 Consensus Statement - Executive Summary. Endocrine Practice. 2013;19(3):536-47.
51. Mertens IL, Van Gaal LF. Overweight, obesity, and blood pressure: the effects of modest weight reduction. Obes Res. 2000;8(3):270-8.
52. Deitel M, Stone E, Kassam HA, Wilk EJ, Sutherland DJ. Gynecologic-obstetric changes after loss of massive excess weight following bariatric surgery. J Am Coll Nutr. 1988;7(2):147-53.
53. Sjöström L. Review of the key results from the Swedish Obese Subjects (SOS) trial - a prospective controlled intervention study of bariatric surgery. J Intern Med. 2013;273(3):219-34.
54. Gregg EW, Jakicic JM, Blackburn G, Bloomquist P, Bray GA, Clark JM, et al. Association of the magnitude of weight loss and changes in physical fitness with long-term cardiovascular disease outcomes in overweight or obese people with type 2 diabetes: a post-hoc analysis of the Look AHEAD randomised clinical trial. Lancet Diabetes Endocrinol. 2016;4(11):913-21.
55. Afshin A, Forouzanfar MH, Reitsma MB, Sur P, Estep K, Lee A, et al. Health Effects of Overweight and Obesity in 195 Countries over 25 Years. N Engl J Med. 2017;377(1):13-27.
56. Lau J, Bloch P, Schäffer L, Pettersson I, Spetzler J, Kofoed J, et al. Discovery of the Once-Weekly Glucagon-Like Peptide-1 (GLP-1) Analogue Semaglutide. J Med Chem. 2015;58(18):7370-80.
57. Gutzwiller JP, Drewe J, Goke B, Schmidt H, Rohrer B, Lareida J, et al. Glucagon-like peptide-1 promotes satiety and reduces food intake in patients with diabetes mellitus type 2. Am J Physiol. 1999;276(5 Pt 2):R1541-4.
58. Novo Nordisk A/S. Investigator's Brochure, Semaglutide s.c. 2.4 mg, project NN9536, (edition 1). 28 Nov 2017.
59. Sorli C, Harashima SI, Tsoukas GM, Unger J, Karsbøl JD, Hansen T, et al. Efficacy and safety of once-weekly semaglutide monotherapy versus placebo in patients with type 2 diabetes (SUSTAIN 1): a double-blind, randomised, placebo-controlled, parallel-group, multinational, multicentre phase 3a trial. Lancet Diabetes Endocrinol. 2017;5(4):251-60.
60. Aroda VR, Bain SC, Cariou B, Piletič M, Rose L, Axelsen M, et al. Efficacy and safety of once-weekly semaglutide versus once-daily insulin glargine as add-on to metformin (with or without sulfonylureas) in insulin-naive patients with type 2 diabetes (SUSTAIN 4): a randomised, open-label, parallel-group, multicentre, multinational, phase 3a trial. Lancet Diabetes Endocrinol. 2017;5(5):355-66.
61. Ahrén B, Masmiquel L, Kumar H, Sargin M, Karsbøl JD, Jacobsen SH, et al. Efficacy and safety of once-weekly semaglutide versus once-daily sitagliptin as an add-on to metformin, thiazolidinediones, or both, in patients with type 2 diabetes (SUSTAIN 2): a 56-week, double-blind, phase 3a, randomised trial. Lancet Diabetes Endocrinol. 2017;5(5):341-54.

62. Ahmann A, Capehorn M, Charpentier G, Dotta F, Henkel E, Lingvay I, et al. Efficacy and safety of once-weekly semaglutide vs exenatide ER after 56 Weeks in subjects with type 2 diabetes (SUSTAIN 3). European Association for the Study of Diabetes, 52nd meeting 2016, Oral Presentation #1472016.
63. Rodbard H, Lingvay I, Reed J, de la Rosa R, Rose L, Sugimoto D, et al. Efficacy and safety of semaglutide once-weekly vs placebo as add-on to basal insulin alone or in combination with metformin in subjects with type 2 diabetes (SUSTAIN 5) [abstract]. Diabetologia. 2016;59(Suppl 1):364-5.
64. Blundell J, Finlayson G, Axelsen M, Flint A, Gibbons C, Kvist T, et al. Effects of once-weekly semaglutide on appetite, energy intake, control of eating, food preference and body weight in subjects with obesity. Diabetes Obes Metab. 2017.
65. European Medicines Agency. EMA/CHMP/311805/2014; Guideline on clinical evaluation of medicinal products used in weight management. 01 Jan 2017.
66. Food and Drug Administration. FDA Guidance for Industry: Developing products for weight management. 2007.
67. Kidney Disease: Improving Global Outcomes (KDIGO) CKD Work Group. KDIGO 2012 Clinical Practice Guideline for the Evaluation and Management of Chronic Kidney Disease. Kidney Int Suppl. 2013;3(1):1-150.
68. FAO/WHO/UNU. Human energy requirements. Report of a joint FAO/WHO/UNU expert consultation. FAO: food and nutrition technical report series 1. Rome: 2004 2004.
69. Banks PA, Bollen TL, Dervenis C, Gooszen HG, Johnson CD, Sarr MG, et al. Classification of acute pancreatitis-2012: revision of the Atlanta classification and definition s by international consensus. Gut. 2013;62(1):102-11.
70. Dalton M, Finlayson G, Hill A, Blundell J. Preliminary validation and principal components analysis of the Control of Eating Questionnaire (CoEQ) for the experience of food craving. Eur J Clin Nutr. 2015;69(12):1313-7.
71. Kroenke K, Spitzer RL, Williams JB. The PHQ-9 - Validity of a brief depression severity measure. J Gen Intern Med. 2001;16(9):606-13.
72. Posner K, Oquendo MA, Gould M, Stanley B, Davies M. Columbia Classification Algorithm of Suicide Assessment (C-CASA): classification of suicidal events in the FDA's pediatric suicidal risk analysis of antidepressants. Am J Psychiatry. 2007;164(7):1035-43.
73. McEvoy BW. Missing data in clinical trials for weight management. J Biopharm Stat. 2016;26(1):30-6.
74. Carpenter JR, Roger JH, Kenward MG. Analysis of longitudinal trials with protocol deviation: a framework for relevant, accessible assumptions, and inference via multiple imputation. J Biopharm Stat. 2013;23(6):1352-71.
75. Sacks FM, Bray GA, Carey VJ, Smith SR, Ryan DH, Anton SD, et al. Comparison of weight-loss diets with different compositions of fat, protein, and carbohydrates. N Engl J Med. 2009;360(9):859-73.
76. World Medical Association. Declaration of Helsinki - Ethical Principles for Medical Research Involving Human Subjects. Last amended by the 64th WMA General Assembly, Fortaleza, Brazil. 1 Oct 2013.
77. ICH Harmonised Tripartite Guideline. Guideline for Good Clinical Practice E6(R2), Current Step 4 version. 09 Nov 2016.

78. Committee for Medicinal Products for Human Use (CHMP). Guideline on clinical evaluation of medicinal products used in weight management. EMA/CHMP/311805/2014. 2016.
79. International Committee of Medical Journal Editors. Recommendations for the Conduct, Reporting, Editing and Publication of Scholarly Work in Medical Journals; current version available at www.icmje.org.
80. De Angelis C, Drazen JM, Frizelle FA, Haug C, Hoey J, Horton R, et al. Clinical trial registration: a statement from the International Committee of Medical Journal Editors. N Engl J Med. 2004;351(12):1250-1.
81. U.S. Food and Drug Administration. Food and Drug Administration Amendments Act of 2007 as amended by the Final Rule "Clinical Trials Registration and Results Information Submission". 21 September 2016.
82. The European Parliament and the Council of the European Council. Regulation (EC) No 726/2004 of the European Parliament and of the Council of 31 March 2004 laying down Community procedures for the authorisation and supervision of medicinal products for human and veterinary use and establishing a European Medicines Agency, article 57. 30 April 2004.
83. Costante G, Meringolo D, Durante C, Bianchi D, Nocera M, Tumino S, et al. Predictive value of serum calcitonin levels for preoperative diagnosis of medullary thyroid carcinoma in a cohort of 5817 consecutive patients with thyroid nodules. J Clin Endocrinol Metab. 2007;92(2):450-5.
84. Scheuba C, Kaserer K, Moritz A, Drosten R, Vierhapper H, Bieglmayer C, et al. Sporadic hypercalcitoninemia: clinical and therapeutic consequences. Endocr Relat Cancer. 2009;16(1):243-53.
85. Verga U, Ferrero S, Vicentini L, Brambilla T, Cirello V, Muzza M, et al. Histopathological and molecular studies in patients with goiter and hypercalcitoninemia: reactive or neoplastic C-cell hyperplasia? Endocr Relat Cancer. 2007;14(2):393-403.

Global and country key Novo Nordisk staff

Attachments I and II (if applicable) to the protocol are located in the Trial Master File.

Content: Global key staff and Country key staff
