# Supplementary material for: Two-year effects of semaglutide in adults with overweight or obesity: the STEP 5 trial
Source: Nat Med. 2022 Oct 10;28(10):2083–91. doi: 10.1038/s41591-022-02026-4 (PMC9556320; doi:10.1038/s41591-022-02026-4)
Supplement: Supplementary file 4 — ICMJE forms. [file 41591_2022_2026_MOESM4_ESM.pdf]

ICMJE DISCLOSURE FORM

**Date:**	7/11/2022
**Your Name:**	Meena Bhatta
**Manuscript Title:**	Two-year effect of semaglutide 2.4 mg once-weekly in adults with overweight or obesity (STEP 5): a randomized, double-blind, placebo controlled, phase 3 trial
**Manuscript Number (if known):**	Click or tap here to enter text.

In the interest of transparency, we ask you to disclose all relationships/activities/interests listed below that are related to the content of your manuscript. "Related" means any relation with for-profit or not-for-profit third parties whose interests may be affected by the content of the manuscript. Disclosure represents a commitment to transparency and does not necessarily indicate a bias. If you are in doubt about whether to list a relationship/activity/interest, it is preferable that you do so.

The author's relationships/activities/interests should be defined broadly. For example, if your manuscript pertains to the epidemiology of hypertension, you should declare all relationships with manufacturers of antihypertensive medication, even if that medication is not mentioned in the manuscript.

In item #1 below, report all support for the work reported in this manuscript without time limit. For all other items, the time frame for disclosure is the past 36 months.

		**Name all entities with whom you have this relationship or indicate none (add rows as needed)**	**Specifications/Comments (e.g., if payments were made to you or to your institution)**
		Time frame: Since the initial planning of the work	
1	All support for the present manuscript (e.g., funding, provision of study materials, medical writing, article processing charges, etc.) **No time limit for this item.**	☐ None	
		Employee of Novo Nordisk	
			Click the tab key to add additional rows.
		Time frame: past 36 months	
**2**	Grants or contracts from any entity (if not indicated in item #1 above).	☒ **None**	
**3**	Royalties or licenses	☒ **None**	

		Name all entities with whom you have this relationship or indicate none (add rows as needed)	Specifications/Comments (e.g., if payments were made to you or to your institution)
**4**	Consulting fees	☒ **None**	
**5**	Payment or honoraria for lectures, presentations, speakers bureaus, manuscript writing or educational events	☒ **None**	
**6**	Payment for expert testimony	☒ **None**	
**7**	Support for attending meetings and/or travel	☒ **None**	
**8**	Patents planned, issued or pending	☒ **None**	
**9**	Participation on a Data Safety Monitoring Board or Advisory Board	☒ **None**	
**10**	Leadership or fiduciary role in other board, society, committee or advocacy group, paid or unpaid	☒ **None**	

		Name all entities with whom you have this relationship or indicate none (add rows as needed)	Specifications/Comments (e.g., if payments were made to you or to your institution)
**11**	Stock or stock options	☒ **None**	
**12**	Receipt of equipment, materials, drugs, medical writing, gifts or other services	☒ **None**	
**13**	Other financial or non-financial interests	☐ **None**	
		Employee of Novo Nordisk since 2017	

Please place an "X" next to the following statement to indicate your agreement:

☒ I certify that I have answered every question and have not altered the wording of any of the questions on this form.

ICMJE DISCLOSURE FORM

Date: 7/11/2022

Your Name: **Juan Frias**

Manuscript Title: ***Two-year effect of semaglutide 2.4 mg once-weekly in adults with overweight or obesity (STEP 5): a randomized, double-blind, placebo-controlled, phase 3 trial'***

Manuscript Number (if known): Click or tap here to enter text.

In the interest of transparency, we ask you to disclose all relationships/activities/interests listed below that are related to the content of your manuscript. "Related" means any relation with for-profit or not-for-profit third parties whose interests may be affected by the content of the manuscript. Disclosure represents a commitment to transparency and does not necessarily indicate a bias. If you are in doubt about whether to list a relationship/activity/interest, it is preferable that you do so.

The author's relationships/activities/interests should be defined broadly. For example, if your manuscript pertains to the epidemiology of hypertension, you should declare all relationships with manufacturers of antihypertensive medication, even if that medication is not mentioned in the manuscript.

In item #1 below, report all support for the work reported in this manuscript without time limit. For all other items, the time frame for disclosure is the past 36 months.

		**Name all entities with whom you have this relationship or indicate none (add rows as needed)**	**Specifications/Comments (e.g., if payments were made to you or to your institution)**
		Time frame: Since the initial planning of the work	
1	All support for the present manuscript (e.g., funding, provision of study materials, medical writing, article processing charges, etc.) **No time limit for this item.**	☒ None	
			Click the tab key to add additional rows.
		Time frame: past 36 months	

		Name all entities with whom you have this relationship or indicate none (add rows as needed)	Specifications/Comments (e.g., if payments were made to you or to your institution)
**2**	Grants or contracts from any entity (if not indicated in item #1 above).	☐ **None**	
		Akero	Research Support
		AstraZeneca	Research Support
		Boehringer Ingelheim	Research Support
		BMS	Research Support
		89bio	Research Support
		Eli Lilly	Research Support
		Intercept	Research Support
		IONIS	Research Support
		Janssen	Research Support
		Madrigal	Research Support
		Metacrine	Research Support
		Merck	Research Support
		NorthSea Therapeutics	Research Support
		Novartis	Research Support
		Novo Nordisk	Research Support
		Oramed	Research Support
		Pfizer	Research Support
		Poxel	Research Support
		Sanofi	Research Support
**3**	Royalties or licenses	☒ **None**	
**4**	Consulting fees	☐ **None**	
		Akero	
		Altimmune	
		Axcella Health	
		Becton Dickenson	
		Boehringer Ingelheim	
		Carmot Therapeutics	
		Echosens	
		89bio	

		Name all entities with whom you have this relationship or indicate none (add rows as needed)	Specifications/Comments (e.g., if payments were made to you or to your institution)
		Eli Lilly	
		Gilead	
		Intercept	
		Metacrine	
		Merck	
		Novo Nordisk	
		Pfizer	
		Sanofi	
**5**	Payment or honoraria for lectures, presentations, speakers bureaus, manuscript writing or educational events	☐ **None**	
		Eli Lilly	
		Sanofi	
**6**	Payment for expert testimony	☒ **None**	
**7**	Support for attending meetings and/or travel	☒ **None**	
**8**	Patents planned, issued or pending	☒ **None**	
**9**	Participation on a Data Safety Monitoring Board or Advisory Board	☐ **None**	
		Akero	
		Altimmune	
		Axcella Health	
		Becton Dickenson	
		Boehringer Ingelheim	
		Carmot Therapeutics	

		Name all entities with whom you have this relationship or indicate none (add rows as needed)	Specifications/Comments (e.g., if payments were made to you or to your institution)
		Echosens	
		89bio	
		Eli Lilly	
		Gilead	
		Intercept	
		Metacrine	
		Merck	
		Novo Nordisk	
		Pfizer	
		Sanofi	
10	Leadership or fiduciary role in other board, society, committee or advocacy group, paid or unpaid	☒ None	
11	Stock or stock options	☒ None	
12	Receipt of equipment, materials, drugs, medical writing, gifts or other services	☒ None	
13	Other financial or non-financial interests	☒ None	

Please place an "X" next to the following statement to indicate your agreement:

☒ I certify that I have answered every question and have not altered the wording of any of the questions on this form.

ICMJE DISCLOSURE FORM

Date: 7/7/2022

Your Name: W. Timothy Garvey, MD

Manuscript Title: ***Two-year effect of semaglutide 2.4 mg once-weekly in adults with overweight or obesity (STEP 5): a randomized, double-blind, placebo-controlled, phase 3 trial***

Manuscript Number (if known): Click or tap here to enter text.

In the interest of transparency, we ask you to disclose all relationships/activities/interests listed below that are related to the content of your manuscript. "Related" means any relation with for-profit or not-for-profit third parties whose interests may be affected by the content of the manuscript. Disclosure represents a commitment to transparency and does not necessarily indicate a bias. If you are in doubt about whether to list a relationship/activity/interest, it is preferable that you do so.

The author's relationships/activities/interests should be defined broadly. For example, if your manuscript pertains to the epidemiology of hypertension, you should declare all relationships with manufacturers of antihypertensive medication, even if that medication is not mentioned in the manuscript.

In item #1 below, report all support for the work reported in this manuscript without time limit. For all other items, the time frame for disclosure is the past 36 months.

		**Name all entities with whom you have this relationship or indicate none (add rows as needed)**	**Specifications/Comments (e.g., if payments were made to you or to your institution)**
		**Time frame: Since the initial planning of the work**	
1	All support for the present manuscript (e.g., funding, provision of study materials, medical writing, article processing charges, etc.) No time limit for this item.	☐ None	
		Medical writing assistance from Spirit Medical Communications Group Limited; applies only to current manuscript	No payments to Dr Garvey
			Click the tab key to add additional rows.
		**Time frame: past 36 months**	
**2**	Grants or contracts from any entity (if not indicated in item #1 above).	☐ **None**	
		NIH/NIDDK, American Heart Association	NIH & AHA: For original investigator-initiated research. Unrelated to current manuscript
		Dr Garvey served as site principal investigator for the current clinical trial sponsored by the University of Alabama at Birmingham and funded by Novo Nordisk	
		Dr Garvey served as site principal investigator for other clinical trials all sponsored by the University of Alabama at Birmingham and funded by Novo Nordisk, Pfizer, Lexicon, Eli Lilly, Epitomee	Unrelated to current manuscript

		Name all entities with whom you have this relationship or indicate none (add rows as needed)	Specifications/Comments (e.g., if payments were made to you or to your institution)
**3**	Royalties or licenses	☒ **None**	
**4**	Consulting fees	☒ **None**	
		Dr. Garvey served as a compensated consultant on advisory boards with Novo Nordisk, Boehringer-Ingelheim, and Amgen	
		Dr Garvey served as a volunteer uncompensated consultant on advisory committees for Boehringer Ingelheim, Jazz Pharmaceuticals, Novo Nordisk, and Pfizer.	No honoraria paid; no financial relationship
		Dr Garvey served as compensated consultant on advisory boards for Fractyl Health and Alnylam Pharmaceuticals	May/June 2022
**5**	Payment or honoraria for lectures, presentations, speakers bureaus, manuscript writing or educational events	☒ **None**	
**6**	Payment for expert testimony	☐ **None**	
		Arnold & Porter law firm representing Eisai, Inc	
**7**	Support for attending meetings and/or travel	☐ **None**	
		Travel/Food: Novo Nordisk, Boehringer-Ingelheim, Amgen, Fractyl, Janssen, Valeritas, Amarin, Lexicon, Merck	To advisory boards or group receptions at scientific meetings
**8**	Patents planned, issued or pending	☒ **None**	

		Name all entities with whom you have this relationship or indicate none (add rows as needed)	Specifications/Comments (e.g., if payments were made to you or to your institution)
**9**	Participation on a Data Safety Monitoring Board or Advisory Board	☒ **None**	
**10**	Leadership or fiduciary role in other board, society, committee or advocacy group, paid or unpaid	☐ **None**	
		Board member and executive committee member in the American Association of Clinical Endocrinology	Unpaid. Not active after June 1, 2022
		Advisory Board for Milken Institute	501c3 nonprofit; no payments
**11**	Stock or stock options	☐ **None**	
		Publically-traded equities managed by investment firm--- Novartis, Eli Lilly, Pfizer, Ionis, Merck, Bristol-Myers-Squibb	Each worth < $25,000
**12**	Receipt of equipment, materials, drugs, medical writing, gifts or other services	☒ **None**	
**13**	Other financial or non-financial interests	☒ **None**	

Please place an "X" next to the following statement to indicate your agreement:

☒ I certify that I have answered every question and have not altered the wording of any of the questions on this form.

ICMJE DISCLOSURE FORM

Date: 5/13/2022

Your Name: Silvio Buscemi

Manuscript Title: ***Two-year effect of semaglutide 2.4 mg once-weekly in adults with overweight or obesity (STEP 5): a randomized, double-blind, placebo-controlled, phase 3 trial***

Manuscript Number (if known): Click or tap here to enter text.

In the interest of transparency, we ask you to disclose all relationships/activities/interests listed below that are related to the content of your manuscript. "Related" means any relation with for-profit or not-for-profit third parties whose interests may be affected by the content of the manuscript. Disclosure represents a commitment to transparency and does not necessarily indicate a bias. If you are in doubt about whether to list a relationship/activity/interest, it is preferable that you do so.

The author's relationships/activities/interests should be defined broadly. For example, if your manuscript pertains to the epidemiology of hypertension, you should declare all relationships with manufacturers of antihypertensive medication, even if that medication is not mentioned in the manuscript.

In item #1 below, report all support for the work reported in this manuscript without time limit. For all other items, the time frame for disclosure is the past 36 months.

		Name all entities with whom you have this relationship or indicate none (add rows as needed)	Specifications/Comments (e.g., if payments were made to you or to your institution)
**Time frame: Since the initial planning of the work**			
1	All support for the present manuscript (e.g., funding, provision of study materials, medical writing, article processing charges, etc.) **No time limit for this item.**	☐ None	
		NovoNordisk	
			Click the tab key to add additional rows.
**Time frame: past 36 months**			
**2**	Grants or contracts from any entity (if not indicated in item #1 above).	☐ **None**	
		Member of the Global Expert Panel of the NovoNordisk Seletc Study	
		PI in different NovoNordisk trials in the field of obesity and diabetes	
**3**	Royalties or licenses	☒ **None**	

		Name all entities with whom you have this relationship or indicate none (add rows as needed)	Specifications/Comments (e.g., if payments were made to you or to your institution)
**4**	Consulting fees	☒ **None**	
**5**	Payment or honoraria for lectures, presentations, speakers bureaus, manuscript writing or educational events	☒ **None**	
**6**	Payment for expert testimony	☒ **None**	
**7**	Support for attending meetings and/or travel	☒ **None**	
**8**	Patents planned, issued or pending	☒ **None**	
**9**	Participation on a Data Safety Monitoring Board or Advisory Board	☒ **None**	
**10**	Leadership or fiduciary role in other board, society, committee or advocacy group, paid or unpaid	☒ **None**	

		Name all entities with whom you have this relationship or indicate none (add rows as needed)	Specifications/Comments (e.g., if payments were made to you or to your institution)
**11**	Stock or stock options	☒ **None**	
**12**	Receipt of equipment, materials, drugs, medical writing, gifts or other services	☒ **None**	
**13**	Other financial or non-financial interests	☒ **None**	

Please place an "X" next to the following statement to indicate your agreement:

☐ I certify that I have answered every question and have not altered the wording of any of the questions on this form.

ICMJE DISCLOSURE FORM

Date:___May, 13th, 2021____
Your Name:___Esteban Jódar GImeno_______________________________
Manuscript Title:
Manuscript number (if known):___________________________________

In the interest of transparency, we ask you to disclose all relationships/activities/interests listed below that are related to the content of your manuscript. "Related" means any relation with for-profit or not-for-profit third parties whose interests may be affected by the content of the manuscript. Disclosure represents a commitment to transparency and does not necessarily indicate a bias. If you are in doubt about whether to list a relationship/activity/interest, it is preferable that you do so.

The following questions apply to the author's relationships/activities/interests as they relate to the current manuscript only.

The author's relationships/activities/interests should be defined broadly. For example, if your manuscript pertains to the epidemiology of hypertension, you should declare all relationships with manufacturers of antihypertensive medication, even if that medication is not mentioned in the manuscript.

In item #1 below, report all support for the work reported in this manuscript without time limit. For all other items, the time frame for disclosure is the past 36 months.

		**Name all entities with whom you have this relationship or indicate none (add rows as needed)**	**Specifications/Comments (e.g., if payments were made to you or to your institution)**
**Time frame: Since the initial planning of the work**			
1	All support for the present manuscript (e.g., funding, provision of study materials, medical writing, article processing charges, etc.) **No time limit for this item.**	____None	Lilly
**Time frame: past 36 months**			
2	Grants or contracts from any entity (if not indicated in item #1 above).	____None	Amgen, Boehringer, AstraZeneca, FAES, Janssen, Lilly, MSD, Novo Nordisk, Pfizer, Sanofi, Shire and UCB
3	Royalties or licenses	_X_None	

4	Consulting fees	____None	Amgen, AstraZeneca, FAES, Helios-Fresenius, Italfármaco, Lilly, MSD, Mundipharma, Novo Nordisk, UCB and Viatris
5	Payment or honoraria for lectures, presentations, speakers bureaus, manuscript writing or educational events	____None	Amgen, Asofarma, Astellas, AstraZeneca, Bayer, Boehringuer, BMS, FAES, Lilly, MSD, Mundipharma, Novo Nordisk, Technofarma, UCB and Viatris
6	Payment for expert testimony	_X_None	
7	Support for attending meetings and/or travel	____None	Amgen, AstraZeneca, BMS, Novo Nordisk, UCB
8	Patents planned, issued or pending	_X_None	
9	Participation on a Data Safety Monitoring Board or Advisory Board	____None	Amgen, AstraZeneca, FAES, Lilly, MSD, Mundipharma, Novo Nordisk, UCB and Viatris
10	Leadership or fiduciary role in other board, society, committee or advocacy group, paid or unpaid	X_None	
11	Stock or stock options	_X_None	
12	Receipt of equipment, materials, drugs, medical writing, gifts or other services	_X_None	
13	Other financial or non-financial interests	_X_None	

Please place an "X" next to the following statement to indicate your agreement:

X_ I certify that I have answered every question and have not altered the wording of any of the questions on this form.

ICMJE DISCLOSURE FORM

**Date:**	5/11/2022
**Your Name:**	Kristian Kandler
**Manuscript Title:**	***Two-year effect of semaglutide 2.4 mg once-weekly in adults with overweight or obesity (STEP 5): a randomized, double-blind, placebo-controlled, phase 3 trial***
**Manuscript Number (if known):**	Click or tap here to enter text.

In the interest of transparency, we ask you to disclose all relationships/activities/interests listed below that are related to the content of your manuscript. "Related" means any relation with for-profit or not-for-profit third parties whose interests may be affected by the content of the manuscript. Disclosure represents a commitment to transparency and does not necessarily indicate a bias. If you are in doubt about whether to list a relationship/activity/interest, it is preferable that you do so.

The author's relationships/activities/interests should be defined broadly. For example, if your manuscript pertains to the epidemiology of hypertension, you should declare all relationships with manufacturers of antihypertensive medication, even if that medication is not mentioned in the manuscript.

In item #1 below, report all support for the work reported in this manuscript without time limit. For all other items, the time frame for disclosure is the past 36 months.

		Name all entities with whom you have this relationship or indicate none (add rows as needed)	Specifications/Comments (e.g., if payments were made to you or to your institution)
**Time frame: Since the initial planning of the work**			
1	All support for the present manuscript (e.g., funding, provision of study materials, medical writing, article processing charges, etc.) **No time limit for this item.**	☒ None	
			Click the tab key to add additional rows.
**Time frame: past 36 months**			
**2**	Grants or contracts from any entity (if not indicated in item #1 above).	☒ **None**	
		Employee at Novo Nordisk	
**3**	Royalties or licenses	☒ **None**	

		Name all entities with whom you have this relationship or indicate none (add rows as needed)	Specifications/Comments (e.g., if payments were made to you or to your institution)
**4**	Consulting fees	☒ **None**	
**5**	Payment or honoraria for lectures, presentations, speakers bureaus, manuscript writing or educational events	☒ **None**	
**6**	Payment for expert testimony	☒ **None**	
**7**	Support for attending meetings and/or travel	☒ **None**	
**8**	Patents planned, issued or pending	☒ **None**	
**9**	Participation on a Data Safety Monitoring Board or Advisory Board	☒ **None**	
**10**	Leadership or fiduciary role in other board, society, committee or advocacy group, paid or unpaid	☒ **None**	

		Name all entities with whom you have this relationship or indicate none (add rows as needed)	Specifications/Comments (e.g., if payments were made to you or to your institution)
**11**	Stock or stock options	☒ **None**	
**12**	Receipt of equipment, materials, drugs, medical writing, gifts or other services	☒ **None**	
**13**	Other financial or non-financial interests	☒ **None**	

Please place an "X" next to the following statement to indicate your agreement:

☒ I certify that I have answered every question and have not altered the wording of any of the questions on this form.

ICMJE DISCLOSURE FORM

Date: 5/11/2022

Your Name: Sean Wharton

Manuscript Title: Two-year effect of semaglutide 2.4 mg once-weekly in adults with overweight or obesity (STEP 5): a randomized, double-blind, placebo controlled, phase 3 trial

Manuscript Number (if known): Click or tap here to enter text.

In the interest of transparency, we ask you to disclose all relationships/activities/interests listed below that are related to the content of your manuscript. "Related" means any relation with for-profit or not-for-profit third parties whose interests may be affected by the content of the manuscript. Disclosure represents a commitment to transparency and does not necessarily indicate a bias. If you are in doubt about whether to list a relationship/activity/interest, it is preferable that you do so.

The author's relationships/activities/interests should be defined broadly. For example, if your manuscript pertains to the epidemiology of hypertension, you should declare all relationships with manufacturers of antihypertensive medication, even if that medication is not mentioned in the manuscript.

In item #1 below, report all support for the work reported in this manuscript without time limit. For all other items, the time frame for disclosure is the past 36 months.

		Name all entities with whom you have this relationship or indicate none (add rows as needed)	Specifications/Comments (e.g., if payments were made to you or to your institution)
		**Time frame: Since the initial planning of the work**	
1	All support for the present manuscript (e.g., funding, provision of study materials, medical writing, article processing charges, etc.) **No time limit for this item.**	☐ None	
		Medical writing and editorial assistance funded by Novo Nordisk	
			Click the tab key to add additional rows.
		**Time frame: past 36 months**	
**2**	Grants or contracts from any entity (if not indicated in item #1 above).	☐ **None**	
		Novo Nordisk, Bausch Health Canada	
**3**	Royalties or licenses	☒ **None**	

		Name all entities with whom you have this relationship or indicate none (add rows as needed)	Specifications/Comments (e.g., if payments were made to you or to your institution)
**4**	Consulting fees	☒ **None**	
**5**	Payment or honoraria for lectures, presentations, speakers bureaus, manuscript writing or educational events	☐ **None**	
		Eli Lilly, Novo Nordisk Inc., Boehringer Ingelheim, Bausch Health Canada	
**6**	Payment for expert testimony	☒ **None**	
**7**	Support for attending meetings and/or travel	☐ **None**	
		Novo Nordisk	
**8**	Patents planned, issued or pending	☒ **None**	
**9**	Participation on a Data Safety Monitoring Board or Advisory Board	☐ **None**	
		Eli Lilly, Boehringer Ingelheim, Novo Nordisk	
**10**	Leadership or fiduciary role in other board, society, committee or advocacy group, paid or unpaid	☐ **None**	
		The Obesity Society, Obesity Canada	

		Name all entities with whom you have this relationship or indicate none (add rows as needed)	Specifications/Comments (e.g., if payments were made to you or to your institution)
**11**	Stock or stock options	☒ **None**	
**12**	Receipt of equipment, materials, drugs, medical writing, gifts or other services	☐ **None**	
		Novo Nordisk – Obesity Care Specialist for CBT provided for patient care	
**13**	Other financial or non-financial interests	☒ **None**	

Please place an "X" next to the following statement to indicate your agreement:

☒ I certify that I have answered every question and have not altered the wording of any of the questions on this form.

ICMJE DISCLOSURE FORM

**Date:**	7/9/2022
**Your Name:**	Prof Rachel L Batterham
**Manuscript Title:**	***Two-year effect of semaglutide 2.4 mg once-weekly in adults with overweight or obesity (STEP 5): a randomized, double-blind, placebo-controlled, phase 3 trial'***
**Manuscript Number (if known):**	Click or tap here to enter text.

In the interest of transparency, we ask you to disclose all relationships/activities/interests listed below that are related to the content of your manuscript. "Related" means any relation with for-profit or not-for-profit third parties whose interests may be affected by the content of the manuscript. Disclosure represents a commitment to transparency and does not necessarily indicate a bias. If you are in doubt about whether to list a relationship/activity/interest, it is preferable that you do so.

The author's relationships/activities/interests should be defined broadly. For example, if your manuscript pertains to the epidemiology of hypertension, you should declare all relationships with manufacturers of antihypertensive medication, even if that medication is not mentioned in the manuscript.

In item #1 below, report all support for the work reported in this manuscript without time limit. For all other items, the time frame for disclosure is the past 36 months.

		Name all entities with whom you have this relationship or indicate none (add rows as needed)	Specifications/Comments (e.g., if payments were made to you or to your institution)
**Time frame: Since the initial planning of the work**			
1	All support for the present manuscript (e.g., funding, provision of study materials, medical writing, article processing charges, etc.) **No time limit for this item.**	☒ None	
			Click the tab key to add additional rows.
**Time frame: past 36 months**			

		Name all entities with whom you have this relationship or indicate none (add rows as needed)	Specifications/Comments (e.g., if payments were made to you or to your institution)
**2**	Grants or contracts from any entity (if not indicated in item #1 above).	☐ **None**	
		National Institute for Health and Care Research (NIHR)	Grant funding to institution
		Sir Jules Thorn Biomedical Trust	Grant funding to institution
		Rosetrees Trust	Grant funding to institution
		European Commission Human Brain Project Grant	Grant funding to institution
		Novo Nordisk	Grant funding to institution for commercial trial
		Rhythm	Funding to institution for commercial trial
**3**	Royalties or licenses	☒ **None**	
**4**	Consulting fees	☐ **None**	
		Novo Nordisk	Personal
		Boehringer-Ingelheim	Personal
		ViiV	Personal
		GWLW-01	Personal
**5**	Payment or honoraria for lectures, presentations, speakers bureaus, manuscript writing or educational events	☐ **None**	
		Novo Nordisk	Personal
		ViiV	Personal
		Eli Lilly	Personal
		Gila Therapeutics Ltd	Personal
		International Medical Press	Personal
**6**	Payment for expert testimony	☒ **None**	

		Name all entities with whom you have this relationship or indicate none (add rows as needed)	Specifications/Comments (e.g., if payments were made to you or to your institution)
7	Support for attending meetings and/or travel	☐ None	
		ViiV	Personal
		Novo Nordisk	Personal
		Pfizer	Personal
8	Patents planned, issued or pending	☒ None	
9	Participation on a Data Safety Monitoring Board or Advisory Board	☐ None	
		Novo Nordisk	Personal
		Pfizer	Personal
		Eli Lilly	Personal
		GLW-01	Personal
		Gila Therapeutics Ltd	Personal
10	Leadership or fiduciary role in other board, society, committee or advocacy group, paid or unpaid	☐ None	
		Royal College of Physicians London<br>• Special Advisor<br>• Chair of the RCP Advisory Group on Weight, Health and Nutrition<br>• Member of Advisory Group on Health Inequalities	Unpaid
		Association for the Study of Obesity Trustee	Unpaid
		Co-Chair of NHS England's Clinical Advisory Group for Tier 3 & Tier 4 weight management services	Unpaid
		Member of the NICE Obesity update Guideline Development Group	Unpaid
		Clinical Committee member for the European Society of Endocrinology	Unpaid
		Chair of the Steering Group and Trustee for the patient charity, Obesity Empowerment Network UK (since 2019)	Unpaid

		Name all entities with whom you have this relationship or indicate none (add rows as needed)	Specifications/Comments (e.g., if payments were made to you or to your institution)
11	Stock or stock options	☒ **None**	
12	Receipt of equipment, materials, drugs, medical writing, gifts or other services	☐ **None**	
		Novo Nordisk	Provision of IMP and placebo for clinical trial to institution<br>Centrifuge for clinical trial to institution
13	Other financial or non-financial interests	☒ **None**	

Please place an "X" next to the following statement to indicate your agreement:

☒ I certify that I have answered every question and have not altered the wording of any of the questions on this form.

ICMJE DISCLOSURE FORM

**Date:**	7/29/2022
**Your Name:**	Thomas A. Wadden
**Manuscript Title:**	Two-year effect of semaglutide 2.4 mg once weekly in adults with overweight or obesity (STEP 5)
**Manuscript Number (if known):**	Click or tap here to enter text.

In the interest of transparency, we ask you to disclose all relationships/activities/interests listed below that are related to the content of your manuscript. "Related" means any relation with for-profit or not-for-profit third parties whose interests may be affected by the content of the manuscript. Disclosure represents a commitment to transparency and does not necessarily indicate a bias. If you are in doubt about whether to list a relationship/activity/interest, it is preferable that you do so.

The author's relationships/activities/interests should be defined broadly. For example, if your manuscript pertains to the epidemiology of hypertension, you should declare all relationships with manufacturers of antihypertensive medication, even if that medication is not mentioned in the manuscript.

In item #1 below, report all support for the work reported in this manuscript without time limit. For all other items, the time frame for disclosure is the past 36 months.

		**Name all entities with whom you have this relationship or indicate none (add rows as needed)**	**Specifications/Comments (e.g., if payments were made to you or to your institution)**
		**Time frame: Since the initial planning of the work**	
1	All support for the present manuscript (e.g., funding, provision of study materials, medical writing, article processing charges, etc.) **No time limit for this item.**	☐ Novo Nordisk. The University of Pennsylvania received funding from Novo Nordisk to support my participation as principal investigator for this study at the Penn site.	
			Click the tab key to add additional rows.
		**Time frame: past 36 months**	
**2**	Grants or contracts from any entity (if not indicated in item #1 above).	☐ **No other grants related to this study**	
**3**	Royalties or licenses	☐ **None**	

		Name all entities with whom you have this relationship or indicate none (add rows as needed)	Specifications/Comments (e.g., if payments were made to you or to your institution)
**4**	Consulting fees	☐ Service on advisory boards for Novo Nordisk And WW (formally Weight Watchers).	Payments were made to me in both cases.
**5**	Payment or honoraria for lectures, presentations, speakers bureaus, manuscript writing or educational events	☐ **None**	
**6**	Payment for expert testimony	☐ **None**	
**7**	Support for attending meetings and/or travel	☐ **None**	
**8**	Patents planned, issued or pending	☐ **None**	
**9**	Participation on a Data Safety Monitoring Board or Advisory Board	☐ **None**	
**10**	Leadership or fiduciary role in other board, society, committee or advocacy group, paid or unpaid	☐ **None**	

		Name all entities with whom you have this relationship or indicate none (add rows as needed)	Specifications/Comments (e.g., if payments were made to you or to your institution)
**11**	Stock or stock options	☐ **None**	
**12**	Receipt of equipment, materials, drugs, medical writing, gifts or other services	☐ **None**	
**13**	Other financial or non-financial interests	☒ **None**	

Please place an "X" next to the following statement to indicate your agreement:

☒ I certify that I have answered every question and have not altered the wording of any of the questions on this form.

ICMJE DISCLOSURE FORM

**Date:**	7/14/2022
**Your Name:**	DR GEORGIA RIGAS
**Manuscript Title:**	Two-year effect of semaglutide 2.4 mg once-weekly in adults with overweight or obesity (STEP 5): a randomized, double-blind, placebo controlled, phase 3 trial
**Manuscript Number (if known):**	Click or tap here to enter text.

In the interest of transparency, we ask you to disclose all relationships/activities/interests listed below that are related to the content of your manuscript. "Related" means any relation with for-profit or not-for-profit third parties whose interests may be affected by the content of the manuscript. Disclosure represents a commitment to transparency and does not necessarily indicate a bias. If you are in doubt about whether to list a relationship/activity/interest, it is preferable that you do so.

The author's relationships/activities/interests should be defined broadly. For example, if your manuscript pertains to the epidemiology of hypertension, you should declare all relationships with manufacturers of antihypertensive medication, even if that medication is not mentioned in the manuscript.

In item #1 below, report all support for the work reported in this manuscript without time limit. For all other items, the time frame for disclosure is the past 36 months.

		**Name all entities with whom you have this relationship or indicate none (add rows as needed)**	**Specifications/Comments (e.g., if payments were made to you or to your institution)**
**Time frame: Since the initial planning of the work**			
1	All support for the present manuscript (e.g., funding, provision of study materials, medical writing, article processing charges, etc.) No time limit for this item.	☒ None	
			Click the tab key to add additional rows.
**Time frame: past 36 months**			
**2**	Grants or contracts from any entity (if not indicated in item #1 above).	☒ **None**	
**3**	Royalties or licenses	☒ **None**	

		Name all entities with whom you have this relationship or indicate none (add rows as needed)	Specifications/Comments (e.g., if payments were made to you or to your institution)
4	Consulting fees	☐ None	
		Johnson & Johnson, Australia	
		Nestle HealthScience, Australia	
		Reshape HealthSciences, Australia	
		iNova Pharmaceuticals, Australia	
		NovoNordisk Pharmaceuticals, Australia	
		NovoNordisk Pharmaceuticals, Global	
5	Payment or honoraria for lectures, presentations, speakers bureaus, manuscript writing or educational events	☐ None	
		Reshape HealthSciences, Australia	
		iNova Pharmaceuticals, Australia	
		NovoNordisk Pharmaceuticals, Australia	
		NovoNordisk Pharmaceuticals, Global	
		NovoNordisk Pharmaceuticals, Global	
		NovoNordisk Pharmaceuticals, Saudi Arabia	
		NovoNordisk Pharmaceuticals, Taiwan	
		NovoNordisk Pharmaceuticals, New Zealand	
		W.L. Gore & Associates, Australia	
		Merk, Sharpe & Dohme, Australia	
		Johnson & Johnson, Australia	
		MDbriefcase, Australia	
		MDbriefcase, Global	
		International Medical Press, Global	
		WedMD, Global	
		Medscape Education, Global	
		NSW Health, Australia	
		Australian Ministry of Health	
		Aspire, Saudi Arabia	
6	Payment for expert testimony	☒ None	
7	Support for attending meetings and/or travel	☐ None	
		NovoNordisk Australia:	educational grant (travel and conference registration fee) to attend national obesity conferences
		NovoNordisk Global:	educational grant (travel and conference registration fee) to attend international obesity conferences

		Name all entities with whom you have this relationship or indicate none (add rows as needed)	Specifications/Comments (e.g., if payments were made to you or to your institution)
8	Patents planned, issued or pending	☒ None	
9	Participation on a Data Safety Monitoring Board or Advisory Board	☐ None	
		Johnson & Johnson, Australia	Advisory board
		Nestle HealthScience, Australia	Advisory board
		Reshape HealthSciences, Australia	Advisory board
		iNova Pharmaceuticals, Australia	Advisory board
		NovoNordisk Pharmaceuticals, Australia	Advisory board
		NovoNordisk Pharmaceuticals, Global	Advisory board
10	Leadership or fiduciary role in other board, society, committee or advocacy group, paid or unpaid	☐ None	
		Royal Australian College of General Practice	Founding Chair, Obesity Management Specific Interest Network
		The Obesity Collective	GP representative advocacy group
		Pharmaceutical Benefits Advisory Committee, Australian Government	Medical advisor
11	Stock or stock options	☒ None	
12	Receipt of equipment, materials, drugs, medical writing, gifts or other services	☐ None	
		iNova Pharmaceuticals	Medication samples for patients
		NovoNordisk Pharmaceuticals, Australia	Medication samples for patients
13	Other financial or non-financial interests	☒ None	

Please place an "X" next to the following statement to indicate your agreement:

☒ I certify that I have answered every question and have not altered the wording of any of the questions on this form.

ICMJE DISCLOSURE FORM

**Date:**	8/1/2022
**Your Name:**	Louise Nygaard Christensen
**Manuscript Title:**	Two-year effect of semaglutide 2.4 mg once-weekly in adults with overweight or obesity (STEP 5): a randomized, double-blind, placebo controlled, phase 3 trial
**Manuscript Number (if known):**	1

In the interest of transparency, we ask you to disclose all relationships/activities/interests listed below that are related to the content of your manuscript. "Related" means any relation with for-profit or not-for-profit third parties whose interests may be affected by the content of the manuscript. Disclosure represents a commitment to transparency and does not necessarily indicate a bias. If you are in doubt about whether to list a relationship/activity/interest, it is preferable that you do so.

The author's relationships/activities/interests should be defined broadly. For example, if your manuscript pertains to the epidemiology of hypertension, you should declare all relationships with manufacturers of antihypertensive medication, even if that medication is not mentioned in the manuscript.

In item #1 below, report all support for the work reported in this manuscript without time limit. For all other items, the time frame for disclosure is the past 36 months.

		**Name all entities with whom you have this relationship or indicate none (add rows as needed)**	**Specifications/Comments (e.g., if payments were made to you or to your institution)**
**Time frame: Since the initial planning of the work**			
1	All support for the present manuscript (e.g., funding, provision of study materials, medical writing, article processing charges, etc.) **No time limit for this item.**	☒ None	
			Click the tab key to add additional rows.
**Time frame: past 36 months**			
**2**	Grants or contracts from any entity (if not indicated in item #1 above).	☒ **None**	
**3**	Royalties or licenses	☒ **None**	

		Name all entities with whom you have this relationship or indicate none (add rows as needed)	Specifications/Comments (e.g., if payments were made to you or to your institution)
**4**	Consulting fees	☒ **None**	
**5**	Payment or honoraria for lectures, presentations, speakers bureaus, manuscript writing or educational events	☒ **None**	
**6**	Payment for expert testimony	☒ **None**	
**7**	Support for attending meetings and/or travel	☒ **None**	
**8**	Patents planned, issued or pending	☒ **None**	
**9**	Participation on a Data Safety Monitoring Board or Advisory Board	☒ **None**	
**10**	Leadership or fiduciary role in other board, society, committee or advocacy group, paid or unpaid	☒ **None**	

		Name all entities with whom you have this relationship or indicate none (add rows as needed)	Specifications/Comments (e.g., if payments were made to you or to your institution)
**11**	Stock or stock options	☒ **None**	
**12**	Receipt of equipment, materials, drugs, medical writing, gifts or other services	☒ **None**	
**13**	Other financial or non-financial interests	☐ **None**	
		Employee of NN	

Please place an "X" next to the following statement to indicate your agreement:

☒ I certify that I have answered every question and have not altered the wording of any of the questions on this form.
